# Gastrointestinal dysfunction in the critically ill: a systematic scoping review and research agenda proposed by the Section of Metabolism, Endocrinology and Nutrition of the European Society of Intensive Care Medicine

**DOI:** 10.1186/s13054-020-02889-4

**Published:** 2020-05-15

**Authors:** Annika Reintam Blaser, Jean-Charles Preiser, Sonja Fruhwald, Alexander Wilmer, Jan Wernerman, Carina Benstoem, Michael P. Casaer, Joel Starkopf, Arthur van Zanten, Olav Rooyackers, Stephan M. Jakob, Cecilia I. Loudet, Danielle E. Bear, Gunnar Elke, Matthias Kott, Ingmar Lautenschläger, Jörn Schäper, Jan Gunst, Christian Stoppe, Leda Nobile, Valentin Fuhrmann, Mette M. Berger, Heleen M. Oudemans-van Straaten, Yaseen M. Arabi, Adam M. Deane, on behalf of the Working Group on Gastrointestinal Function within the Section of Metabolism, Endocrinology and Nutrition (MEN Section) of ESICM, Annika Reintam Blaser, Jean-Charles Preiser, Sonja Fruhwald, Alexander Wilmer, Jan Wernerman, Carina Benstoem, Michael P. Casaer, Joel Starkopf, Arthur van Zanten, Olav Rooyackers, Stephan M. Jakob, Cecilia I. Loudet, Danielle E. Bear, Gunnar Elke, Matthias Kott, Ingmar Lautenschläger, Jörn Schäper, Jan Gunst, Christian Stoppe, Leda Nobile, Valentin Fuhrmann, Mette M. Berger, Heleen M. Oudemans-van Straaten, Yaseen M. Arabi, Adam M. Dean, Karin Amrein, Guillaume Besch, Brenno Belazi Nery de Souza Campos, Elisabeth De Waele, Vincent Fraipont, Teodoro Grau, Kursat Gundogan, Claudia Heidegger, Michael Hiesmayr, Carole Ichai, Mar Juan-Díaz, Kurt Kobke-Jacobsen, Vasiliki Koutsoukou, Marcus Laube, Juan Carlos Montejo González, Zudin Puthucheary, Anne-Francoise Rousseau, Pierre Singer, Sandra N. Stapel, Hakan Tekguc, Carlos Torruco-Sotelo

**Affiliations:** 1 Department of Anaesthesiology and Intensive Care, University of Tartu, Tartu, Estonia; 2 Department of Intensive Care Medicine, Lucerne Cantonal Hospital, Lucerne, Switzerland; 3 Department of Intensive Care, Erasme University Hospital, Université Libre de Bruxelles, Brussels, Belgium; 4 Department of Anaesthesiology and Intensive Care Medicine, Division of Anesthesiology for Cardiovascular Surgery and Intensive Care Medicine, Medical University of Graz, Graz, Austria; 5 Department of Medical Intensive Care, Katholieke Universiteit Leuven, Leuven, Belgium; 6 Department of Anaesthesiology and Intensive Care Medicine, CLINTEC, Karolinska Institutet, Stockholm, Sweden; 7 Department of Intensive Care Medicine, Medical Faculty RWTH Aachen University, Aachen, Germany; 8 Cardiovascular Critical Care & Anesthesia Research and Evaluation (3CARE), Medical Faculty, RWTH Aachen University, Aachen, Germany; 9 Clinical Division and Laboratory of Intensive Care Medicine, Department of Cellular and Molecular Medicine, KU Leuven, Leuven, Belgium; 10 Department of Anaesthesiology and Intensive Care, Tartu University Hospital, Tartu, Estonia; 11 Department of Intensive Care Medicine, Gelderse Vallei Hospital, Ede, The Netherlands; 12 Department of Anesthesiology and Intensive Care, CLINTEC, Karolinska Institutet, Stockholm, Sweden; 13 Department of Intensive Care Medicine, Inselspital, Bern, Switzerland; 14 University of Bern, Bern, Switzerland; 15 Department of Intensive Care, Hospital Interzonal General de Agudos General San Martín, La Plata, Argentina; 16 Departments of Critical Care and Nutrition and Dietetics, Guy’s and St Thomas’ NHS Foundation Trust, London, UK; 17 Centre for Human and Applied Physiological Sciences, King’s College London, London, UK; 18 Department of Anaesthesiology and Intensive Care Medicine, University Medical Center Schleswig-Holstein, Campus Kiel, Kiel, Germany; 19 Department of Anaesthesiology, Universitätsmedizin Göttingen, Göttingen, Germany; 20 Department of Intensive Care Medicine, University Medical Center Hamburg-Eppendorf, Hamburg, Germany; 21 Department of Medicine B, University of Münster, Münster, Germany; 22 Service of Adult Intensive Care Medicine and Burns, Lausanne University Hospital (CHUV), Lausanne, Switzerland; 23 Department of Intensive Care, Amsterdam University Medical Center, VUMC, Amsterdam, The Netherlands; 24 College of Medicine, King Saud bin Abdulaziz University for Health Sciences (KSAU-HS) and King Abdullah International Medical Research Center (KAIMRC), Riyadh, Saudi Arabia; 25 The University of Melbourne, Department of Medicine, Royal Melbourne Hospital, Parkville, Victoria 3050 Australia

**Keywords:** Gastrointestinal function, Gastrointestinal dysfunction, Gastrointestinal failure, Monitoring, Critically ill, Intensive care

## Abstract

**Background:**

Gastrointestinal (GI) dysfunction is frequent in the critically ill but can be overlooked as a result of the lack of standardization of the diagnostic and therapeutic approaches. We aimed to develop a research agenda for GI dysfunction for future research. We systematically reviewed the current knowledge on a broad range of subtopics from a specific viewpoint of GI dysfunction, highlighting the remaining areas of uncertainty and suggesting future studies.

**Methods:**

This systematic scoping review and research agenda was conducted following successive steps: (1) identify clinically important subtopics within the field of GI function which warrant further research; (2) systematically review the literature for each subtopic using PubMed, CENTRAL and Cochrane Database of Systematic Reviews; (3) summarize evidence for each subtopic; (4) identify areas of uncertainty; (5) formulate and refine study proposals that address these subtopics; and (6) prioritize study proposals via sequential voting rounds.

**Results:**

Five major themes were identified: (1) monitoring, (2) associations between GI function and outcome, (3) GI function and nutrition, (4) management of GI dysfunction and (5) pathophysiological mechanisms. Searches on 17 subtopics were performed and evidence summarized. Several areas of uncertainty were identified, six of them needing consensus process. Study proposals ranked among the first ten included: prevention and management of diarrhoea; management of upper and lower feeding intolerance, including indications for post-pyloric feeding and opioid antagonists; acute gastrointestinal injury grading as a bedside tool; the role of intra-abdominal hypertension in the development and monitoring of GI dysfunction and in the development of non-occlusive mesenteric ischaemia; and the effect of proton pump inhibitors on the microbiome in critical illness.

**Conclusions:**

Current evidence on GI dysfunction is scarce, partially due to the lack of precise definitions. The use of core sets of monitoring and outcomes are required to improve the consistency of future studies. We propose several areas for consensus process and outline future study projects.

## Background

**Fig. 1:**
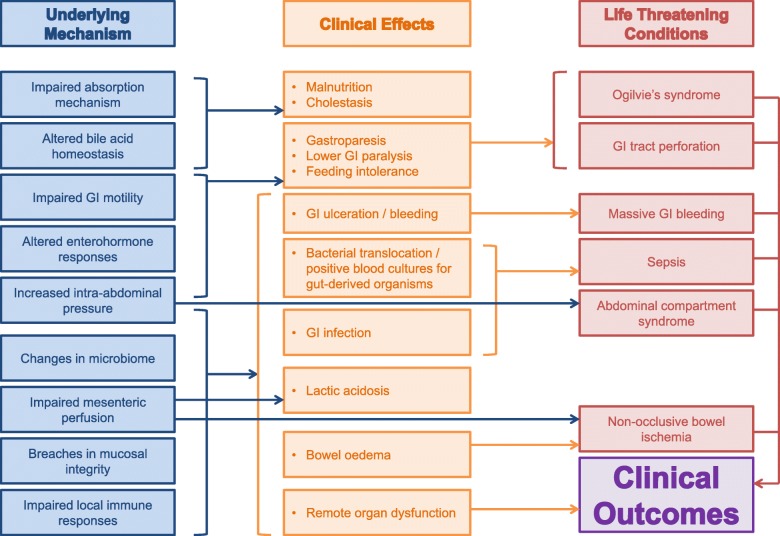
Pathophysiological mechanisms and multi-faceted clinical presentation of GI dysfunction. Critical illness is associated with gastrointestinal (GI)-related (patho)biochemical/physiological mechanisms which can be both cause and consequence of the disease, respectively. These mechanisms again have clinical effects/sequelae that further lead to life-threatening conditions depending on the grade of severity and concordantly affect the clinical outcome. The relationship between these mechanisms (as marked by the arrows) is not linear; they rather occur in parallel and may aggravate each other

Gastrointestinal (GI) dysfunction is frequently seen in critically ill patients and is associated with worse clinical outcomes [1]. GI dysfunction refers broadly to functional impairment of the GI tract that may include disturbances in motility and/or absorption, breaches in mucosal integrity, changes in the microbiome, increased intra-abdominal pressure, impaired mesenteric perfusion infections of the GI tract and other clinical consequences displayed in Fig. 1. These functional impairments may contribute to patient morbidity, may aggravate multi-organ failure and may further deteriorate to life-threatening emergencies (bowel ischaemia, Ogilvie’s syndrome, GI tract perforation, GI bleeding, abdominal compartment syndrome). The underlying pathophysiology of GI dysfunction in critically ill patients comprises several components whose respective influence and relevance are poorly understood. Available monitoring techniques are limited [2], and management options are scarce [3].

We aimed to develop a research agenda for GI dysfunction giving a concise overview of different aspects on GI dysfunction for clinicians and offer a starting point for future research. We did not aim to issue recommendations for clinical practice but rather give a basis for future research that is needed for evidence-based recommendations.

As a first step, we predefined our research themes and subtopics and performed a systematic scoping review to summarize current knowledge in the field (what we know). We address a broad range of subtopics from a specific viewpoint of GI dysfunction selected by a group of experienced ICU physicians specifically dedicated to this topic, also explaining the pathophysiological aspects that need to be further explored before several clinical questions can be answered and monitoring technologies developed. Five major themes related to GI dysfunction were selected a priori: (1) monitoring, (2) associations between GI dysfunction and outcome, (3) GI function and nutrition, (4) management of GI dysfunction and (5) pathophysiological mechanisms. With including experimental research, addressing pathophysiological hypotheses and monitoring, we aimed to provide a broader view and background for future studies rather than a strict assessment of clinical studies in adult critically ill patients.

As next steps, we highlight the key remaining areas of uncertainty (what we do not know) and suggest recommendations for studies/trials (what we need to know). We focus on GI dysfunction as a part of multiple organ dysfunction but do not specifically address interventional management of GI emergencies (e.g. bowel ischaemia or perforation, GI bleeding).

## Methods

The project was initiated by the Section of Metabolism, Endocrinology and Nutrition (MEN) of the European Society of Intensive Care Medicine (ESICM) and endorsed by ESICM. In October 2017, the Working Group (WG) on GI Function within the MEN Section formulated the following steps of the process: (1) identify clinically important subtopics within the a priori identified 5 major themes of GI function which warrant further research, (2) systematically review the literature for each subtopic, (3) summarize evidence for each subtopic, (4) identify areas of uncertainty, (5) formulate and refine study proposals that address these subtopics and (6) prioritize study proposals via sequential voting rounds. The group communicated via e-mail and met four times remotely and twice a year physically during the WG meeting at congresses. The process of voting was discussed and agreed on during the WG meeting in October 2018 and conducted in winter 2019. All MEN Section members were asked for their interest to participate in voting, and all interested members were invited to participate in voting. Voting was conducted in two Delphi rounds, where voting 1 was a shortlisting of all the proposals, and voting 2 was a quality assessment of the 20 highest ranked proposals. Methods in detail and conflicts of interest are presented in Additional file 1.

## Results

Summary of evidence is presented in Table 1 and in Additional file 2, all developed study proposals in Additional file 3, summary on monitoring and motility in Additional file 4, PRISMA (Preferred Reporting Items for Systematic reviews and Meta-Analyses) checklist in Additional file 5 and PRISMA flow diagrams for each systematic review in Additional file 6.

**Table 1:** Summary of evidence in predefined subtopics related to gastrointestinal dysfunction (what we know). More details on the literature behind statements in this table are presented in Supplement 2 Table S3

Subtopic		Study questions	Main observations
Monitoring of GI function			
i.	Clinical assessment	Can GI symptoms* and/or clinical signs be used to monitor GI function in critically ill patients?	- There is no gold standard for monitoring of GI function in critically ill. - GI symptoms and clinical signs may be used, and the number of GI symptoms is associated with increased mortality. - GI symptoms/signs have not been clearly correlated with other objective methods quantifying GI function.
ii.	Imaging	Can imaging be used to monitor GI function in critically ill?	- No validated imaging method for bedside monitoring. - GI ultrasound is promising but requires further study.
iii.	Laboratory (including biomarkers)	Can biomarkers be used to monitor GI function in critically ill?	- No biomarker is validated for clinical use. - Host-, disease- and analytics-related factors may influence potential biomarkers of interest. - Most of the studies assess biomarkers of mesenteric ischaemia or organ dysfunction.
iv.	Absorption of nutrients	Can absorption of nutrients be measured to monitor GI function?	- No method to measure absorption is available for routine clinical use.
v.	Barrier function	Can barrier function be measured to monitor GI function?	- No valid method to measure barrier function is available for routine clinical use.
vi.	Others (including intra-abdominal pressure (IAP))	Which other monitoring methods can be used to monitor GI function?	- IAP can be easily measured and gives a numeric value reflecting abdominal compartment. - Association with GI function is unclear. - Grossly elevated and increasing IAP may necessitate discontinuation or reduction of EN.
Management of GI dysfunction			
vii.	Prokinetics	-Do prokinetics improve upper GI motility in critically ill? - Do prokinetics improve lower GI motility in critically ill? - Does combined treatment of upper and lower GI intolerance improve GI motility in critically ill? - Do prokinetics improve other clinically relevant outcomes?	Gastric emptying: - Erythromycin accelerates gastric emptying and may be superior to metoclopramide. - The effect of combination metoclopramide and erythromycin is sustained for longer than either drug alone. Lower GI dysmotility: insufficient data. Combined treatment of upper and lower GI motility: insufficient data Uncertainty with regards to: - Recommended dose of erythromycin (3 × 100 mg vs 200–250 mg) and therapy duration. - Repeated treatment with gastroprokinetics. - Definition of lower GI intolerance/dysmotility. - Effect on morbidity and mortality.
viii.	Laxatives	Do laxatives improve GI function, morbidity and mortality in critically ill patients?	- Possible benefit of prophylactic therapy (polyethylene glycol, lactulose) regarding time to defaecation, but not regarding complications. - Polyethylene glycol probably better than lactulose, suggested to reduce the incidence of Ogilvie’s syndrome.
ix.	Post-pyloric feeding	Does post-pyloric feeding improve GI function, morbidity and mortality in critically ill patients receiving EN?	- Post-pyloric feeding may reduce the number of patients who develop ventilator-associated pneumonia. - Mostly small studies in patients without feeding intolerance. - Heterogeneity of intervention, i.e. different location of tubes (duodenal and jejunal) pooled.
x.	Others	Which other management improves GI function, morbidity and mortality in critically ill?	- None confirmed in critically ill in general. - In postoperative patients, ERAS protocol and epidural analgesia may improve GI motility. - Beneficial effect of any specific (e.g. restrictive) fluid management strategy on GI function has not been proven.
xi.	GI function and nutrition	Does EN improve GI function, morbidity and mortality in critically ill?	- EN may preserve GI immunity and attenuate proinflammatory changes and bacterial overgrowth. - The quantity of nutrients absorbed with EN during critical illness is uncertain. - EN has not been shown to improve patient-centred outcomes.
Pathophysiological mechanisms in GI dysfunction relevant to the outcome			
xii.	The role of the gut in multiple organ failure	What is the evidence on the role of the GI dysfunction in the development and course of MOF?	Indirect evidence supports a role of GI dysfunction in the development/perpetuation of MODS suggested by associations between the severity of GI dysfunction and organ failures.
xiii.	Microbiome	What is the evidence on the role of the microbiome in GI dysfunction?	Observational data have shown an association between critical illness (severity) and change of the intestinal microbiome as compared to the healthy state (‘dysbiosis’). Change in microbiome is suggested to be associated with GI dysfunction and clinical outcome but has yet to be confirmed by adequately powered studies.
xiv.	Bacterial translocation/mucosal integrity	What is the evidence on bacterial translocation/mucosal integrity in GI dysfunction?	Reported associations between the presence of enteric bacteria or bacterial products in the circulation, presumably related to gut dysfunction and poor outcome. Gut microbiota or related products (e.g. DAMPs in lymphatic ducts, endotoxins in portal blood) may trigger distant organ damage in GI dysfunction.
xv.	GI hormones	What is the evidence that endogenous GI hormones are important in modulating GI dysfunction?	A decrease in the plasma concentration of orexigenic hormones (e.g. ghrelin) and an increase of anorexigenic hormones (e.g. PYY) during the early phase have been observed. No direct correlation with the GI function has been reported.
xvi.	Bile acid signalling	What is the evidence on bile acid signalling in GI dysfunction?	Bile acid signalling as a mechanism of GI dysfunction has not been studied in adult critically ill patients, but increased levels of bile acids in circulation are associated with adverse outcome.
xvii.	Others	What is the evidence on other mechanisms in GI dysfunction?	Bowel oedema and bowel distension have not been studied in critically ill patients. Bowel oedema impaired motility in experimental study.

*DAMP* damage-associated molecular pattern, *EN* enteral nutrition, *ERAS* enhanced recovery after surgery, *IAH* intra-abdominal hypertension, *IAP* intra-abdominal pressure, *FI* feeding intolerance, *GI* gastrointestinal, *MODS* multiple organ dysfunction syndrome, *RCT* randomized controlled trial

*GI symptoms include vomiting/regurgitation, abdominal distension, GI bleeding, diarrhoea and lower GI paralysis [3]. Expanded (if performed/possible to assess) nausea, abdominal pain, absence of bowel sounds, large GRV (> 500 mL/6 h), bowel dilatation (radiological) and bowel wall thickening/bowel oedema (radiological)

## Current knowledge in the field (what we know)

### Monitoring of GI function

Current techniques for monitoring GI dysfunction in critically ill patients are limited [2]. Clinical assessment, often combined with measurement of gastric residual volumes (GRV), is widely used but provides an imprecise assessment of global GI function. Possible techniques to monitor GI function are summarized in Additional file 4, Table S5.

#### Clinical assessment

GI symptoms occur frequently in the critically ill [1]. No single symptom correlates with mortality, whereas an increasing number of concomitant GI symptoms are associated with increasing mortality [1]. There is no agreed and validated scoring system for the assessment of GI dysfunction [3, 4]. The presence of GI bleeding that has been used as a symptom identifying GI dysfunction in multiple organ failure scores [5, 6] is not necessarily related to gut dysfunction, as there are numerous specific causes and therapeutic modalities [7]. Likewise, delayed gastric emptying leading to increased GRV can occur in the absence of intestinal dysfunction. Moreover, using a feeding strategy based on GRV may lack relevance, as it did not decrease the risk of ventilator-associated pneumonia in ventilated medical patients with full enteral nutrition (EN) [8]. Several methods to assess gastric emptying (e.g. scintigraphy, paracetamol absorption test) are mostly used for the purpose of research (Additional file 4, Table S5).

Diarrhoea has been suggested as a marker of malabsorption [9] and could also be considered as a sign of feeding intolerance, but existing evidence is scarce [10].

Clinical symptoms, including diarrhoea, can signal a non-occlusive mesenteric ischaemia (NOMI) that may occur related to early full EN during acute circulatory failure [11].

#### Imaging

Recent studies demonstrated the potential for ultrasound (US) to provide a measure of (1) gastric emptying, (2) bowel peristalsis, (3) bowel diameter, (4) bowel wall thickness and (5) tissue perfusion (US Doppler). The diameter of the gastric antrum measured with US correlates with both GRV and calculations based on CT images [12]. US may also facilitate the placement of feeding tubes and therefore is an imaging technique that could potentially be incorporated into regular abdominal assessment (Additional file 4, Table S5) [13].

#### Biomarkers

Besides blood l-lactate, several novel biomarkers have been proposed [14] (Additional file 4, Table S6). Citrulline levels may represent enterocyte function [15], and citrulline concentrations < 10 μmol/L are associated with increased mortality [16]. Specific aspects and pitfalls for laboratory measurements are summarized (Additional file 4). Despite encouraging preliminary results, several factors may limit the translation of novel biomarkers to clinical practice including (1) the timing of sampling, (2) the extent of surgical damage, (3) the coexistence of other organ dysfunction (e.g. renal), (4) previous gut surgery and length of intact bowel and (5) precision of laboratory technique, threshold values chosen and rapidity of the result [17].

#### Absorption of nutrients

Small cohort studies have demonstrated that absorption of macronutrients is markedly attenuated in the critically ill when compared to health [18–20]. Nutrient analogues or nutrient labelled with an isotope (e.g. 3-*O*-methyl-glucose or ^13^C-glucose) can be administered with enteral nutrition and subsequently sampled from the blood and/or other body fluids to quantify nutrient absorption [18–20]. The results of absorption studies may substantially vary depending on whether markers are administered intragastrically or intraduodenally, especially if gastric emptying is delayed [21]. The duodenal approach will better reflect the actual absorption, whereas the former might be more representative of the actual nutrient (bio)-availability during routine clinical practice.

Utilization of enterally administrated nutrients can be quantified using whole-body balance studies (Additional file 4). However, the precision of this technique requires accurate measurement of intake and output, including output from urine, faeces and drains. Faecal energy loss can be measured as a marker of malabsorption using bomb calorimetry [9], but this method is not widely available and requires the passage of stool, which is infrequent in many critically ill patients [10].

#### Barrier function

GI barrier dysfunction may be caused by (1) loss of enterocyte integrity, (2) increased transcellular/paracellular permeability, (3) loss of mucus layer integrity and (4) impaired mucosal immunity.

The GI barrier can be visualized using electron microscopy [22], but this invasive approach requires tissue biopsy and only quantifies structure at the place and time tissue is obtained. GI barrier function is the net result of a myriad of interactions between the luminal content, the epithelium and the mucosal immune system [23]. Because any or all of these components may be dysregulated in critical illness, no single biomarker (Additional file 4 Table S6) is likely to capture all of these processes to provide a robust summary score.

Double/triple sugar absorption tests are used to determine paracellular permeability in ambulant populations. However, these tests may be affected by GI dysmotility, renal and/or liver impairment, and administration of antibiotics [24], possibly limiting their usefulness in the critically ill.

Quantification of specific enteral bacteria in the blood is possible. However, confirming translocation from the gut lumen as a direct result of gut barrier dysfunction is challenging due to low rates and contamination. In HIV patients, reverse transcription polymerase chain reaction (RT-PCR) of bacterial 16S rDNA has been reported to correlate with lipopolysaccharide blood concentration [25]. This genetic information, however, does not refer to gut-specific bacteria such as *Enterococcus* or *Bacteroides* species. The widely used quantification of endotoxin, corresponding antibodies or binding proteins is neither gut- nor species-specific [26].

#### Other monitoring options

Intra-abdominal pressure (IAP) is readily measurable at the bedside, and increased IAP may be both cause and consequence of GI dysfunction. The definition of intra-abdominal hypertension (IAH) and measurement of IAP is described elsewhere [27]. In a study in mechanically ventilated patients, the presence of IAH in the absence of GI symptoms was not associated with mortality [28].

### GI dysfunction: reporting and outcome

GI dysfunction has been shown to be associated with adverse outcome, even though reported outcomes and their definitions are very variable [4]. The importance of agreement on a minimum collection of essential outcomes within a given field (core outcomes set (COS)) has been recently highlighted [29].

### Management of GI dysfunction

Current management of GI dysfunction mainly relies on treating the underlying causes. In addition, specific therapeutic interventions may be considered, but available options have substantial limitations.

#### GI motility drugs

Current options for treating delayed gastric emptying include drugs such as metoclopramide, erythromycin and domperidone [30] (Additional file 4, Table S7). Domperidone is only available for oral administration, limiting its use in ICU patients. The combination of metoclopramide and erythromycin may have synergistic effects and be superior to either drug alone [31]; however, tachyphylaxis and arrhythmias are the limitations.

A recent meta-analysis reported that prokinetic drugs modestly reduce feeding intolerance (absolute risk reduction 17.3% (95% CI 5–26.8%)) and facilitate the placement of post-pyloric feeding tubes, but had no effect on the development of pneumonia, vomiting and diarrhoea; mortality; or length of hospital stay [32]. An even more recent meta-analysis provided similar results, but erythromycin was the only prokinetic drug to reduce feeding intolerance [33]. Due to concerns about adverse effects of erythromycin, there is a considerable interest in the use of non-antibiotic motilin agonists. The pre-emptive administration of such motilin receptor agonist had a negligible effect on nutrition provision in a recent multicentre clinical trial [34].

Neostigmine is shown effective in colonic paralysis [35] and accordingly used as a treatment for acute colonic pseudo-obstruction (Ogilvie’s syndrome) [36]. To prevent GI paralysis, administration of opioid receptor antagonists, osmotic laxatives (e.g. polyethylene glycol) and stool softeners has been proposed (Additional file 4, Table S7), but demonstrated effect of ‘bowel protocols’ is limited [37].

#### Post-pyloric feeding

Although the gastric route is the preferred method of providing EN, international guidelines include recommendations for the post-pyloric route option in patients at high risk of aspiration or with gastric feeding intolerance [33, 38]. It is important to note that most of the trials and available meta-analyses [38, 39] have not restricted inclusion to patients with signs of GI dysmotility.

#### Systemic management

Apart from systemic conditions such as sepsis and shock, several interventions and specific conditions are considered to contribute to GI dysfunction including (1) intravenous fluid, and plasma glucose and electrolyte concentrations; (2) the use of opioids for analgesia; and (3) untreated intra-abdominal hypertension.

##### Intravenous fluid and plasma electrolytes

There is evidence demonstrating the association between excessive fluid administration and GI dysfunction [40]. At the same time, a recent large RCT in patients undergoing major abdominal surgery reported no signal of fewer episodes of GI dysfunction but a greater number of renal complications with a restrictive approach to perioperative fluid administration [41]. There is currently insufficient evidence to support a restrictive fluid approach on the rationale that it will reduce GI dysfunction.

Hyperglycaemia may slow gastric emptying, whereas hypoglycaemia may accelerate it [42]. Interference of serum electrolyte abnormalities with bowel motility has been suggested [43], but there is insufficient data to target specific plasma electrolyte and glucose thresholds to improve GI function.

##### Pain management and sedation

Stimulation of either opioid or alpha-2 adrenergic receptors may inhibit GI motility [44, 45]. After colorectal surgery, faster recovery of GI motility is achieved with the combination of early postoperative feeding, multimodal analgesic regimens and morphine restriction [46].

##### Intra-abdominal hypertension

IAH can attenuate splanchnic blood flow [47] and exacerbate bowel oedema [48]. No intervention targeting IAH has been shown to improve GI function or outcomes in the critically ill. Effect of IAH on the outcome depends on the severity and dynamics of IAH [49].

### GI function and nutrition

Whilst the dose of nutrition is beyond the scope of this review, prolonged fasting (7 days when compared to 3 days) in the critically ill attenuated nutrient absorption when EN was eventually administered [50]. Early EN may preserve GI immunity, whereas prolonged starvation may cause proinflammatory changes and bacterial overgrowth [51].

On the other hand, a recent RCT demonstrated that early full EN within 24 h in patients with shock was associated with increased risk for non-occlusive bowel ischaemia and colonic pseudo-obstruction [9]. Therefore, the optimal strategy for feeding in shock remains uncertain, but early full feeding may be harmful. Guidelines recommend low-dose early EN (< 48 h of ICU admission) in critically ill patients who are not able to maintain oral intake [33, 52], whereas the extent of absorption of enterally administered nutrients may vary widely [20, 21].

### Pathophysiological mechanisms in GI dysfunction

#### The role of the gut in multiple organ dysfunction syndrome (MODS)

Animal models indicate that altered microbiome (see the ‘Microbiome’ section) during critical illness is associated with loss of intestinal barrier [53]. This then allows the translocation of bacterial products across the mucosa to cause further inflammation and, finally, dysfunction of remote organs (Fig. 1) [54]. For instance, acute lung injury can occur following the release of gut-derived products into the lymphatic vessels and/or directly into the lungs, as shown in a murine model and in humans [55]. In animal models, the ligation of the mesenteric lymph duct prevents the development of lung injury [56]. Likewise, intestinal dysbiosis may lead to hepatic impairment [54]. In the clinical setting, inflammation induced by translocation through disrupted gut epithelium will trigger the administration of fluids and vasopressors. Fluid-induced tissue oedema and mesenteric vasoconstriction may amplify the pathophysiological processes in the gut further and possibly lead to NOMI.

#### Microbiome

The microbiome refers to all of the microbial consortia (both commensal and pathogenic bacteria, viruses and fungi), their genes and gene products (proteins and metabolites), their community structure (distribution, diversity, and evenness) and the particulars of the environment in which they reside. Essential functions of the gut microbiota include the synthesis, modulation and fermentation of gastrointestinal metabolites. Moreover, the microbiome has immunomodulatory properties [57].

Not only antibiotics but also other commonly used drugs in the ICU can interfere with the gut microbiome [58]. Endogeneous bacteria may play a beneficial role in morbidity and mortality of acute illness [59]. However, critical illness leads to disruption of the balance between the intestinal epithelium (increased apoptosis, permeability and mucus alterations all resulting in decreased barrier function) and the microbiome (predominance of pathological bacteria, increased virulence and antibiotic resistance) [57, 60]. This transfer to a critical illness-related ‘disease-promoting microbiome’ or ‘pathobiome’, respectively, may lead to pro-inflammatory downstream events in the intestinal epithelial cells, increased permeability of tight junctions and mucus disintegration, all of which are considered to be associated—both as a cause and consequence—with gastrointestinal injury and multi-organ dysfunction syndrome (MODS) [50, 60]. Emerging, but still preliminary, data in critically ill patients suggest the following: (1) the presence of specific gastrointestinal microbial pathogens at ICU admission is associated with an increased risk for death or all-cause infection, and rectal carriage of common ICU pathogens may predict specific infections [61]; (2) microbiome of critically ill patients undergoes a significant and rapid dysbiosis with loss of diversity, loss of site specificity and a shift toward dominant pathogens as compared to healthy controls [62]; (3) selective decontamination of the digestive tract (SDD)-treated critically ill patients deviate strongly from the gut microbiota of healthy subjects, whereas recolonization of the gut by antibiotic-resistant bacteria may occur upon ICU discharge and cessation of SDD [63]; and (4) lung microbiome is enriched with gut bacteria in acute respiratory distress syndrome [54].

#### Gastrointestinal mucosal integrity

As described (the ‘The role of the gut in multiple organ dysfunction syndrome (MODS)’ section), in animal models, the gut plays a pivotal role to precipitate MODS.

Peterson and Artis have suggested that the intestinal epithelial cells with all the different phenotypes (i.e. enterocytes, goblet cells, Paneth cells, enteroendocrine cells, M cells and intestinal epithelial stem cells) should be recognized as the central regulatory components of barrier function and immune homeostasis [64]. Secretion of epithelial-derived mucins, antimicrobial peptides and IgA create the first line of defence, whilst the tight epithelial lining builds up a physical border. Special cell populations like M cells and dendritic cells act as sensors for pathogens/antigens that activate the local immune response if necessary. Functional or physical loss of this epithelial integrity can lead to further harm [65]. Translocation of (patho)antigens across the epithelial lining may result in the activation of intestinal macrophages and leucocyte recruitment (i.e. intestinal T lymphocytes CD4+ alpha4beta7+ CCR9+) to the intestinal mucosa. The release of cytokines (i.e. tumour necrosis factor, interleukin-1 beta and interleukin-10), reactive oxygen species and nitric oxide may aggravate the intestinal barrier failure and impair gastrointestinal motility by disruption of the tight junctions and smooth muscle contractile elements [66].

#### GI hormones

Molecules secreted from the GI tract may have local effects to modulate motility, mucosal growth and immune function and/or distal hormonal effects on other systems, particularly metabolism [67, 68]. Plasma concentrations of enterohormones have therefore been evaluated as a technique to monitor GI function, but none of them is currently clinically used for this purpose (Table S6). However, the precise relation between GI hormones and GI dysfunction is insufficiently understood.

#### Bile acid signalling

Bile acids have been suggested as a mediator for organ dysfunction [69]. Altered bile acid homeostasis in paediatric patients with intestinal failure has been postulated to contribute to liver dysfunction via increased hepatic bile acid synthesis due to a failing feedback mechanism [70]. Intrahepatic cholestasis of the critically ill is a consequence of alterations of bile acid signalling and transportation at the hepatocellular level. Although the clinical association of cholestasis and inflammation are established, recent studies demonstrated that alterations of hepatic transport and metabolism occur early after ICU admission [69, 70].

In case of malabsorption, the reabsorption of bile acids is reduced and the negative feedback for hepatic bile acid synthesis is inhibited [70]. This mechanism gives a rationale to study bile acid signalling molecules as possible markers of malabsorption and the effects of overproduction of bile acids due to malabsorption (gut-liver axis) on organ dysfunction and outcome.

#### Other pathophysiological mechanisms in GI dysfunction

Pathophysiological mechanisms related to GI dysfunction with potentially impaired outcome in ICU patients are bowel oedema and distension. Gut oedema occurs in the setting of inflammation and capillary leak, fluid resuscitation and increased venous pressure, whereas GI dysmotility may cause bowel distension. Both of them may contribute to (further aggravation of) GI dysfunction.

##### Gut oedema

Scarce existing evidence suggests that gut oedema per se may lead to endotoxaemia, impair intestinal motility and healing of bowel anastomoses, being therefore an important contributor to the outcome.

A study in rodents reported similar activation of signalling pathways in response to intestinal oedema as to mechanical longitudinal bowel distension [71]. Such oedema-induced cell stretch and resulting altered cytoskeleton alterations may explain bowel dysmotility, impaired healing of anastomoses and also endotoxaemia-mediated systemic effects. Bowel oedema can lead to endotoxaemia [72], whereas the effect of oedema on bowel motility may be comparable to the effect of peritonitis [73]. In patients with increased mesenteric venous pressure (caused by right heart failure, mesenteric hypervolaemia or increased intra-abdominal pressure), increased drainage via lymphatics is necessary. With the major increase in such filtration from the capillaries to lymphatics, proteins will be washed out, leading to increased interstitial oncotic pressure and the intestinal interstitial space may become a space with high compliance. The lymphatic flow will then be impaired, further aggravating gut oedema [47].

##### Bowel distension

Bowel distension relates to expansion through increased intra- luminal pressure, manifesting in clinical signs such as bloating or pain [74]. Whilst distension can lead to bowel perforation it can also increase bacterial translocation and stimulate MODS [75].

## Key remaining areas of uncertainty (what we do not know)

Based on a review of the available literature, we identified several areas of uncertainty in GI dysfunction (Table 2). In addition, specific topics with unclear definitions were identified and prioritized for the consensus process. We highlight the following topics:

1. Feeding intolerance: The large variety of definitions is confusing the interpretation of different studies. Consensus definition is needed to identify the clinical importance of feeding intolerance and refine management strategies.
2. Core set of daily monitoring of GI function: Different definitions of different GI symptoms are currently used in studies. Unification of reporting should allow better comparisons of studies regarding the prevalence and clinical relevance of GI symptoms.
3. Core set of outcomes (core outcome set (COS)) in studies addressing GI (dys)function: Unification of reported outcomes would facilitate conduction of meta-analyses.
4. Protocol of abdominal ultrasound to assess GI function (collaboration with radiologists and gastroenterologists): US could possibly supplement the clinical assessment of GI dysfunction, but only if applied in protocolized way.
5. Descriptive definition of non-occlusive mesenteric ischaemia (collaboration with radiologists, gastroenterologists and surgeons): Consensus definition of NOMI is needed to study epidemiology, risks, management and avoidance of this severe syndrome which may sometimes be related to therapy provided to critically ill patients.
6. Reference methods to be used to measure gastric emptying, absorption of nutrients and barrier dysfunction in studies in critically ill patients

**Table 2:** Remaining areas of uncertainty in gastrointestinal dysfunction of critically ill patients (what we do not know) We describe these areas as high-level open-ended questions, to stimulate further research formulating specific questions. We highlight in bold the subjects that were chosen by the panel for needing consensus process and prioritized as the next tasks for the Working Group on GI function of the Section of MEN of ESICM

Subtopic		Remaining areas of uncertainties
**Monitoring of GI dysfunction**		
i.	Clinical assessment	How are GI symptoms* associated with GI function? **How to monitor GI function daily at bedside = what is the core set of daily monitoring of GI function?** Which symptoms and when should trigger more complex diagnostics? Whether a combination of clinical assessment with specific diagnostics/monitoring methods could allow developing a reliable scoring system for GI dysfunction in critically ill? **How to define feeding intolerance?** **What is the reference method to be used to measure gastric emptying in studies in critically ill patients?** Can measurement of gastric residual volumes identify delayed gastric emptying? Can monitoring of gastric residual volumes help in avoiding complications in patients with feeding intolerance?
ii.	Imaging	**What is the best abdominal ultrasound protocol for GI dysfunction?** Needs collaboration with radiologists and gastroenterologists. Whether US image quality (which may be affected by air in the GI tract) can be further improved? What imaging technique is associated with high inter-operator and over time reproducibility? How to quantify bowel oedema?
iii.	Laboratory (including biomarkers)	Which biomarker(s) could be used as a marker of GI dysfunction? Which biomarker(s) could be used in decision-making regarding enteral nutrition? Which biomarkers could be used in decision-making regarding non-occlusive mesenteric ischaemia?
iv.	Absorption of nutrients	**What is the reference method to be used to measure absorption of nutrients in studies in critically ill patients?** What are the possible novel methods to measure absorption of nutrients at bedside?
v.	Barrier function	**What is the reference method to be used to measure barrier dysfunction in studies in critically ill patients?** How to differentiate between pathological and physiological GI mucosal permeability? What are the possible novel methods to measure/detect the presence of barrier dysfunction in studies in critically ill patients? What are the possible novel methods to estimate barrier dysfunction at bedside? What are the possible biomarkers that can rapidly detect barrier dysfunction caused by mesenteric ischaemia? (How) does the microbiome influence the gut-derived immunity?
vi.	Others	Which GI symptoms could identify the cohort of patients who would benefit from monitoring of intra-abdominal pressure? What are the other possible novel strategies to monitor/assist in monitoring of GI function?
**GI dysfunction: reporting and outcome**		
		What is the natural course of GI dysfunction in survivors of critical illness? Which are the differences between primary and secondary GI injury? **What is the core set of daily monitoring of GI function?** **What are the ‘core outcomes’ for GI dysfunction in critically ill patients?** How to define non-occlusive mesenteric ischaemia (collaboration with radiologists, gastroenterologists and surgeons)?
**Management of GI dysfunction**		
vii.	Prokinetics	Which are the indications to use prokinetics? Which novel prokinetic agents with less side effects could be used in clinical practice?
viii.	Laxatives	Which are the indications for laxative agents? Which laxatives, when and in which dosage should be applied?
ix.	Post-pyloric feeding	Which are the indications to use post-pyloric feeding? How does post-pyloric feeding compare to gastric feeding with prokinetic drugs on patient-centred outcomes? Are there differences between duodenal and jejunal feeding?
x.	Others	Which other management options could be used to prevent and/or improve GI dysfunction? Could specific fluid resuscitation strategy reduce the prevalence and severity of GI dysfunction? Could achievement of high-normal levels of electrolytes (potassium and magnesium) improve GI motility? Could early mobilization improve GI function in ICU patients? Which sedation strategy associated with less GI dysfunction?
**xi.**	GI function and nutrition	What is the optimal timing and duration for both trophic EN to ‘feed the mucosa and microbiome’ and ‘full feeding’ to match estimated energy expenditure? Does early EN benefits or harms GI function (e.g. absorptive capacity and barrier function of the gut) when compared to fasting or PN? Should intolerance of EN be accepted as a protective adaptive response to critical illness or treated to increase nutrient delivery? How and when should EN be initiated and/or increased to best maintain and/or improve GI function?
**Pathophysiological mechanisms in GI dysfunction relevant to the outcome**		
xii.	The role of the gut in multiple organ failure	**What is the best definition and estimates of the prevalence of NOMI?** How to achieve earlier identification of NOMI (e.g. with additional biomarkers or other tests of gut (hypo)perfusion)? What are the feeding strategies to reduce the rate of NOMI (e.g. early fasting vs early trophic EN)? What interventions are effective in conservative management of NOMI? When and how does GI injury cause multiple organ failure and vice versa?
xiii.	Microbiome	What mechanisms underlie changes in microbiota density, genus abundance, community structure and function during critical illness? Which role do microbiota-modulated metabolite function and inter-organ cross-talk play in critically ill patients? What are the causes and consequences of dysbiosis on gastrointestinal injury and organ dysfunction? Are there valid biomarkers for microbiome-related GI-dysfunction? Can personalized microbiome type- and function-directed interventions improve organ dysfunction and ICU-related outcomes? What are the best sampling methods for the specimen (e.g. stool vs rectal swabs)?
xiv.	Bacterial translocation /mucosal integrity	Which are the mechanistic approaches to protect mucosal integrity? Whether and how can/should the immune response on the loss of mucosal integrity be modulated?
xv.	GI hormones	Which GI hormones are inadequately secreted in critical illness? Quantify response to ‘normal’ endogenous or physiological concentrations? What is the effect of restoring secretion of hormones/levels of hormones to ‘normal’ endogenous or physiological concentrations?
xvi.	Bile acid signalling	Could plasma concentrations of bile acid signalling molecules be used as a marker of malabsorption?
xvii.	Others	How does development of bowel oedema impact GI motility and vice versa? What are the differences between the direct GI injury resulting in oedema vs GI injury due to generalized oedema?

*GI symptoms include vomiting/regurgitation, abdominal distension, GI bleeding, diarrhoea and lower GI paralysis [3]. Expanded (if performed/possible to assess): nausea, abdominal pain, absence of bowel sounds, large GRV (> 500 mL/6 h), bowel dilatation (radiological) and bowel wall thickening/bowel oedema (radiological)

It should be recognized that in the light of the current poor evidence, these consensus definitions will likely need to be adapted in the future if new evidence emerges. At the same time, they are crucial to produce and systematize this new evidence.

## Research agenda (what we need to know)

The panel formulated 32 study proposals (Additional file 3, Table S4) that underwent voting. The following studies (Table 3) were selected via voting to have the priority using the methodology outlined in Additional file 1:

**Table 3:** Top ten study proposals for future research on GI dysfunction (what we need to know)

Final rank and acronym	Research questions/aims	Study design	Study population	Indicator/Intervention	Suggested outcome variables	Points
1. Diarrhoea prevention	Does routine use of fibre-enriched EN reduce diarrhoea?	RCT	Critically ill patients with EN with a standard protocol	Fibre-enriched vs non-fibre EN	Bristol stool chart GI symptoms*	400
2. Opioid antagonists for bowel paralysis	Do opioid antagonists reduce time to defaecation and GI symptoms? Potential substudy: study the impact on intestinal absorption	Multicentre RCT	Adult ICU patients with opioid requirement above a minimal dosage	Methylnaltrexone (or other opioid antagonists) vs placebo	Time to the first defaecation COS^#^ GI symptoms* Infections Substudy: absorption	373
3. Diarrhoea management	Does reduction or discontinuation of EN reduce diarrhoea?	RCT (3-armed study)	Patients with severe diarrhoea during EN Severe diarrhoea = requiring interventions (fluids and electrolytes).	1. Continuation of EN 2. Reduction of EN (50%) 3. Trophic EN + supplemental PN (after 3–7 days)	Bristol stool chart GI symptoms* LOS infections	343
4. Prophylaxis vs treatment of upper GI intolerance	Is the prophylactic use of prokinetics superior to therapeutic use?	Multicentre RCT	Adult ICU patients at high risk for gastroparesis (e.g. patients with high doses of opioids, post-GI surgery)	Two study arms, the same drugs (e.g. erythromycin, metoclopramide, alizapride) and dosages, different timing (routine administration or only in confirmed gastroparesis)	COS^#^ safety outcomes Long-term outcomes (prolonged QT, extrapyramidal side effects, colonization with multi-resistant microbes)	335
5. Prophylaxis vs treatment of lower GI intolerance	Does the prophylactic use of motility agents (prokinetics and laxative drugs) reduce time to defaecation and improve feeding tolerance and GI dysfunction based on AGI grading?	Multicentre RCT	Adult consecutive ICU patients with an expected ICU stay of ≥ 3 days?	Two study arms, the same drugs (e.g. macrogol, laxatives) and dosages, different timing (routine administration or only in confirmed constipation/bowel paralysis)	Time to defaecation AGI dynamics COS (clinical outcomes) Infections Diarrhoea Mesenteric ischaemia	323
6. IAH-GI + NOMI-AGI	1. Does protocolised monitoring of IAP and management of IAH improve outcome? 2. Is increased IAP associated with GI dysmotility?	1. RCT 2. Observational substudy	MV patients at risk of IAH	Intervention: monitoring and management of IAP based on the protocol (bundle of preventive measures) Control: standard care US in the intervention group	Mesenteric ischaemia Incidence of infections/sepsis Mortality 90 days, LOS, GI symptoms* Obervational substudy: correlation between IAP and GI motility as assessed by US	312
7. Indication of post-pyloric feeding	Is post-pyloric feeding superior to PN in case of gastroparesis?	Multicentre RCT	Adult ICU patients with gastroparesis (e.g. GRV > 500 mL with prokinetics)	Post-pyloric feeding vs PN	Infections Mortality GI complications (including non-occlusive bowel ischaemia), meeting nutritional target	290
8. GI and IAP	Which GI symptoms* should trigger IAP measurements? Which IAP values should trigger specific monitoring of GI?	Post hoc analysis of combined databases (prospective observational)	Patients in performed studies	Identification and merging of existing databases	Association of GI symptoms with IAH, mesenteric ischaemia and mortality	274
9. AGI prospective	Does AGI score (AGI I–IV) predict the outcome?	Prospective observational	Consecutive ICU patients being mechanically ventilated for non-elective reason (planned MV after elective surgery excluded)	AGI score documented daily Decisions for diagnostics or treatment taken based on daily assessed GI symptoms* documented daily Preferably similar feeding protocol in all centres	GI symptoms* Pneumonia COS^#^ ICU outcome 90-day outcome Long-term patient-centred outcome NOBN GI anastomosis leakage (if relevant)	272
10. PPI and dysbiosis	Does use of PPI vs no PPI alters the intestinal microbiome?	RCT	Mech. ventilated ICU patients in need of EN and without an absolute indication for PPI	PPI vs no PPI	Faecal microbiome pattern Incidence of *Clostridium difficile* colitis	249

49/55 completed the first voting

43/49 completed the second voting

Points gained from two main general questions in voting 2 (see Supplement 1 for details) are presented

*AGI* acute gastrointestinal injury, *COS* core outcome set, *EN* enteral nutrition, *GI* gastrointestinal, *GRV* gastric residual volume, *IAH* intra-abdominal hypertension, *IAP* intra-abdominal pressure, *ICU* intensive care unit, *LOS* length of stay, *MV* mechanical ventilation, *NOBN* non-occlusive bowel necrosis, *NOMI* non-occlusive mesenteric ischaemia, *PN* parenteral nutrition, *PPI* proton pump inhibitors, *RCT* randomized controlled trial, *US* ultrasound

*GI symptoms include vomiting/regurgitation, abdominal distension, GI bleeding, diarrhoea and lower GI paralysis [3]. Expanded (if performed/possible to assess): nausea, abdominal pain, absence of bowel sounds, large GRV (> 500 mL/6 h), bowel dilatation (radiological) and bowel wall thickening/bowel oedema (radiological)

^#^Core outcome set (COS) to be identified in the consensus process

*Studies on prevention and management of diarrhoea* (ranks 1 and 3, respectively) as well as *upper and lower GI feeding intolerance* (ranks 4 and 5) were ranked high, stressing the perceived importance of these very practical problems at the bedside and the feasibility to study these issues. Future research on diarrhoea and feeding intolerance is definitely not limited to the proposed study ideas, offering a much broader field. Studies on *opioid antagonists* (rank 2) and *indications for post-pyloric feeding* (rank 7) also refer to the management of feeding intolerance, whereas testing of *AGI grading* (rank 9) as a clinical bedside tool includes monitoring of feeding intolerance. The role of *intra-abdominal hypertension in development of NOMI* (rank 6), *in development and in monitoring of GI dysfunction* (rank 8) and *the effect of proton pump inhibitors on microbiome* of critically ill (rank 10) were prioritized.

We want to emphasize the importance of all the study proposals included in Table S4. The above-presented ranking of the study projects also considered the feasibility of the projects. Feasibility of several proposals could not be evaluated as ‘high’ due to concerns about definitions, a priori necessary development in methodology or very high costs, even though the answers to raised research questions would be most warranted. At the same time, feasible studies gained higher ranking, explaining the prominent position of rather straight-forward and practical studies in the final list. However, e.g., studies on diarrhoea also need to be seen in the context of later more sophisticated studies addressing feeding intolerance and malabsorption. The lack of uniformity/consensus in definitions regarding GI function was recognized to be a major limiting impact on future research. Therefore, initiation of consensus processes on topics listed in the previous section was formulated as the next task of the WG.

## Discussion of strengths and limitations

The main strength of this scoping review is the unified effort of a large group of experts to systematize available information and establish a framework to improve research in this field. The main issues hindering any research on GI dysfunction comprise the absence of uniform definitions and the lack of gold-standard methods for measuring/monitoring GI function.

Limitations of this work are that searches were limited to the English language, the interpretation of current evidence represents a consensus summary, the list of study proposals is not exhaustive and both proposals and current evidence may be influenced by individual academic or industry bias despite the consensus approach. In addition, voting methodology emphasized feasibility, which may rapidly change with advancement in research methodology. Finally, all voting members were clinician/researcher, and patients may have prioritized differently.

## Conclusions

Despite the high morbidity, causes and consequences of gastrointestinal dysfunction in critically ill patients are insufficiently understood. To improve the consistency of future studies, we propose the areas for consensus process and outline future study projects. Studies on the monitoring, prevention and management of diarrhoea and feeding intolerance received the highest ranking on the research agenda.

## Supplementary information

**Additional file 1: **Methods and conflicts of interest. This file presents 1) detailed information on methods; 2) authors’ contributions and 3) disclosure of conflicts of interest. **Table S1.** in this file presents academic conflicts of interest.

**Additional file 2:** Summary of evidence. This file presents results of systematic reviews, summary of evidence and references of all papers selected for full text assessment. **Table S2.** in this file presents search strategies and results of screening of titles/abstracts for each research topic. **Table S3.** in this file presents summary of evidence for each research topics, referring to selected papers supporting this summary. All papers selected for full text assessment are listed, papers supporting summary of evidence (presented in Table 3 in the main manuscript and Table S3 in this file) are marked with asterisk.

**Additional file 3:** All study proposals. This file includes **Table S4.** presenting all study proposals.

**Additional file 4:** Monitoring and motility. This file includes summary on monitoring of GI function, biomarkers of GI dysfunction with description of specific aspects and pifalls in laboratory measurements, and summary of drugs influencing GI motility. **Table S5.** presents clinical assessment, imaging and specific tools used to assess motility and perfusion. **Table S6.** presents possible laboratory biomarkers of GI dysfunction. **Table S7.** presents summary on GI motility drugs based on systematic review.

**Additional file 5.** PRISMA checklist. This file includes PRISMA (Preferred Reporting Items for Systematic reviews and Meta-Analyses) extension for Scoping Reviews (PRISMA-ScR) checklist.

**Additional file 6.** PRISMA Flow diagrams. This file presents PRISMA (Preferred Reporting Items for Systematic reviews and Meta-Analyses) Flow diagrams for each of 16 systematic reviews separately.

## Data Availability

All papers included in the full-text assessment are listed in Additional file 2.

## Abbreviations

ARDS: Acute respiratory distress syndrome
CT: Computer tomography
CI: Confidence interval
EN: Enteral nutrition
ERAS: Enhanced recovery after surgery
ESICM: European Society of Intensive Care Medicine
GALT: Gut-associated lymphoid tissue
GRV: Gastric residual volume
GI: Gastrointestinal
GLP-1: Glucagon-like peptide-1
GIP: Glucose-dependent insulinotropic polypeptide
ICU: Intensive care unit
IAH: Intra-abdominal hypertension
IAP: Intra-abdominal pressure
MODS: Multi-organ dysfunction syndrome
NOMI: Non-occlusive mesenteric ischaemia
PN: Parenteral nutrition
PRISMA: Preferred Reporting Items for Systematic reviews and Meta-Analyses
RCT: Randomized controlled trial
RT-PCR: Reverse transcription polymerase chain reaction
MEN Section: Section of Metabolism, Endocrinology and Nutrition
SDD: Selective decontamination of the digestive tract
US: Ultrasound

## References

[1] Reintam Blaser A, Poeze M, Malbrain ML, Björck M, Oudemans-van Straaten HM, Starkopf J, Gastro-Intestinal Failure Trial Group (2013). Gastrointestinal symptoms during the first week of intensive care are associated with poor outcome: a prospective multicentre study. Intensive Care Med.

[2] Moonen PJ, Reintam Blaser A, Starkopf J, Oudemans-van Straaten HM, Van der Mullen J, Vermeulen G, Malbrain MLNG (2018). The black box revelation: monitoring gastrointestinal function. Anaesthesiol Intensive Ther.

[3] Reintam Blaser A, Malbrain ML, Starkopf J, Fruhwald S, Jakob SM, De Waele J (2012). Gastrointestinal function in intensive care patients: terminology, definitions and management. Recommendations of the ESICM Working Group on Abdominal Problems. Intensive Care Med.

[4] Asrani VM, Brown A, Huang W, Bissett I, Windsor JA. Gastrointestinal dysfunction in critical illness: a review of scoring tools. JPEN J Parenter Enteral Nutr. 2019 Jul 26. 10.1002/jpen.1679 [Epub ahead of print]. 10.1002/jpen.1679 31350771

[5] Lefering R, Goris RJ, van Nieuwenhoven EJ, Neugebauer E (2002). Revision of the multiple organ failure score. Langenbeck’s Arch Surg.

[6] Mayr VD, Dünser MW, Greil V, Jochberger S, Luckner G, Ulmer H (2006). Causes of death and determinants of outcome in critically ill patients. Crit Care.

[7] D’Hondt A, Haentjens L, Brassart N, Flamme F, Preiser JC (2017). Uncontrolled bleeding of the gastrointestinal tract. Curr Opin Crit Care.

[8] Reignier J, Mercier E, Le Gouge A, Boulain T, Desachy A, Bellec F (2013). Effect of not monitoring residual gastric volume on risk of ventilator-associated pneumonia in adults receiving mechanical ventilation and early enteral feeding: a randomized controlled trial. JAMA..

[9] Wierdsma NJ, Peters JH, Weijs PJ, Keur MB, Girbes AR, van Bodegraven AA, Beishuizen A (2011). Malabsorption and nutritional balance in the ICU: fecal weight as a biomarker: a prospective observational pilot study. Crit Care.

[10] Hay T, Bellomo R, Rechnitzer T (2019). Constipation, diarrhea, and prophylactic laxative bowel regimens in the critically ill: a systematic review and meta-analysis. J Crit Care.

[11] Reignier J, Boisramé-Helms J, Brisard L, Lascarrou JB, Ait Hssain A, Anguel N (2018). Enteral versus parenteral early nutrition in ventilated adults with shock: a randomised, controlled, multicentre, open-label, parallel-group study (NUTRIREA-2). Lancet..

[12] Hamada SR, Garcon P, Ronot M, Kerever S, Paugam-Burtz C, Mantz J (2014). Ultrasound assessment of gastric volume in critically ill patients. Intensive Care Med.

[13] Perez-Calatayud AA, Carrillo-Esper R, Anica-Malagon ED, Briones-Garduño JC, Arch-Tirado E, Wise R, Malbrain MLNG (2018). Point-of-care gastrointestinal and urinary tract sonography in daily evaluation of gastrointestinal dysfunction in critically ill patients (GUTS Protocol). Anaesthesiol Intensive Ther..

[14] Treskes N, Persoon AM, van Zanten ARH (2017). Diagnostic accuracy of novel serological biomarkers to detect acute mesenteric ischemia: a systematic review and meta-analysis. Intern Emerg Med.

[15] Crenn P, Messing B, Cynober L (2008). Citrulline as a biomarker of intestinal failure due to enterocyte mass reduction. Clin Nutr.

[16] Piton G, Manzon C, Cypriani B, Carbonnel F, Capellier G (2011). Acute intestinal failure in critically ill patients: is plasma citrulline the right marker?. Intensive Care Med.

[17] Reintam Blaser A, Padar M, Tang J, Dutton J, Forbes A (2019). Citrulline and intestinal fatty acid-binding protein as biomarkers for gastrointestinal dysfunction in the critically ill. Anaesthesiol Intensive Ther.

[18] Deane AM, Summers MJ, Zaknic AV, Chapman MJ, Di Bartolomeo AE, Bellon M (2011). Glucose absorption and small intestinal transit in critical illness. Crit Care Med.

[19] Ali Abdelhamid Y, Cousins CE, Sim JA, Bellon MS, Nguyen NQ, Horowitz M (2015). Effect of critical illness on triglyceride absorption. JPEN J Parenter Enteral Nutr.

[20] Liebau F, Wernerman J, van Loon LJ, Rooyackers O (2015). Effect of initiating enteral protein feeding on whole-body protein turnover in critically ill patients. Am J Clin Nutr.

[21] Berger MM, Werner D, Revelly JP, Cayeux MC, Tappy L, Bachmann C, Chiolero RL (2003). Serum paracetamol concentration: an alternative to X-rays to determine feeding tube location in the critically ill. JPEN J Parenter Enteral Nutr.

[22] Liew VY, Chapman MJ, Nguyen NQ, Cousins CE, Plummer MP, Chapple LA (2016). A prospective observational study of the effect of critical illness on ultrastructural and microscopic morphology of duodenal mucosa. Crit Care Resusc.

[23] Camilleri M, Madsen K, Spiller R, Greenwood-Van Meerveld B, Verne GN (2012). Intestinal barrier function in health and gastrointestinal disease. Neurogastroenterol Motil.

[24] Oudemans-van Straaten HM, van der Voort PJ, Hoek FJ, Bosman RJ, van der Spoel JI, Zandstra DF (2002). Pitfalls in gastrointestinal permeability measurement in ICU patients with multiple organ failure using differential sugar absorption. Intensive Care Med.

[25] Kramski M, Gaeguta AJ, Lichtfuss GF, Rajasuriar R, Crowe SM, French MA (2011). Novel sensitive real-time PCR for quantification of bacterial 16S rRNA genes in plasma of HIV-infected patients as a marker for microbial translocation. J Clin Microbiol.

[26] Miller SI, Ernst RK, Bader MW (2005). LPS, TLR4 and infectious disease diversity. Nat Rev Microbiol.

[27] Kirkpatrick AW, Roberts DJ, De Waele J, Jaeschke R, Malbrain ML, De Keulenaer B (2013). Intra-abdominal hypertension and the abdominal compartment syndrome: updated consensus definitions and clinical practice guidelines from the World Society of the Abdominal Compartment Syndrome. Intensive Care Med.

[28] Reintam Blaser A, Parm P, Kitus R, Starkopf J. Intra-abdominal hypertension and gastrointestinal symptoms in mechanically ventilated patients. Crit Care Res Pract. 2011;982507. 10.1155/2011/982507. 10.1155/2011/982507 PMC3087429 21547094

[29] Blackwood B, Marshall J, Rose L (2015). Progress on core outcome sets for critical care research. Curr Opin Crit Care.

[30] Fruhwald S, Holzer P, Webb A, Angus D, Finfer S, Gattinoni L, Singer M (2016). Gastrointestinal motility drugs in critical illness. Oxford textbook of critical care.

[31] Nguyen NQ, Chapman M, Fraser RJ, Bryant LK, Burgstad C, Holloway RH (2007). Prokinetic therapy for feed intolerance in critical illness: one drug or two?. Crit Care Med.

[32] Lewis K, Alqahtani Z, McIntyre L, Almenawer S, Alshamsi F, Rhodes A (2016). The efficacy and safety of prokinetic agents in critically ill patients receiving enteral nutrition: a systematic review and meta-analysis of randomized trials. Crit Care.

[33] Singer P, Reintam Blaser A, Berger MM, Alhazzani W, Calder PC, Casaer MP (2019). ESPEN guideline on clinical nutrition in the intensive care unit. Clin Nutr.

[34] Deane AM, Lamontagne F, Dukes GE, Neil D, Vasist L, Barton ME (2018). Nutrition adequacy therapeutic enhancement in the critically ill: a randomized double-blind, placebo-controlled trial of the motilin receptor agonist camicinal (GSK962040): the NUTRIATE Study. JPEN J Parenter Enteral Nutr.

[35] van der Spoel JI, Oudemans-van Straaten HM, Stoutenbeek CP, Bosman RJ, Zandstra DF (2001). Neostigmine resolves critical illness-related colonic ileus in intensive care patients with multiple organ failure - a prospective, double-blind, placebo-controlled trial. Intensive Care Med.

[36] Valle RG, Godoy FL (2014). Neostigmine for acute colonic pseudo-obstruction: a meta-analysis. Ann Med Surg (Lond).

[37] Oczkowski SJW, Duan EH, Groen A, Warren D, Cook DJ (2017). The use of bowel protocols in critically ill adult patients: a systematic review and meta-analysis. Crit Care Med.

[38] Taylor BE, McClave SA, Martindale RG, Warren MM, Johnson DR, Braunschweig C (2016). Guidelines for the provision and assessment of nutrition support therapy in the adult critically ill patient: Society of Critical Care Medicine (SCCM) and American Society for Parenteral and Enteral Nutrition (A.S.P.E.N.). Crit Care Med.

[39] Deane AM, Dhaliwal R, Day AG, Ridley EJ, Davies AR, Heyland DK (2013). Comparisons beween intragastric and small intestinal delivery of enteral nutrition in the critically ill: a systematic review and meta-analysis. Crit Care.

[40] Lobo DN, Bostock KA, Neal KR, Perkins AC, Rowlands BJ, Allison SP (2002). Effect of salt and water balance on recovery of gastrointestinal function after elective colonic resection: a randomised controlled trial. Lancet..

[41] Myles PS, Bellomo R, Corcoran T, Forbes A, Peyton P, Story D (2018). Restrictive versus liberal fluid therapy for major abdominal surgery. N Engl J Med.

[42] Kar P, Jones KL, Plummer MP, Ali Abdelhamid Y, Giersch EJ, Summers MJ (2017). Antecedent hypoglycemia does not attenuate the acceleration of gastric emptying by hypoglycemia. J Clin Endocrinol Metab.

[43] Brigode WM, Jones C, Vazquez DE, Evans DC (2015). Scrutinizing the evidence linking hypokalemia and ileus: a commentary on fact and dogma. Int J Acad Med.

[44] Barletta JF, Asgeirsson T, Senagore AJ (2011). Influence of intravenous opioid dose on postoperative ileus. Ann Pharmacother.

[45] Rubinoff MJ, Piccione PR, Holt PR (1989). Clonidine prolongs human small intestine transit time: use of the lactulose-breath hydrogen test. Am J Gastroenterol.

[46] Wallström A, Frisman GH (2014). Facilitating early recovery of bowel motility after colorectal surgery: a systematic review. J Clin Nurs.

[47] Malbrain ML, Viaene D, Kortgen A, De Laet I, Dits H, Van Regenmortel N (2012). Relationship between intra-abdominal pressure and indocyanine green plasma disappearance rate: hepatic perfusion may be impaired in critically ill patients with intra-abdominal hypertension. Ann Intensive Care.

[48] Verbrugge FH, Dupont M, Steels P, Grieten L, Malbrain M, Tang WH, Mullens W (2013). Abdominal contributions to cardiorenal dysfunction in congestive heart failure. J Am Coll Cardiol.

[49] Reintam Blaser A, Regli A, De Keulenaer B, Kimball EJ, Starkopf L, Davis WA (2019). Incidence, risk factors, and outcomes of intra-abdominal hypertension in critically ill patients - a prospective multicenter study (IROI Study). Crit Care Med.

[50] Nguyen NQ, Besanko LK, Burgstad C, Bellon M, Holloway RH, Chapman M (2012). Delayed enteral feeding impairs intestinal carbohydrate absorption in critically ill patients. Crit Care Med.

[51] Ralls MW, Demehri FR, Feng Y, Woods Ignatoski KM, Teitelbaum DH (2015). Enteral nutrient deprivation in patients leads to a loss of intestinal epithelial barrier function. Surgery..

[52] Reintam Blaser A, Starkopf J, Alhazzani W, Berger MM, Casaer MP, Deane AM (2017). Early enteral nutrition in critically ill patients: ESICM clinical practice guidelines. Intensive Care Med.

[53] Chen G, Huang B, Fu S, Li B, Ran X, He D (2018). G protein-coupled receptor 109A and host microbiota modulate intestinal epithelial integrity during Sepsis. Front Immunol.

[54] Meng M, Klingensmith NJ, Coopersmith CM (2017). New insights into the gut as the driver of critical illness and organ failure. Curr Opin Crit Care.

[55] Dickson RP, Singer BH, Newstead MW, Falkowski NR, Erb-Downward JR, Standiford TJ, Huffnagle GB (2016). Enrichment of the lung microbiome with gut bacteria in sepsis and the acute respiratory distress syndrome. Nat Microbiol.

[56] Deitch EA, Adams C, Lu Q, Xu DZ (2001). A time course study of the protective effect of mesenteric lymph duct ligation on hemorrhagic shock-induced pulmonary injury and the toxic effects of lymph from shocked rats on endothelial cell monolayer permeability. Surgery..

[57] Wolff NS, Hugenholtz F, Wiersinga WJ (2018). The emerging role of the microbiota in the ICU. Crit Care.

[58] Le Bastard Q, Al-Ghalith GA, Grégoire M, Chapelet G, Javaudin F, Dailly E (2018). Systematic review: human gut dysbiosis induced by non-antibiotic prescription medications. Aliment Pharmacol Ther.

[59] Fox AC, McConnell KW, Yoseph BP, Breed E, Liang Z, Clark AT (2012). The endogenous bacteria alter gut epithelial apoptosis and decrease mortality following Pseudomonas aeruginosa pneumonia. Shock..

[60] Alverdy JC, Krezalek MA (2017). Collapse of the microbiome, emergence of the pathobiome, and the immunopathology of sepsis. Crit Care Med.

[61] Freedberg DE, Zhou MJ, Cohen ME, Annavajhala MK, Khan S, Moscoso DI (2018). Pathogen colonization of the gastrointestinal microbiome at intensive care unit admission and risk for subsequent death or infection. Intensive Care Med.

[62] Yeh A, Rogers MB, Firek B, Neal MD, Zuckerbraun BS, Morowitz MJ (2016). Dysbiosis across multiple body sites in critically ill adult surgical patients. Shock..

[63] Buelow E, Bello González TDJ, Fuentes S, de Steenhuijsen Piters WAA, Lahti L, Bayjanov JR (2017). Comparative gut microbiota and resistome profiling of intensive care patients receiving selective digestive tract decontamination and healthy subjects. Microbiome..

[64] Peterson LW, Artis D (2014). Intestinal epithelial cells: regulators of barrier function and immune homeostasis. Nat Rev Immunol.

[65] Hotchkiss RS, Swanson PE, Freeman BD, Tinsley KW, Cobb JP, Matuschak GM (1999). Apoptotic cell death in patients with sepsis, shock, and multiple organ dysfunction. Crit Care Med.

[66] Greis C, Rasuly Z, Janosi RA, Kordelas L, Beelen DW, Liebregts T (2017). Intestinal T lymphocyte homing is associated with gastric emptying and epithelial barrier function in critically ill: a prospective observational study. Crit Care.

[67] Santacruz CA, Quintairos A, Righy C, Crippa IA, Couto L, Imbault V (2017). Is there a role for enterohormones in the gastroparesis of critically ill patients?. Crit Care Med.

[68] Plummer MP, Kar P, Cousins CE, Hausken T, Lange K, Chapman MJ (2016). Critical illness is associated with impaired gallbladder emptying as assessed by 3D ultrasound. Crit Care Med.

[69] Horvatits T, Drolz A, Rutter K, Roedl K, Langouche L, Van den Berghe G (2017). Circulating bile acids predict outcome in critically ill patients. Ann Intensive Care.

[70] Xiao YT, Cao Y, Zhou KJ, Lu LN, Cai W (2016). Altered systemic bile acid homeostasis contributes to liver disease in pediatric patients with intestinal failure. Sci Rep.

[71] Shah SK, Fogle LN, Aroom KR, Gill BS, Moore-Olufemi SD, Jimenez F (2010). Hydrostatic intestinal edema induced signaling pathways: potential role of mechanical forces. Surgery..

[72] Turnage RH, Guice KS, Oldham KT (1994). Endotoxemia and remote organ injury following intestinal reperfusion. J Surg Res.

[73] Gorrasi J, Jakob SM, Tovar L, Balsiger B, Brandt S, Bruegger LE (2018). Perioperative fluid accumulation impairs intestinal contractility to a similar extent as peritonitis and endotoxemia. Shock..

[74] Reintam Blaser A, Starkopf J, Moonen PJ, Malbrain MLNG, Oudemans-van Straaten HM (2018). Perioperative gastrointestinal problems in the ICU. Anaesthesiol Intensive Ther..

[75] Lee TC, Huang YC, Lu YZ, Yeh YC, Yu LC (2018). Hypoxia-induced intestinal barrier changes in balloon-assisted enteroscopy. J Physiol.

