# Supplementary material for: Gastrointestinal dysfunction in the critically ill: a systematic scoping review and research agenda proposed by the Section of Metabolism, Endocrinology and Nutrition of the European Society of Intensive Care Medicine
Source: Crit Care. 2020 May 15;24:224. doi: 10.1186/s13054-020-02889-4 (PMC7226709; doi:10.1186/s13054-020-02889-4)
Supplement: Supplementary file 1 — Additional file 1: Methods and conflicts of interest. This file presents 1) detailed information on methods; 2) authors’ contributions and 3) disclosure of conflicts of interest. Table S1. in this file presents academic conflicts of interest. [file 13054_2020_2889_MOESM1_ESM.docx]

**Additional file 1**

**Methods and conflicts of interest**

Contents

1. Methods……………………………………………………1-5
2. Authors’ contributions …………………………………….6-7
3. Conflicts of interest ………………………………………..8-9
4. Table S1. Academic conflicts of interest…………………..10

**Methods**

*Group members*

This task was formulated and completed by the Working Group (WG) on Gastrointestinal Function within the Metabolism, Endocrinology and Nutrition (MEN) Section of the European Society of Intensive Care Medicine (ESICM). A methodologist (CB) was engaged from outside the Working Group. All members of the WG were invited to participate in drafting the manuscript, propose research topics and vote on priorities of study proposals. All MEN section members, who expressed interest in voting (according to the results of a preceding web-based survey to identify the level of interest of the members in activities to the MEN section), were invited to participate. No clinical experts were invited from outside of the group.

Two group meetings were held during Annual Congresses of ESICM 2017 and 2018, four web-meetings in 2017-2019, and the work in-between was organized via email correspondence.

*Management of conflict of interest*

The study was not funded.

There was no industry input. No industry representatives were present at any of the meetings. Possible issues of conflict of interest were discussed, none of them was considered relevant. All group members had the possibility to propose new research projects and suggest on already proposed research topics. Each group member had one vote during selection of study proposals. Voting on the priorities took place via a web-based questionnaire and the individual choices were not visible to the other group members.

Individual conflicts of interest (COI) are reported at the end of this document.

The group agreed that COIs would not create major bias in the voting process because the vast majority of research proposals did not include one certain methodology/drug etc. that could pertain to specific non-/financial or scientific COIs. Thus, no rules or restrictions were considered necessary for the voting process.

*Identification of sub-topics*

Based on consensus reached during the Working Group meetings and e-mail discussions, we predefined research categories as:

1) Monitoring of GI function in critical illness;

2) Reporting and outcome of GI dysfunction in critically ill patients;

3) Management of GI dysfunction in critically ill patients;

4) GI function and nutrition in critically ill patients;

5) Pathophysiological mechanisms in GI dysfunction relevant to outcome in critically ill patients.

After identification of the five main categories, e-mail discussions between the co-authors were continued to refine sub-topics under each of these categories. Finally it was agreed to perform searches on 17 sub-topics as presented in the manuscript and in **Table 1, and Table S2 in Additional file 2**, whereas not performing specific search in the category ‘Reporting and outcome’. Each sub-topic was then assigned to two group members for literature assessment, summary of evidence, drafting corresponding parts of the manuscript to summarize existing evidence and identifying areas and rationale for future research.

*Systematic reviews*

The aim of our study was to perform a scoping review using explicit systematic methods to determine the scope and coverage of the body of literature on the topic gastrointestinal function in critically ill patients and to give a clear indication of the available volume of literature. This systematic scoping review was performed according to the Cochrane handbook (<https://handbook-5-1.cochrane.org>), and its reporting is in line with the PRISMA statement (<https://www.ncbi.nlm.nih.gov/pubmed/19622552>). PRISMA Checklist is presented in **Additional file 5** and PRISMA flow charts for each systematic review in **Additional file 6.**

Following group agreement of the questions and outcomes, we formulated a search strategy for each question (see **Table S2 in Additional file 2**). Systematic searches of electronic databases were performed by an independent methodologist (CB), using PubMed, CENTRAL and the Cochrane Database of Systematic Reviews from inception to end of November 2019.

As the aim of the project was to identify research priorities, we did not perform meta-analyses. The study population of main interest was the adult critically ill patient. Due to the scarce evidence in assessment and management of GI (dys) function in the critically ill, we decided to report all papers possibly relevant for future research not necessarily limited to clinical studies in the adult critically ill. Therefore, no clear eligibility criteria were defined for selection of relevant papers.

We included studies focusing on critical illness, and depending on the respective topic we limited the study type to clinical trials, observational studies, case series, experimental studies or reviews. We used Medical Subject Headings (MeSH) to build specific search strategies for the electronic databases (see **Table S2 in Additional file 2**). Only full-texts published in English were considered.

Group members assigned to a specific sub-topic independently assessed the results of electronic searches, identified relevant papers from the search results as well as additional relevant papers that were not identified via the literature search (see **Table S2 in Additional file 2**). Each record was screened and matching studies were grouped according to their study design (randomized controlled trial (RCT), observational studies / case series, systematic, scoping or narrative reviews and other manuscripts).

Then, additional titles were identified from reference lists of identified papers or via related articles feature in PubMed. In case the search of electronic databases did not allow identification of relevant papers for a specific topic, key words were modified in cooperation with the methodologist (CB) and the electronic search was newly performed.

Summary of evidence on each sub-topic is presented in subsections 1.1 to 5.6 and in Table 1. All details on available evidence and its identification are presented in Additional file 2, including key words and results for literature searches for different sub-questions (**Table S2)**, summary of evidence (**Table S3)** and all papers identified as relevant. All the co-authors were asked to suggest on all the parts of the manuscript including identification of additional references. Divergent opinions were discussed within the group with the aim to formulate an agreement of the group. The final view presented in the manuscript presents consensus of all co-authors.

*Identification of areas of uncertainty and development of specific study proposals*

All the co-authors identified areas of uncertainty for the sub-topic/s assigned to them. All the co-authors were encouraged to suggest on all of initially proposed areas of uncertainty.

All the co-authors were encouraged to propose new study ideas not limited to research question/s allocated to this co-author. Collected study ideas were formulated more in detail and refined based on suggestions from all the co-authors. Where possible and reasonable, individually proposed study projects were merged. We did not aim to necessarily follow initial structure of sub-topics allowing study projects to cross the boarders of initial list of sub-topics identified for literature search. All the co-authors agreed with the final list of study proposals.

*Voting procedure*

All study proposals (as presented in **Additional file 3, Table S4**) were taken for voting as equal entities independent of the sub-topic/s.

For the voting process all MEN section members were invited in addition to the co-authors. Voting was a two-step procedure, where Voting 1 was a shortlisting of the proposals, and Voting 2 was a quality assessment of the highest ranked proposals. All responders to Voting 1 received an invitation for participation in Voting 2.

During Voting 1 each participant was asked to select 5 study projects of priority. Study proposals were ranked based on the total number of votes received during Voting 1 and proposals ranked 1-20 were included in the list for Voting 2.

During Voting 2 each participant was asked to answer nine questions regarding each study proposal to identify feasibility and relevance, as well as likelihood to answer the formulated study question/hypothesis. Questions 1-2 were pointed out to the voters as the main questions giving the ranking for studies. Questions 3-9 were created to assist in reaching decisions on the main questions and their points used for final ranking in case of the same score for several study proposals after summarizing the points for the main questions. The scores for answer alternatives were blinded to the voters. Ten studies receiving the highest point score in total regarding respective items were identified as the *Top Ten* study proposals.

**Questions for Voting 2:**

1. **The study is likely to answer the hypothesis/study question**

- **yes (5 points)**
- **no (0 points)**
- **not sure (0 point)**

1. **General evaluation: This study**

- **is relevant and feasible (5 points)**
- **is relevant but not feasible due to e.g. lacking definitions, lacking tools, very high expense (1 point)**
- **is feasible but not clinically relevant (0 points)**

1. The risk of drop-outs is

- low (2 points)
- moderate (0 points)
- high (0 points)

1. Expected heterogeneity (related to unit, country etc.) is

- small (1 point)
- moderate (0 points)
- large (0 points)

1. Inclusion criteria are

- well defined (2 points)
- poorly defined (0 points)
- somewhat unclear (0 points)

1. The target population (to whom the results will be applied) is

- similar to study population (1 point)
- somewhat different from study population (1 point)
- very different from study population (0 points)

1. The study intervention or studied association is

- well defined (2 points)
- poorly defined (0 points)
- somewhat unclear (0 points)

1. Outcome(s) is(are)

- well defined (2 points)
- poorly defined (0 points)
- somewhat unclear (0 points)

1. Outcomes are

- relevant (2 points)
- not relevant (0 points)
- possibly relevant (0 points)

**Authors’ contributions**

All the co-authors participated in development of the outline (including research questions) and structure of the manuscript and approved the final version of the manuscript. ARB led the process and drafted the initial manuscript using contributions of co-authors (details listed below). AMD, SMJ, MPC, JG, AW, MB and YA revised various iterations of the manuscript.

CB created and performed all literature searches based on research questions. JS and ARB performed assessment of literature and drafted the manuscript parts on ‘clinical assessment’, ‘imaging’, ‘other monitoring’ and ‘systemic management’. CS and AvZ performed assessment of literature and drafted the manuscript part on ‘biomarkers’ and created Table S5. JW and OR performed assessment of literature and drafted the manuscript part on ‘Absorption of nutrients’ and drafted text of Additional file 4. JSch and IL performed assessment of literature and drafted the manuscript parts on ‘Monitoring of barrier function’ and ‘Mucosal integrity as a pathophysiological mechanism in GI dysfunction’. GE and MK performed assessment of literature and drafted the manuscript parts on ‘Monitoring of microbiome’ and ‘Microbiome in pathophysiology of GI dysfunction’. CS and ARB performed assessment of literature and drafted the manuscript part on ‘GI function and outcome’. SF and CL performed assessment of literature and drafted the manuscript part on ‘GI motility drugs’ and created Table 3. DB and ARB performed assessment of literature and drafted the manuscript part on ‘postpyloric feeding’. HOvS and DB performed assessment of literature and drafted the manuscript part on ‘GI function and nutrition’. JCP and LN performed assessment of literature and drafted the manuscript part on ‘GI function and multiple organ dysfunction’. JCP and AMD performed assessment of literature and drafted the manuscript part on ‘GI hormones’. VF and ARB performed assessment of literature and drafted the manuscript part on ‘bile acid signaling’. HOvS and ARB performed assessment of literature and drafted the manuscript part on ‘other pathophysiological mechanisms’.

JW, AW and ARB created questions and scoring system for ranking of study proposals.

CB and ARB drafted Tables S2 and S3. GE, HOvS, JCP, JG, and ARB drafted Table 2 and Table S4, based on study proposals provided by all co-authors. JS, HOvS and ARB drafted Table S5. CS, AvZ and AMD drafted Table S6. SF drafted Table S7.

All the co-authors (except methodologist - CB) and collaborators completed two voting rounds to identify study proposals of the highest priority.

**Conflicts of interest**

ARB received speaker fees from Fresenius Kabi and Nestlé, and a study grant for University of Tartu from Fresenius Kabi. JCP received speaker fees from Fresenius, Nestlé and Nutricia. HOvS received research support and speaker fees from Fresenius Kabi, Nutricia and Nestlé. OR received speakers fees from Fresenius Kabi, Nestle, Baxter and Nutricia and is a consultant for Fresenius Kabi. GE has received lecture fees and travel support from Fresenius Kabi, Baxter and consulting fees and travel support from Cardinal Health, Fresenius Kabi and Nutricia. JS has received speaker fees from B. Braun and Fresenius Kabi. JW received speaker fees from Baxter, Fresenius, GE Health Care, Nestlé, and Nutricia. CS received travel support and speaker fees from BBRAUN, Baxter and Fresenius. MMB received speaker fees from Fresenius Kabi and Baxter, and consulting fees from Fresenius Kabi. MPC and JG received consultancy fees by VIPUN Medical that were paid to KU Leuven. DEB reports receiving advisory board fees, speaker fees and conference attendance support from Nutricia, Nestle Nutrition, BBraun, Baxter healthcare, Fresenius Kabi, and Abbott Nutrition and advisory board fees from Avanos and Cardinal Health. AvZ received honoraria for advisory board meetings, lectures, and travel expenses from Abbott, Baxter, BBraun, Danone-Nutricia, Fresenius Kabi, Mermaid, Lyric and Nestle -Novartis. Inclusion fees for patients in nutrition trials were paid to the local ICU research foundation. AMD or his institution has received honoraria from Baxter, Cardinal Health, Nestle and Takeda.

SMJ reported that the Department of Intensive Care Medicine, University Hospital Bern, has or has had in the past, research contracts with Abionic SA, AVA AG, CSEM SA, Cube Dx GmbH, Cyto Sorbents Europe GmbH, Edwards Lifesciences LLC, GE Healthcare, ImaCor Inc., MedImmune LLC, Orion Corporation, Phagenesis Ltd. and research & development/consulting contracts with Edwards Lifesciences LLC, Nestec SA, Wyss Zurich. The money was paid into a departmental fund; Dr Jakob received no personal financial gain. The Department of Intensive Care Medicine has received unrestricted educational grants from the following organizations for organizing a quarterly postgraduate educational symposium, the Berner Forum for Intensive Care (until 2015): Abbott AG, Anandic Medical Systems, Astellas, AstraZeneca, Bard Medica SA, Baxter, B | Braun, CSL Behring, Covidien, Fresenius Kabi, GSK, Lilly, Maquet, MSD, Novartis, Nycomed, Orion Pharma, Pfizer, Pierre Fabre Pharma AG (formerly known as RobaPharm). The Department of Intensive Care Medicine, University Hospital Bern, has received unrestricted educational grants from the following organizations for organizing bi-annual postgraduate courses in the fields of critical care ultrasound, management of ECMO and mechanical ventilation: Abbott AG, Anandic Medical Systems, Bard Medica SA., Bracco, Dräger Schweiz AG, Edwards Lifesciences AG, Fresenius Kabi (Schweiz) AG, Getinge Group Maquet AG, Hamilton Medical AG, Pierre Fabre Pharma AG (formerly known as RobaPharm), PanGas AG Healthcare, Pfizer AG, Orion Pharma, Teleflex Medical GmbH.

The other co-authors reported no conflict of interest.

**Table S1. Academic conflicts of interests.**

Each author discloses projects in the field of pre-defined topics with manuscript published or data collection finished within the last 2 years (identified as P) or current, defined as data being collected, protocol has been ethics approved or funding for the study has been obtained (identified as C).

|  | Number of subtopic (see Table S2 in Additional file 2) | | | | | | | | | | | | | | | | |
| --- | --- | --- | --- | --- | --- | --- | --- | --- | --- | --- | --- | --- | --- | --- | --- | --- | --- |
| **Author** | **i** | **ii** | **iii** | **iv** | **v** | **vi** | **vii** | **viii** | **ix** | **x** | **xi** | **xii** | **xiii** | **xiv** | **xv** | **xvi** | **xvii** |
| Reintam Blaser | P/C |  | C |  |  |  |  |  |  |  | C |  |  |  |  |  |  |
| Preiser | P |  |  |  |  |  |  |  |  |  |  |  |  |  |  |  |  |
| Fruhwald |  |  |  |  |  |  | C |  |  |  |  |  |  |  |  |  | C |
| Wilmer |  |  |  |  |  |  |  |  |  |  |  |  |  |  |  |  |  |
| Wernerman |  | P |  | C |  |  |  |  |  |  |  |  |  |  |  |  | P/C |
| Benstoem |  |  |  |  |  |  |  |  |  |  |  |  |  |  |  |  |  |
| Casaer |  |  |  |  |  | P/C |  |  |  |  | P/C |  |  |  |  |  |  |
| Starkopf | P/C |  | C |  |  |  | P/C |  |  |  |  |  |  |  |  |  |  |
| van Zanten | P | C |  |  |  |  | P |  | C |  | C |  |  |  | P |  | C |
| Rooyackers |  |  | C | P/C |  |  |  |  |  |  |  |  |  |  |  |  | P/C |
| Jakob | P/C |  | P |  |  | P |  |  |  |  |  | P |  |  |  |  |  |
| Loudet |  |  |  |  |  |  |  |  |  |  | P | P |  |  |  |  |  |
| Bear |  |  |  |  |  |  |  |  |  |  |  |  |  |  |  |  |  |
| Elke |  |  |  |  |  |  |  |  |  |  | P/C |  | C |  |  |  | C |
| Kott |  |  | C |  |  | P |  |  |  |  |  |  | C |  |  |  |  |
| Lautenschläger |  | C |  |  | P |  |  |  |  |  |  |  |  | P |  |  | P/C |
| Schäper |  |  |  |  | C |  |  |  |  |  |  | C |  | C |  |  |  |
| Gunst |  |  |  |  |  | P/C |  |  |  |  | P/C |  |  |  |  |  |  |
| Stoppe |  |  | P/C | P/C |  |  |  |  |  |  | C |  |  |  |  |  | P/C |
| Nobile |  |  |  |  |  |  |  |  |  |  |  |  |  |  |  |  |  |
| Fuhrmann |  |  |  |  |  |  |  |  |  |  |  |  |  |  |  |  |  |
| Berger | C | P/C |  |  |  |  |  |  |  |  |  |  |  |  |  |  |  |
| Oudemans |  |  |  |  |  |  |  |  |  |  |  |  |  |  |  |  |  |
| Arabi |  |  |  |  |  |  |  |  |  | P/C |  |  |  |  |  |  |  |
| Deane | P | P |  | P | P |  | P/C | P/C | P/C |  | P/C |  |  |  | P/C |  |  |

Legend: i - clinical assessment; ii – imaging; iii – laboratory (incl. biomarkers); iv – absorption of nutrients; v – barrier function; vi – other monitoring; vii – prokinetics; viii - laxatives; ix – postpyloric feeding; x - other management; xi – gastrointestinal function and nutrition; xii – the role of the gut in multiple organ failure; xiii – microbiome; xiv – bacterial translocation/mucosal integrity; xv – gastrointestinal hormones; xvi – bile acid signaling; xvii - other pathophysiological mechanisms
