# Supplementary material for: Gastrointestinal dysfunction in the critically ill: a systematic scoping review and research agenda proposed by the Section of Metabolism, Endocrinology and Nutrition of the European Society of Intensive Care Medicine
Source: Crit Care. 2020 May 15;24:224. doi: 10.1186/s13054-020-02889-4 (PMC7226709; doi:10.1186/s13054-020-02889-4)
Supplement: Supplementary file 2 — Additional file 2: Summary of evidence. This file presents results of systematic reviews, summary of evidence and references of all papers selected for full text assessment. Table S2. in this file presents search strategies and results of screening of titles/abstracts for each research topic. Table S3. in this file presents summary of evidence for each research topics, referring to selected papers supporting this summary. All papers selected for full text assessment are listed, papers supporting summary of evidence (presented in Table 3 in the main manuscript and Table S3 in this file) are marked with asterisk. [file 13054_2020_2889_MOESM2_ESM.docx]

**Additional file 2. Summary of evidence.**

Contents

1. Table S2. Literature search strategies and results…………page 1-2
2. Table S3. Summary of evidence…………………………..page 3-4
3. All references……………………………………………...page 5-40

**Table S2.** **Literature search strategies and results.**

| **Subtopic** | | **Key words and search strategy *** | **Identified titles**  **(original search + additional)** | **Relevant papers selected for full-text assessment** |
| --- | --- | --- | --- | --- |
| **1. Monitoring of GI function** | | | | |
| i. | Clinical assessment | #1 gastrointestinal OR digestive [T/A]  #2 symptom* OR signs OR function OR dysfunction OR intolerance* [T/A]  #3 #1 AND #2  #4 adult [MeSH]  #5 critical care OR critical illness OR intensive care unit [MeSH]  #6 #3 AND #4 AND #5  **Limitations: Clinical Trial, Observational Study, Review** | 642  (635+7) | 45 |
| ii. | Imaging | #1 gastrointestinal OR digestive [T/A]  #2 Diagnostic Imaging [MeSH]  #3 #1 AND #2  #4 adult [MeSH]  #5 critical care OR critical illness OR intensive care unit [MeSH]  #6 #4 AND #5 AND #6 | 45  (39 + 6) | 10 |
| iii. | Laboratory biomarkers | #1 gastrointestinal OR digestive [T/A]  #2 biomarkers [MeSH]  #3 #1 AND #2  #4 adult [MeSH]  #5 critical care OR critical illness OR intensive care unit [MeSH]  #6 #4 AND #5 AND #6 | 71  (52 + 19) | 34 |
| iv. | Absorption of nutrients | #1 absorption [T/A]  #2 nutrients [T/A]  #3 #1 AND #2  #4 critical care OR critical illness OR intensive care unit [MeSH]  #5 #3 AND #4 | 74  (51 + 23) | 33 |
| v. | Barrier function | #1 gastrointestinal OR digestive [T/A]  #2 barrier function [T/A]  #3 #1 AND #2  #4 critical care OR critical illness OR intensive care unit [MeSH]  #5 #3 AND #4 | 103  (64 + 39) | 50 |
| vi. | Other (incl. intra-abdominal pressure) | #1 gastrointestinal OR digestive [T/A]  #2 intra-abdominal pressure [T/A]  #3 #1 AND #2  #4 critical care OR critical illness OR intensive care unit [MeSH]  #5 #3 AND #4 | 34  (30 + 4) | 11 |
| **2. GI dysfunction: reporting and outcome** – no specific search was performed for this subtopic, all papers identified from other subtopics were screened | | | | |
| **3. Management of GI dysfunction** | | | | |
| vii. | Prokinetics | #1 prokinetic* [T/A]  #2 critical care OR critical illness OR intensive care unit [MeSH]  #3 #1 AND #2 | 192  (175 + 17) | 62 |
| viii. | Laxatives | #1 laxative* [T/A]  #2 critical care OR critical illness OR intensive care unit [MeSH]  #3 #1 AND #2 | 112  (95 + 17) | 21 |
| ix. | Postpyloric feeding | #1 postpyloric feeding OR post-pyloric feeding OR nasojejunal OR nasointestinal OR nasoduodenal OR nasoenteral [T/A] #2 critical care OR critical illness OR intensive care unit [MeSH] #3 #1 AND #2 | 278  (265 + 13) | 31 |
| x. | Other | #1 gastrointestinal OR digestive [T/A]  #2 symptom* OR signs OR function OR dysfunction OR intolerance* [T/A]  #3 #1 AND #2  #4 adult [MeSH]  #5 critical care OR critical illness OR intensive care unit [MeSH]  #6 #3 AND #4 AND #5  **Limitations: Clinical Trial, Observational Study, Review** | 581  (565 + 20) | 31 |
| **4.**  xi. | **GI function and nutrition** | #1 gastrointestinal OR digestive [T/A]  #2 symptom* OR signs OR function OR dysfunction OR intolerance* [T/A]  #3 nutrition OR feeding [T/A]  #4 critical care OR critical illness OR intensive care unit [MeSH]  #5 adult [MeSH]  #6 = #1 AND #2 AND #3 AND #4 AND #5 | 210  (196 + 14) | 28 |
| **5. Pathophysiological mechanisms in GI dysfunction relevant to outcome** | | | | |
| xii. | The role of the gut in multiple organ failure | #1 gut [T/A]  #2 multiple organ failure [MeSH]  #3 critical care OR critical illness OR intensive care unit [MeSH]  #4 #1 AND #2 AND #3 | 111  (97 + 14) | 40 |
| xiii. | Microbiome | #1 gastrointestinal OR digestive [T/A]  #2 symptom* OR signs OR function OR dysfunction OR intolerance* [T/A]  #3 microbiome [T/A]  #4 critical care OR critical illness OR intensive care unit [MeSH]  #5 #1 AND #2 AND #3 AND #4 | 43  (25 + 18) | 23 |
| xiv. | Bacterial translocation/mucosal integrity | #1 bacterial translocation [T/A]  #2 mucosal integrity [T/A]  #3 #1 OR #2  #4 critical care OR critical illness OR intensive care unit [MeSH]  #5 #3 AND #4 | 590  (541 + 49) | 82 |
| xv. | GI hormones | #1 gastrointestinal OR digestive [T/A]  #2 symptom* OR signs OR function OR dysfunction OR intolerance* [T/A]  #3 hormones [T/A]  #4 critical care OR critical illness OR intensive care unit [MeSH]  #5 #1 AND #2 AND #3 AND #4 | 65  (33 + 32) | 36 |
| xvi. | Bile acid signaling | #1 bile acid [T/A]  #2 critical care OR critical illness OR intensive care unit [MeSH]  #3 #1 AND #2 | 83  (63 + 20) | 26 |
| xvii. | Other | #1 gastrointestinal OR digestive [T/A]  #2 symptom* OR signs OR function OR dysfunction OR intolerance* [T/A]  #3 #1 AND #2  #4 adult [MeSH]  #5 critical care OR critical illness OR intensive care unit [MeSH]  #6 #3 AND #4 AND #5  **Limitations: Clinical Trial, Observational Study, Review** | 644  (635 + 9) | 10 |

All searches were limited to English language. Searches were performed in Pubmed, CENTRAL and Cochrane Database of Systematic Reviews. Additional titles were identified from references or via Related articles feature.

* Search i was screened for all subtopics to identify additional papers not identified via specific searches.

GI – gastrointestinal; MeSH - Medical Subject Headings; T/A – title/abstract

**Table S3. Summary of evidence in all predefined subtopics related to GI dysfunction**.

| **Subtopic** | | **Study questions** | **RCTs** | **Obser-vational studies/ case series** | **Systematic, scoping and narrative**  **reviews** | **Other manu-scripts** | **Main observations** |
| --- | --- | --- | --- | --- | --- | --- | --- |
| **Monitoring of GI dysfunction** | | | | | | | |
| i. | Clinical assessment | Can GI symptoms * and/or clinical signs be used to monitor GI function in critically ill patients? | 3 | 19 | 6 | 1 | - There is no gold standard for monitoring of GI function in critically ill  - GI symptoms and clinical signs may be used, and the number of GI symptoms is associated with increased mortality  - GI symptoms/signs have not been clearly correlated with other objective methods quantifying GI function |
| ii. | Imaging | Can imaging be used to monitor GI function in critically ill? | 0 | 3 | 2 | 1 | - No validated imaging method for bedside monitoring  - GI ultrasound is promising but requires further study |
| iii. | Laboratory (incl. biomarkers) | Can biomarkers be used to monitor GI function in critically ill? | 5 | 15 | 5 | 0 | - No biomarker is validated for clinical use  - Host-, disease-, and analytics-related factors may influence potential biomarkers of interest  - Most of the studies assess biomarkers of mesenteric ischemia or organ dysfunction |
| iv. | Absorption of nutrients | Can absorption of nutrients be measured to monitor GI function? | 6 | 9 | 1 | 0 | - No method to measure absorption is available for routine clinical use |
| v. | Barrier function | Can barrier function be measured to monitor GI function? | 1 | 20 | 6 | 2 | - No valid method to measure barrier function is available for routine clinical use. |
| vi. | Other (incl. intra-abdomi-nal pressure - IAP) | Which other monitoring methods can be used to monitor GI function? | 0 | 6 | 5 | 1 | - IAP can be easily measured and gives a numeric value reflecting abdominal compartment  - Association with GI function is unclear  - Grossly elevated and/or increasing IAP may necessitate discontinuation or reduction of EN |
| **Management of GI dysfunction** | | | | | | | |
| vii. | Prokinetics | -Do prokinetics improve upper GI mo-tility in critically ill?  - Do prokinetics improve lower GI mo-tility in critically ill?  - Does combined treatment of upper and lower GI intole-rance improve GI mo-tility in critically ill?  - Do prokinetics improve other clinically relevant outcomes? | 10 | 3 | 14 | 1 | Gastric emptying:  -Erythromycin accelerates gastric emptying and may be superior to metoclopramide  - the effect of combination metoclopramide and erythromycin is sustained for longer than either drug alone  Lower GI dysmotility: insufficient data  Combined treatment of upper and lower GI motility:  - insufficient data  Uncertainty with regards to:  - recommended dose of erythromycin (3x100 mg vs. 200-250 mg) and therapy duration  - repeated treatment with gastroprokinetics  - definition of lower GI intolerance/dysmotility  - effect on morbidity and mortality |
| viii. | Laxatives | Do laxatives improve GI function, morbidity and mortality in critically ill patients? | 4 | 2 | 2 | 2 | - Possible benefit of prophylactic therapy (polyethylene glycol, lactulose) regarding time to defecation, but not regarding complications  - Polyethylene glycol probably better than lactulose, suggested to reduce the incidence of Ogilvie’s Syndrome |
| ix. | Postpyloric feeding | Does postpyloric feeding improve GI function, morbidity and mortality in critically ill patients receiving EN? | 17 | 0 | 2 | 0 | - Postpyloric feeding may reduce the number of patients who develop ventilator associated pneumonia  - Mostly small studies in patients without feeding intolerance  - Heterogeneity of intervention, i.e. different location of tubes (duodenal and jejunal) pooled |
| x. | Other | Which other management improves GI function, morbidity and mortality in critically ill? | 8 | 3 | 7 | 0 | - None confirmed in critically ill in general  - In postoperative patients, ERAS protocol and epidural analgesia may improve GI motility  - Beneficial effect of any specific (e.g. restrictive) fluid management strategy on GI function has not been proven |
| **xi.** | **GI function and nutrition** | Does EN improve GI function, morbidity and mortality in critically ill? | 16 | 6 | 5 | 1 | - EN may preserve GI immunity and attenuate proinflammatory changes and bacterial overgrowth  - The quantity of nutrient absorbed with EN during critical illness is uncertain  - EN has not been shown to improve patient-centred outcomes |
| **Pathophysiological mechanisms in GI dysfunction relevant to outcome** | | | | | | | |
| xii. | The role of the gut in multiple organ failure | What is the evidence on the role of the GI dys-function in develop-ment and course of MOF? | 0 | 7 (1 included also ex-perimental data) | 9 | 1 | Indirect evidence supports a role of GI dysfunction in the development/ perpetuation of MODS suggested by associations between the severity of GI dysfunction and organ failures. |
| xiii. | Microbiome | What is the evidence on the role of the microbiome in GI dysfunction? | 0 | 6 | 9 | 7 | Observational data have shown an association between critical illness (severity) and change of the intestinal microbiome as compared to the healthy state ("dysbiosis"). Change in microbiome is suggested to be associated with GI dysfunction and clinical outcome, but has yet to be confirmed by adequately powered studies. |
| xiv. | Bacterial translocation/mucosal integrity | What is the evidence on bacterial translocation/mucosal integrity in GI dysfunction? | 2 | 12 | 5 | 3 | Reported associations between the presence of enteric bacteria or bacterial products in the circulation, presumably related to gut dysfunction and poor outcome. Gut microbiota or related products (e.g. DAMPs in lymphatic ducts, endotoxins in portal blood, etc) may trigger distant organ damage in GI dysfunction. |
| xv. | GI hormones | What is the evidence that endogenous GI hormones are important in modulating GI dysfunction? | 1 | 7 | 10 | 0 | A decrease in the plasma concentration of orexigenic hormones (e.g. ghrelin) and an increase of anorexigenic hormones (e.g. PYY) during the early phase has been observed. No direct correlation with the GI function has been reported. |
| xvi. | Bile acid signaling | What is the evidence on bile acid signaling in GI dysfunction? | 0 | 2 | 4 | 4 | Bile acid signalling as a mechanism of GI dysfunction has not been studied in adult critically ill patients, but increased levels of bile acids in circulation are associated with adverse outcome. |
| xvii. | Other | What is the evidence on other mechanisms in GI dysfunction? | 0 | 0 | 1 | 3 | Bowel oedema and bowel distension have not been studied in critically ill patients. Bowel oedema impaired motility in experimental study. |

Legend:

Papers summarized here are marked with asterisk in the reference list below, presented by respective subtopics.

* GI symptoms include vomiting/regurgitation, abdominal distension, GI bleeding, diarrohea, lower GI paralysis [Reintam Blaser A, Malbrain ML, Starkopf J, Fruhwald S, Jakob SM, De Waele J, Braun JP, Poeze M, Spies C (2012) Gastrointestinal function in intensive care patients: terminology, definitions and management. Recommendations of the ESICM Working Group on Abdominal Problems. Intensive Care Med. 2012 Mar;38(3):384-94. doi: 10.1007/s00134-011-2459-y.]. Expanded (if performed/possible to assess): nausea, abdominal pain, absence of bowel sounds, large GRV (>500 ml/6h), bowel dilatation (radiological), bowel wall thickening/bowel oedema (radiological).

DAMP – damage-associated molecular pattern; EN – enteral nutrition; ERAS – early recovery after surgery; IAH – intra-abdominal hypertension; IAP – intra-abdominal pressure; FI –feeding intolerance; GI – gastrointestinal; MODS – multiple organ dysfunction syndrome; RCT – randomized controlled trial.

**ALL references selected for full-text assessment**

Papers considered in Table 2 for summary of evidence are marked with asterisk.

Papers are categorised as follows:

**RCTs** – randomised controlled studies in critically ill adult patients (incl. studies with patients possibly treated in ICU (e.g abdominal surgery))

**Observational studies** – observational studies in critically ill adult patients (incl. studies with patients possibly treated in ICU (e.g. abdominal surgery))

**Reviews** – all review articles (including narrative, scoping and systematic reviews) and meta-analyses (including guidelines)

**Case series** – case series and observational studies with <10 patients

**Other** – all studies and reviews on populations other than critically ill adult patients, including experimental studies; book chapters; surveys; case reports, letters

**Topic 1 Clinical Assessment**

RCT = 8

Observational = 28

Case series = 2

Review = 7

Other = 1

Papers identified from electronic databases (39):

RCTs

1. Jakob SM et al (2017) A randomized controlled pilot study to evaluate the effect of an enteral formulation designed to improve gastrointestinal tolerance in the critically ill patient-the SPIRIT trial. Crit Care 21(1):140. doi: 10.1186/s13054-017-1730-1
2. * McClave SA et al (2005) Poor validity of residual volumes as a marker for risk of aspiration in critically ill patients. Crit Care Med 33(2):324-330
3. Mohammadpour ALI et al (2018) The effect of gastric gas emptying on the residual gastric volume in mechanically-ventilated intensive care unit patients fed through nasogastric tubes: a randomized, single-blind, clinical trial. Asian J Pharm Clin Res 11(9):492-495.
4. Momenfar F et al (2018) Studying the effect of abdominal massage on the gastric residual volume in patients hospitalized in intensive care units. J Intensive Care 6:47. doi: 10.1186/s40560-018-0317-5.
5. Montejo JC et al (2002) Multicenter, prospective, randomized, single-blind study comparing the efficacy and gastrointestinal complications of early jejunal feeding with early gastric feeding in critically ill patients. Crit Care Med 30(4):796-800
6. * Nguyen NQ (2007) Feed intolerance in critical illness is associated with increased basal and nutrient-stimulated plasma cholecystokinin concentrations. Crit Care Med 35(1):82-88. doi: 10.1097/01.CCM.0000250317.10791.6C
7. * Ozen, N et al (2016) Evaluation of the effect on patient parameters of not monitoring gastric residual volume in intensive care patients on a mechanical ventilator receiving enteral feeding: a randomized clinical trial. J Crit Care 33:137-144. doi: 10.1016/j.jcrc.2016.01.028
8. Pinilla JC et al (2001) Comparison of gastrointestinal tolerance to two enteral feeding protocols in critically ill patients: a prospective, randomized controlled trial. JPEN J Parenter Enteral Nutr 25(2):81-86. doi: 10.1177/014860710102500281

Observational studies

1. * Bourcier S et al (2016) Diagnosis of non-occlusive acute mesenteric ischemia in the intensive care unit. Ann Intensive Care 6(1):112. doi: 10.1186/s13613-016-0213-x
2. Chang RW et al (1987) Gastrointestinal dysfunction among intensive care unit patients. Crit Care Med 15(10):909-914
3. Chen YD et al (2011) Monitoring of medical complications after acute ischemic stroke in a neurological intensive care unit. Eur Neurol 66(4):204-209. doi: 10.1159/000330551
4. Destrebecq AL et al (2014) Aerophagia increases the risk of ventilator-associated pneumonia in critically-ill patients. Minerva Anestesiol 80(4):410-418.
5. Edrisi F et al. (2018) Evaluation of patient’s energy intake between different types of formulas in the first week of starting enteral feeding in intensive care unit patients. Razavi Int Med 6(3): e57991
6. * Gungabissoon U et al (2015) Prevalence, risk factors, clinical consequences, and treatment of enteral feed intolerance during critical illness. JPEN J Parenter Enteral Nutr 39(4):441-448. doi: 10.1177/0148607114526450
7. * Hu B et al (2017) Severity of acute gastrointestinal injury grade is a predictor of all-cause mortality in critically ill patients: a multicenter, prospective, observational study. Crit Care 21(1):188. doi: 10.1186/s13054-017-1780-4
8. * Li H et al (2017) Association between acute gastrointestinal injury and biomarkers of intestinal barrier function in critically ill patients. BMC Gastroenterol 17(1):45. doi: 10.1186/s12876-017-0603-z
9. * Li H et al (2016) Association between acute gastrointestinal injury grading system and disease severity and prognosis in critically ill patients: A multicenter, prospective, observational study in China. J Crit Care 36:24-28. doi: 10.1016/j.jcrc.2016.05.001
10. * Marshall JC et al (1993) The gastrointestinal tract. The "undrained abscess" of multiple organ failure. Ann Surg 218(2):111-119
11. * Mentec, H et al (2001) Upper digestive intolerance during enteral nutrition in critically ill patients: frequency, risk factors, and complications. Crit Care Med 29(10):1955-1961
12. * Mierdl S et al (2001) Abdominal complications after cardiac surgery. Ann Acad Med Singapore 30(3):245-249
13. * Montejo JC (1999) Enteral nutrition-related gastrointestinal complications in critically ill patients: a multicenter study. The Nutritional and Metabolic Working Group of the Spanish Society of Intensive Care Medicine and Coronary Units." Crit Care Med 27(8):1447-1453
14. * Nassar AP Jr et al (2009) Constipation in intensive care unit: incidence and risk factors. J Crit Care 24(4):630.e639-612. doi: 10.1016/j.jcrc.2009.03.007
15. * Nguyen T et al (2013) Impaired gastrointestinal transit and its associated morbidity in the intensive care unit. J Crit Care 28(4):537.e511-537. doi: 10.1016/j.jcrc.2012.12.003
16. * Patejdl R et al (2017) Clinical nutrition and gastrointestinal dysfunction in critically ill stroke patients. Neurol Res 39(11):959-964. doi: 10.1080/01616412.2017.1367545
17. Pinto TF et al (2012) Tolerance to enteral nutrition therapy in traumatic brain injury patients. Brain Inj 26(9):1113-1117. doi: 10.3109/02699052.2012.666369
18. * Reintam A et al (2009) Gastrointestinal symptoms in intensive care patients. Acta Anaesthesiol Scand 53(3):318-324. doi: 10.1111/j.1399-6576.2008.01860.x
19. * Reintam A et al (2008) Gastrointestinal failure score in critically ill patients: a prospective observational study. Crit Care 12(4):R90. doi: 10.1186/cc6958
20. * Reintam A et al (2006) Gastrointestinal failure in intensive care: a retrospective clinical study in three different intensive care units in Germany and Estonia. BMC Gastroenterol 6:19. doi: 10.1186/1471-230X-6-19
21. * Reintam Blaser A et al (2013) Gastrointestinal symptoms during the first week of intensive care are associated with poor outcome: a prospective multicentre study. Intensive Care Med 39(5):899-909. doi: 10.1007/s00134-013-2831-1
22. * Reintam Blaser A et al (2015) Comparison of different definitions of feeding intolerance: A retrospective observational study. Clin Nutr 34(5):956-961. doi: 10.1016/j.clnu.2014.10.006
23. Rice TW et al (2005) Variation in enteral nutrition delivery in mechanically ventilated patients. Nutrition 21(7-8):786-792. DOI: 10.1016/j.nut.2004.11.014
24. Streefkerk JO et al (2016) Gastric feeding intolerance is not caused by mucosal ischemia measured by intragastric air tonometry in the critically ill. Clin Nutr 35(3):731-734. doi: 10.1016/j.clnu.2015.05.015
25. Torgersen C et al (2009) Macroscopic postmortem findings in 235 surgical intensive care patients with sepsis. Anesth Analg 108(6):1841-1847. doi: 10.1213/ane.0b013e318195e11d
26. * Zhang D et al (2014) Evaluation of clinical application of ESICM acute gastrointestinal injury grading system: a single-center observational study. Chin Med J (Engl) 127(10):1833-1836
27. Zhang X et al (2018) Effect of Rhubarb on Gastrointestinal Dysfunction in Critically Ill Patients: A Retrospective Study Based on Propensity Score Matching. Chin Med J (Engl) 131(10):1142-1150. doi: 10.4103/0366-6999.231523

Case series

1. * Goto J et al (2015) Usefulness of a real-time bowel sound analysis system in patients with severe sepsis (pilot study). J Artif Organs 18(1):86-91. doi: 10.1007/s10047-014-0799-4
2. * Marvin RG et al (2000) Nonocclusive bowel necrosis occurring in critically ill trauma patients receiving enteral nutrition manifests no reliable clinical signs for early detection." Am J Surg 179(1):7-12

Reviews

1. * Btaiche IF et al (2010) Critical illness, gastrointestinal complications, and medication therapy during enteral feeding in critically ill adult patients. Nutr Clin Pract 25(1):32-49. doi: 10.1177/0884533609357565
2. * Reintam Blaser A et al (2016) Gastrointestinal failure in the ICU. Curr Opin Crit Care 22(2):128-141. doi: 10.1097/MCC.0000000000000286

Additional papers identified (7):

Observational studies

1. * Mayr VD et al (2006) Causes of death and determinants of outcome in critically ill patients. Crit Care 10(6):R154. doi: 10.1186/cc5086

Reviews

1. * Asrani VM et al (2019) Gastrointestinal Dysfunction in Critical Illness: A Review of Scoring Tools. JPEN J Parenter Enteral Nutr 2019 Jul 26. doi: 10.1002/jpen.1679. [Epub ahead of print]
2. * D'Hondt A et al (2017) Uncontrolled bleeding of the gastrointestinal tract. Curr Opin Crit Care 23(6):549-555. doi: 10.1097/MCC.0000000000000452.
3. Lefering R et al (2002) Revision of the multiple organ failure score. Langenbecks Arch Surg 387(1):14-20. doi: 10.1007/s00423-001-0269-3
4. * Moonen PJ et al (2018) The black box revelation: monitoring gastrointestinal function. Anaesthesiol Intensive Ther 50(1):72-81. doi: 10.5603/AIT.a2017.0065
5. * Reintam Blaser A et al (2012) Gastrointestinal function in intensive care patients: terminology, definitions and management. Recommendations of the ESICM Working Group on Abdominal Problems. Intensive Care Med 38(3):384-94. doi: 10.1007/s00134-011-2459-y.

Other

1. * Berger MM et al (2008) Gastrointestinal failure score in critically ill patients. Crit Care (6):436. doi: 10.1186/cc7120

**Topic 2 Imaging**

RCT = 0

Observational = 7

Review = 2

Case series = 0

Other = 1

Identified from electronic databases (4):

Observational studies

1. Andrews AH et al (2005) Ineffectiveness of routine abdominal radiography in patients with gastrointestinal hemorrhage admitted to an intensive care unit. J Clin Gastroenterol 39(3):228-31
2. Gok F et al. (2015) Ultrasound-guided nasogastric feeding tube placement in critical care patients. Nutr Clin Pract 30(2):257-260. doi: 10.1177/0884533614567714
3. Rivera R et al (2011) Small bowel feeding tube placement using an electromagnetic tube placement device: accuracy of tip location. JPEN J Parenter Enteral Nutr 35(5):636-642. doi: 10.1177/0148607110386047
4. Zatelli M, Vezzali N (2017) 4-Point ultrasonography to confirm the correct position of the nasogastric tube in 114 critically ill patients. J Ultrasound 20(1):53-58. doi: 10.1007/s40477-016-0219-0

Additional papers identified (6):

Observational studies

1. * Dupont G et al (2017) Ultrasonographic gastric volume before unplanned surgery. Anaesthesia 72(9):1112-1116. doi: 10.1111/anae.13963
2. * Hamada SR et al (2014) Ultrasound assessment of gastric volume in critically ill patients. Intensive Care Med 40(7):965-72. doi: 10.1007/s00134-014-3320-x
3. * Sharma V et al (2017) Ultrasound-Assessed Gastric Antral Area Correlates With Aspirated Tube Feed Volume in Enterally Fed Critically Ill Patients. Nutr Clin Pract 32(2):206-211. doi: 10.1177/0884533616681530

Reviews

1. * Muresan C et al (2015) Abdominal Ultrasound for the Evaluation of Gastric Emptying Revisited. J Gastrointestin Liver Dis 24(3):329-38. doi: 10.15403/jgld.2014.1121.243.mur
2. * Perez-Calatayud AA et al (2018) Point-of-care gastrointestinal and urinary tract sonography in daily evaluation of gastrointestinal dysfunction in critically ill patients (GUTS Protocol). Anaesthesiol Intensive Ther 50(1):40-48. doi: 10.5603/AIT.a2017.0073

Other

1. * Richburg DA, Kim JH (2013) Real-time bowel ultrasound to characterize intestinal motility in the preterm neonate. J Perinatol 33(8):605-8. doi: 10.1038/jp.2013.17

**Topic 3 Laboratory**

RCT = 7

Observational = 18

Review = 5

Case series = 0

Other = 4

Papers identified from electronic databases (15):

RCTs

1. Bakiner O et al (2013) Impact of early versus late enteral nutrition on cell mediated immunity and its relationship with glucagon like peptide-1 in intensive care unit patients: a prospective study. Crit Care 17(3):R123. doi: 10.1186/cc12795
2. * Deane AM et al (2010) Effects of exogenous glucagon-like peptide-1 on gastric emptying and glucose absorption in the critically ill: relationship to glycemia. Crit Care Med 38(5):1261-1269. doi: 10.1097/CCM.0b013e3181d9d87a
3. Deane A M et al (2012) Randomized double-blind crossover study to determine the effects of erythromycin on small intestinal nutrient absorption and transit in the critically ill. Am J Clin Nutr 95(6):1396-402. doi: 10.3945/ajcn.112.035691
4. * Kar P et al (2015) Effects of glucose-dependent insulinotropic polypeptide on gastric emptying, glycaemia and insulinaemia during critical illness: a prospective, double blind, randomised, crossover study. Crit Care 19: 20. doi: 10.1186/s13054-014-0718-3
5. * Miller A et al (2017) Exogenous glucagon-like peptide-1 attenuates glucose absorption and reduces blood glucose concentration after small intestinal glucose delivery in critical illness. Crit Care Resusc 19(1):37-42
6. * Nguyen NQ et al (2012) Delayed enteral feeding impairs intestinal carbohydrate absorption in critically ill patients. Crit Care Med 40(1):50-54

Observational studies

1. * Chiolero RL et al (2003) Labeled acetate to assess intestinal absorption in critically ill patients. Crit Care Med 31(3):853-857. doi: 10.1097/01.CCM.0000055373.60390.8F
2. * Gao Y et al (2017) Procalcitionin as a diagnostic marker to distinguish upper and lower gastrointestinal perforation. World J Gastroenterol 23(24):4422-4427. doi: 10.3748/wjg.v23.i24.4422
3. * Gundling F et al (2011) Fecal calprotectin is a useful screening parameter for hepatic encephalopathy and spontaneous bacterial peritonitis in cirrhosis. Liver Int 31(9):1406-1415. doi: 10.1111/j.1478-3231.2011.02577.x
4. * Li H et al (2017). Association between acute gastrointestinal injury and biomarkers of intestinal barrier function in critically ill patients. BMC Gastroenterol 17(1):45. doi: 10.1186/s12876-017-0603-z
5. Oudemans-van Straaten H M et al (2011) Presence of tobramycin in blood and urine during selective decontamination of the digestive tract in critically ill patients, a prospective cohort study. Crit Care 15(5): R240. doi: 10.1186/cc10489
6. * Shimizu T et al (2013) Diagnostic potential of endotoxin scattering photometry for sepsis and septic shock. Shock 40(6):504-511. doi: 10.1097/SHK.0000000000000056
7. * Wierdsma NJ et al (2011) Malabsorption and nutritional balance in the ICU: fecal weight as a biomarker: a prospective observational pilot study. Crit Care 15(6):R264. doi: 10.1186/cc10530
8. * Zou L et al (2018) Intestinal fatty acid-binding protein as a predictor of prognosis in postoperative cardiac surgery patients. Medicine (Baltimore) 97(33):e11782. doi: 10.1097/MD.0000000000011782

Other

1. Bryant LK. et al (2004) Stimulation of small intestinal burst activity in the postprandial state differentially affects lipid and glucose absorption in healthy adult humans. Am J Physiol Gastrointest Liver Physiol 287(5):G1028-34. doi: 10.1152/ajpgi.00091.2004

Additional papers identified (19):

1. * Piton G et al (2019) Impact of the route of nutrition on gut mucosa in ventilated adults with shock: an ancillary of the NUTRIREA-2 trial. Intensive Care Med 45(7):948-956. doi: 10.1007/s00134-019-05649-3.

Observational studies

1. * Assadian A et al (2006) Plasma D-lactate as a potential early marker for colon ischaemia after open aortic reconstruction. Eur J Vasc Endovasc Surg 31(5):470-474. doi: 10.1016/j. ejvs.2005.10.031
2. * Block T et al (2008) Diagnostic accuracy of plasma biomarkers for intestinal ischaemia. Scand J Clin Lab Invest 68(3):242-248. doi: 10.1080/00365510701646264
3. * de Haan JJ et al (2009) Rapid development of intestinal cell damage following severe trauma: a prospective observational cohort study. Crit Care 13(3): R86. doi: 10.1186/cc7910
4. * de Madaria E et al (2005) Detection and identification of bacterial DNA in serum from patients with acute pancreatitis. Gut 54:1293-1297. doi: 10.1136/gut.2004.047514
5. * Fagoni N et al (2017) The IN-PANCIA Study: Clinical Evaluation of Gastrointestinal Dysfunction and Failure, Multiple Organ Failure, and Levels of Citrulline in Critically Ill Patients. J Intensive Care Med. 2017 Jan 1:885066617742594. doi: 10.1177/0885066617742594.
6. * Hong J et al (2017) Nonocclusive mesenteric infarction after cardiac surgery: potential biomarkers. J Surg Res 211: 21–29. doi: 10.1016/j.jss.2016.12.001
7. Oudemans-van Straaten HM et al (2002). Pitfalls in gastrointestinal permeability measurement in ICU patients with multiple organ failure using differential sugar absorption. Intensive Care Med 28(2):130-138. 10.1007/s00134-001-1140-2
8. * Poole A et al (2015) The relationship between fasting plasma citrulline concentration and small intestinal function in the critically ill. Crit Care 19:16. doi: 10.1186/s13054-014-0725-4.
9. * Shi H et al (2015) The role of serum intestinal fatty acid binding protein levels and D-lactate levels in the diagnosis of acute intestinal ischemia. Clin Res Hepatol Gastroenterol 39(3): 373–378. doi: 10.1016/j.clinre.2014.12.005
10. Wada T et al (2016) Early lactate clearance for predicting active bleeding in critically ill patients with acute upper gastrointestinal bleeding: a retrospective study. Intern Emerg Med 11(5):737-743. doi: 10.1007/s11739-016-1392-z

Reviews

1. * Crenn P et al (2008) Citrulline as a biomarker of intestinal failure due to enterocyte mass reduction. Clin Nutr 27:328-339. doi: 10.1016/j.clnu.2008.02.005
2. * Evennett NJ et al (2009) Systematic review and pooled estimates for the diagnostic accuracy of serological markers for intestinal ischemia. World J Surg 33:1374-1383. doi: 10.1007/s00268-009-0074-7
3. * Piton G et al (2011) Acute intestinal failure in critically ill patients: is plasma citrulline the right marker? Intensive Care Med 37:911-917. doi: 10.1007/s00134-011-2172-x
4. * Reintam Blaser A et al (2019) Citrulline and intestinal fatty acid-binding protein as biomarkers for gastrointestinal dysfunction in the critically ill. Anaesthesiol Intensive Ther 51(3):230-239. doi: 10.5114/ait.2019.86049
5. * Treskes N et al (2017) Diagnostic accuracy of novel serological biomarkers to detect acute mesenteric ischemia: a systematic review and meta-analysis. Intern Emerg Med 12(6):821-836. doi: 10.1007/s11739-017-1668-y

Other

1. Küçükaydin M et al (2000) Detection of intestinal bacterial translocation in subclinical ischemia-reperfusion using the polymerase chain reaction technique. J Pediatr Surg 35:41-43
2. Schellekens DH et al (2014) Plasma intestinal fatty acid-binding protein levels correlate with morphologic epithelial intestinal damage in a human translational ischemia-reperfusion model. J Clin Gastroenterol 48(3):253–260. doi: 10.1097/MCG.0b013e- 3182a87e3e
3. Shen LJ et al (2015) Serum citrulline as a diagnostic marker of sepsis-induced intestinal dysfunction. Clin Res Hepatol Gastroenterol 39(2):230–236. doi:10.1016/j.clinre.2014.10.002

**Topic 4 Absorption of nutrients**

RCT = 7

Observational = 12

Review = 6

Case series = 5

Other = 3

Papers identified from electronic databases (10):

RCTs

1. * Beale RJ et al (2008) Early enteral supplementation with key pharmaconutrients improves Sequential Organ Failure Assessment score in critically ill patients with sepsis: outcome of a randomized, controlled, double-blind trial. Crit Care Med 36(1):131-144. doi: 10.1097/01.CCM.0000297954.45251.A9
2. * Deane AM et al (2012) Randomized double-blind crossover study to determine the effects of erythromycin on small intestinal nutrient absorption and transit in the critically ill. Am J Clin Nutr 95(6):1396-402. doi: 10.3945/ajcn.112.035691
3. Heyland DK et al (2019) A multicenter, randomized, double-blind study of ulimorelin and metoclopramide in the treatment of critically ill patients with enteral feeding intolerance: PROMOTE trial. Intensive Care Med 45(5):647-656. doi: 10.1007/s00134-019-05593-2.
4. * Nguyen NQ et al (2012) Delayed enteral feeding impairs intestinal carbohydrate absorption in critically ill patients. Crit Care Med 40(1):50‐54 doi: 10.1097/CCM.0b013e31822d71a6

Observational studies

1. * Chiolero RL et al (2003) Labeled acetate to assess intestinal absorption in critically ill patients. Crit Care Med 31(3): 853-857. doi: 10.1097/01.CCM.0000055373.60390.8
2. * Di Bartolomeo AE et al (2012) Comparative effects on glucose absorption of intragastric and post-pyloric nutrient delivery in the critically ill. Crit Care 16(5): R167. doi: 10.1186/cc11522
3. * Strack van Schijndel RJ, Wierdsma NJ, van Heijningen EM, Weijs PJ, de Groot SD, Girbes AR (2006) Fecal energy losses in enterally fed intensive care patients: an explorative study using bomb calorimetry. Clin Nutr 25(5):758-764. doi: 10.1016/j.clnu.2005.11.012

Case series

1. Bryant LK et al (2004) Stimulation of small intestinal burst activity in the postprandial state differentially affects lipid and glucose absorption in healthy adult humans. Am J Physiol Gastrointest Liver Physiol 287(5): G1028-1034. doi: 10.1152/ajpgi.00091.2004

Reviews

1. Chapman MJ et al (2007) Gastrointestinal motility and prokinetics in the critically ill. Curr Opin Crit Care 13(2):187-194. doi: 10.1097/MCC.0b013e3280523a88
2. Leverve XM (2001) Inter-organ substrate exchanges in the critically ill. Curr Opin Clin Nutr Metab Care 4(2):137-142

Additional papers identified (23):

RCTs

1. * Deane AM et al (2010) Effects of exogenous glucagon-like peptide-1 on gastric emptying and glucose absorption in the critically ill: relationship to glycemia. Crit Care Med 38(5):1261-1269. doi: 10.1097/CCM.0b013e3181d9d87a
2. * Kar P et al (2015) Effects of glucose-dependent insulinotropic polypeptide on gastric emptying, glycaemia and insulinaemia during critical illness: a prospective, double blind, randomised, crossover study. Crit Care 19: 20. doi: 10.1186/s13054-014-0718-3
3. * Miller A et al (2017) Exogenous glucagon-like peptide-1 attenuates glucose absorption and reduces blood glucose concentration after small intestinal glucose delivery in critical illness. Crit Care Resusc 19(1):37-42

Observational studies

1. * Ali Abdelhamid Y et al (2015) Effect of Critical Illness on Triglyceride Absorption. JPEN J Parenter Enteral Nutr 39(8):966-72. doi: 10.1177/0148607114540214
2. * Berger MM, et al (2003) Serum paracetamol concentration: an alternative to X-rays to determine feeding tube location in the critically ill. JPEN J Parenter Enteral Nutr 27(2):151-5. doi: 10.1177/0148607103027002151
3. * Burgstad CM et al (2013) Sucrose malabsorption and impaired mucosal integrity in enterally fed critically ill patients: a prospective cohort observational study. Crit Care Med 41(5):1221-1228. doi: 10.1097/CCM.0b013e31827ca2fa
4. * Deane AM et al (2011) Glucose absorption and small intestinal transit in critical illness. Crit Care Med 39(6):1282-1288. doi: 10.1097/CCM.0b013e31820ee21f
5. * Deane AM et al (2014) The effects of critical illness on intestinal glucose sensing, transporters, and absorption. Crit Care Med 42(1):57-65. doi: 10.1097/CCM.0b013e318298a8af
6. Liebau F et al (2015) Short-term amino acid infusion improves protein balance in critically ill patients. Crit Care 19:106. doi: 10.1186/s13054-015-0844-6
7. Liebau F et al (2015) Effect of initiating enteral protein feeding on whole-body protein turnover in critically ill patients. Am J Clin Nutr 101(3):549-57. doi: 10.3945/ajcn.114.091934
8. * Pennings B et al (2012) Amino acid absorption and subsequent muscle protein accretion following graded intakes of whey protein in elderly men. Am J Physiol Endocrinol Metab 302(8):E992-999. doi: 10.1152/ajpendo.00517.2011
9. Sim JA et al (2013) Mesenteric blood flow, glucose absorption and blood pressure responses to small intestinal glucose in critically ill patients older than 65 years. Intensive Care Med 39(2):258-266. doi: 10.1007/s00134-012-2719-5

Reviews

1. Afolabi P et al (2013) Clinical utility of 13C-liver-function breath tests for assessment of hepatic function. Dig Dis Sci 58(1):33-41. doi: 10.1007/s10620-012-2340-z
2. Fuller MF, Reeds PJ (1998) Nitrogen cycling in the gut. Annu Rev Nutr 18:385-411. doi: 10.1146/annurev.nutr.18.1.385
3. * Gardiner K, Barbul A (1993) Intestinal amino acid absorption during sepsis. JPEN J Parenter Enteral Nutr 17(3):277-283. doi: 10.1177/0148607193017003277
4. Young VR (1986) Nutritional balance studies: indicators of human requirements or of adaptive mechanisms? J Nutr 116(4):700-703. doi: 10.1093/jn/116.4.700

Case series

1. Biolo G et al (1992) Leucine and phenylalanine kinetics during mixed meal ingestion: a multiple tracer approach. Am J Physiol 262(4 Pt 1):E455-463. doi: 10.1152/ajpendo.1992.262.4.E455
2. Fong Y et al (1994) Whole body and splanchnic leucine, phenylalanine, and glucose kinetics during endotoxemia in humans. Am J Physiol 266(2 Pt 2):R419-425. doi: 10.1152/ajpregu.1994.266.2.R419
3. Sundstrom Rehal M et al (2017) A supplemental intravenous amino acid infusion sustains a positive protein balance for 24 hours in critically ill patients. Crit Care 21(1):298. doi: 10.1186/s13054-017-1892-x
4. Vesali RF et al (2009) Protein metabolism in leg muscle following an endotoxin injection in healthy volunteers. Clin Sci (Lond) 2009, 118(6):421-427. doi: 10.1042/CS20090332

Other

1. Matthews DE et al (1993) Splanchnic bed utilization of glutamine and glutamic acid in humans. Am J Physiol 264(6 Pt 1):E848-854. doi: 10.1152/ajpendo.1993.264.6.E848
2. Matthews DE et al (1993) Splanchnic bed utilization of leucine and phenylalanine in humans. Am J Physiol 264(1 Pt 1):E109-118. doi: 10.1152/ajpendo.1993.264.1.E109
3. Wolfe RR, Chinkes DL (2004) Isotope Tracers in Metabolic Research: Principles and Practice of Kinetic Analysis, 2nd Edition, Wiley

**Topic 5 Barrier dysfunction monitoring**

RCT = 2

Observational = 26

Review = 9

Case series = 4

Other = 9

Papers identified from electronic databases (11):

Observational studies

1. * Angarita SAK et al (2019) Quantitative Measure of Intestinal Permeability Using Blue Food Coloring. J Surg Res 233:20-25. doi: 10.1016/j.jss.2018.07.005
2. * Holland J et al (2005) Intraoperative splanchnic hypoperfusion, increased intestinal permeability, down-regulation of monocyte class II major histocompatibility complex expression, exaggerated acute phase response, and sepsis. Am J Surg 190(3): 393-400. doi: 10.1016/j.amjsurg.2005.03.038
3. * Li H et al (2017) Association between acute gastrointestinal injury and biomarkers of intestinal barrier function in critically ill patients. BMC Gastroenterol 17(1): 45. doi: 10.1186/s12876-017-0603-z

Reviews

1. * Fink MP (1994) Effect of critical illness on microbial translocation and gastrointestinal mucosa permeability. Semin Respir Infect 9(4): 256-260.
2. McClave SA et al (2018) The 2016 ESPEN Arvid Wretlind lecture: The gut in stress. Clin Nutr 37(1): 19-36. doi: 10.1016/j.clnu.2017.07.015
3. * Piton G et al (2011) Acute intestinal failure in critically ill patients: is plasma citrulline the right marker? Intensive Care Med 37(6): 911-917. doi: 10.1007/s00134-011-2172-x
4. Salzman AL (1995) Nitric oxide in the gut. New Horiz 3(1): 33-45.
5. Steinmetz OK,and Meakins JL (1991) Care of the gut in the surgical intensive care unit: fact or fashion? Can J Surg 34(3): 207-215.

Other

1. * Han X et al (2004) Increased iNOS activity is essential for intestinal epithelial tight junction dysfunction in endotoxemic mice. Shock 21(3): 261-270. 10.1097/01.shk.0000112346.38599.10
2. O'Dwyer ST et al (1988) A single dose of endotoxin increases intestinal permeability in healthy humans. Arch Surg 123(12): 1459-1464. doi: 10.1001/archsurg.1988.01400360029003
3. * Rentea RM et al (2018). Role of intestinal Hsp70 in barrier maintenance: contribution of milk to the induction of Hsp70.2. Pediatr Surg Int 34(3): 323-330. doi: 10.1007/s00383-017-4211-3

Additional papers identified (39):

RCTs

1. * Besselink MG et al (2009) Intestinal barrier dysfunction in a randomized trial of a specific probiotic composition in acute pancreatitis. Ann Surg 250: 712-719. doi: 10.1097/SLA.0b013e3181bce5bd
2. Mangell P et al (2012) Lactobacillus plantarum 299v does not reduce enteric bacteria or bacterial translocation in patients undergoing colon resection. Dig Dis Sci 57: 1915-1924. doi: 10.1007/s10620-012-2102-y

Observational studies

1. Ambrose NS et al (1984) Incidence of pathogenic bacteria from mesenteric lymph nodes and ileal serosa during Crohn's disease surgery. Br J Surg 71: 623-625. doi: 10.1002/bjs.1800710821
2. * Deitch EA (1990) Intestinal permeability is increased in burn patients shortly after injury. Surgery 1990; 107: 411-416
3. Deitch EA (1989) Simple intestinal obstruction causes bacterial translocation in man. Arch Surg 124: 699-701. 10.1001/archsurg.1989.01410060065013
4. Derikx JPM et al (2007) Evidence for intestinal and liver epithelial cell injury in the early phase of sepsis. Shock 28:544–548. 10.1097/shk.0b013e3180644e32
5. Doig CJ et al (1998) Increased intestinal permeability is associated with the development of multiple organ dysfunction syndrome in critically ill ICU patients. Am.J.Respir.Crit Care Med 158: 444-451. 10.1164/ajrccm.158.2.9710092
6. * Grimaldi D et al (2013) Markers of intestinal injury are associated with endotoxemia in successfully resuscitated patients. Resuscitation 84(1):60-65. doi: 10.1016/j.resuscitation.2012.06.010
7. Guidet B et al (1994) Endotoxemia and bacteremia in patients with sepsis syndrome in the intensive care unit. Chest 106: 1194-1201. doi: 10.1378/chest.106.4.1194
8. Habes QLM et al (2017) Markers of Intestinal Damage and their Relation to Cytokine Levels in Cardiac Surgery Patients. Shock 47(6):709-714. doi: 10.1097/SHK.0000000000000803
9. * Harris CE et al (1992) Intestinal permeability in the critically ill. Intensive Care Med 18(1):38-41.
10. * Mizuno T et al (2010) Intraoperative bacterial translocation detected by bacterium-specific ribosomal rna-targeted reverse-transcriptase polymerase chain reaction for the mesenteric lymph node strongly predicts postoperative infectious complications after major hepatectomy for biliary malignancies. Ann Surg 252: 1013-1019. doi: 10.1097/SLA.0b013e3181f3f355
11. Moore FA (1991) Gut bacterial translocation via the portal vein: a clinical perspective with major torso trauma. J Trauma 31: 629-636
12. Moore FA et al (1992) Postinjury shock and early bacteremia. A lethal combination. Arch Surg 127: 893-897. 10.1001/archsurg.1992.01420080027004
13. * O’Boyle CJ et al (1998) Microbiology of bacterial translocation in humans. Gut 42:29–35. doi: 10.1136/gut.42.1.29
14. * Ono S et al (2005) Detection of microbial DNA in the blood of surgical patients for diagnosing bacterial translocation. World J Surg 29: 535-539. doi: 10.1007/s00268-004-7618-7
15. * Oudemans-van Straaten HM et al (2002) Pitfalls in gastrointestinal permeability measurement in ICU patients with multiple organ failure using differential sugar absorption. Intensive Care Med 28: 130-138. doi: 10.1007/s00134-001-1140-2
16. * Peitzman AB et al (1991) Bacterial translocation in trauma patients. J Trauma 31: 1083-1086
17. * Pijls KE et al (2014) Large intestine permeability is increased in patients with compensated liver cirrhosis. Am J Physiol Gastrointest Liver Physiol 306(2):G147-153. doi: 10.1152/ajpgi.00330.2013
18. * Piton G et al (2010) Plasma citrulline kinetics and prognostic value in critically ill patients. Intensive Care Med 36:702–706. doi: 10.1007/s00134-010-1751-6
19. * Piton G et al (2013) Enterocyte damage in critically ill patients is associated with shock condition and 28-day mortality. Crit Care Med 41:2169–2176. doi: 10.1097/CCM.0b013e31828c26b5
20. Reddy BS et al (2006) Surgical manipulation of the large intestine increases bacterial translocation in patients undergoing elective colorectal surgery. Colorectal Dis. 2006; 8: 596-600. doi: 10.1111/j.1463-1318.2006.01024.x
21. Roumen RM et al (1993) Intestinal permeability after severe trauma and hemorrhagic shock is increased without relation to septic complications. Arch Surg 128: 453-457. 10.1001/archsurg.1993.01420160095016
22. * Rush BF Jr et al (1988) Endotoxemia and bacteremia during hemorrhagic shock. The link between trauma and sepsis? Ann Surg 207: 549-554. doi: 10.1097/00000658-198805000-00009
23. * Timmermans K et al (2015) Circulating iFABP levels as a marker of intestinal damage in trauma patients. Shock 43:117–120. doi: 10.1097/SHK.0000000000000284

Reviews

1. * Berg RD (1999) Bacterial translocation from the gastrointestinal tract. Adv Exp Med Biol 473: 11-30. 10.1007/978-1-4615-4143-1_2
2. * Camilleri M et al (2012) Intestinal barrier function in health and gastrointestinal disease. Neurogastroenterol Motil 24(6):503-512. doi: 10.1111/j.1365-2982.2012.01921.x
3. * Tsujimoto H et al (2009) Role of translocation of pathogen-associated molecular patterns in sepsis. Dig Surg 26: 100-109. doi: 10.1159/000206143
4. * Wen Z et al (2019) A Protective Role of the NRF2-Keap1 Pathway in Maintaining Intestinal Barrier Function. Oxid Med Cell Longev 2019:1759149. doi: 10.1155/2019/1759149

Case series

1. * Hernandez G (2007) Splanchnic ischemia and gut permeability after acute brain injury secondary to intracranial hemorrhage. Neurocrit Care 7: 40-44. doi: 10.1007/s12028-007-0026-8
2. * Lemaire LC et al (1999) Thoracic duct in patients with multiple organ failure: no major route of bacterial translocation. Ann Surg 229: 128-36. doi: 10.1097/00000658-199901000-00017
3. * MacFie J et al (2006) Bacterial translocation studied in 927 patients over 13 years. Br J Surg 93: 87-93. doi: 10.1002/bjs.5184
4. * Sedman PC et al (1994) The prevalence of gut translocation in humans. Gastroenterology 107: 643-649

Other

1. Demaude J et al (2006) Phenotypic changes in colonocytes following acute stress or activation of mast cells in mice: implications for delayed epithelial barrier dysfunction. Gut 55: 655-661. doi: 10.1136/gut.2005.078675
2. Derikx JP et al (2008) New Insight in Loss of Gut Barrier during Major Non-Abdominal Surgery. PLoS One 3(12): e3954. doi: 10.1371/journal.pone.0003954
3. Hietbrink F et al (2009) Systemic inflammation increases intestinal permeability during experimental human endotoxemia. Shock 32: 374-378. doi: 10.1097/SHK.0b013e3181a2bcd6
4. Kramski M et al (2011) Novel sensitive real-time PCR for quantification of bacterial 16S rRNA genes in plasma of HIV-infected patients as a marker for microbial translocation. J Clin Microbiol 49: 3691-3693. doi: 10.1128/JCM.01018-11
5. Marchbank T et al (2011) The nutriceutical bovine colostrum truncates the increase in gut permeability caused by heavy exercise in athletes. Am J Physiol Gastrointest Liver Physiol 300: G477-G484. doi: 10.1152/ajpgi.00281.2010
6. Wen ZS et al (2019) Low Molecular Seleno-Aminopolysaccharides Protect the Intestinal Mucosal Barrier of Rats under Weaning Stress. Int J Mol Sci 20(22). pii: E5727. doi: 10.3390/ijms20225727

**Topic 6 Other monitoring**

**Results combined from searches on subtopic 1 and 6**

RCT = 0

Observational = 6

Review = 5

Case series = 0

Other = 1

Papers identified from electronic databases in search on Topic #6 (7):

Observational studies

1. * McNelis J et al (2002) Abdominal compartment syndrome in the surgical intensive care unit. Am Surg 68(1): 18-23.
2. * Reintam Blaser A et al (2011) Risk factors for intra-abdominal hypertension in mechanically ventilated patients. Acta Anaesthesiol Scand 55(5): 607-614. doi: 10.1111/j.1399-6576.2011.02415.x

Reviews

1. * Madl C, Druml W (2003) Gastrointestinal disorders of the critically ill. Systemic consequences of ileus. Best Pract Res Clin Gastroenterol 17(3): 445-456.
2. * Moonen PJ et al (2018) The black box revelation: monitoring gastrointestinal function. Anaesthesiol Intensive Ther 50(1): 72-81. doi: 10.5603/AIT.a2017.0065
3. * Reintam Blaser A et al (2017) Abdominal pressure and gastrointestinal function: an inseparable couple? Anaesthesiol Intensive Ther 49(2): 146-158. doi: 10.5603/AIT.a2017.0026
4. * Sheldon R, Eckert M (2017) Surgical Critical Care: Gastrointestinal Complications. Surg Clin North Am 97(6): 1425-1447. doi: 10.1016/j.suc.2017.08.002

Other

1. * Tanriverdi S et al (2013) Serial intravesical pressure measurements can predict the presence and the severity of necrotizing enterocolitis. Eur J Pediatr Surg 23(3): 243-248. doi: 10.1055/s-0032-1329706

Papers identified from electronic databases in search on Topic #1 (3):

Observational studies

1. * Heyland DK et al (1996) Impaired gastric emptying in mechanically ventilated, critically ill patients. Intensive Care Med 22(12): 1339-1344.
2. * Nguyen NQ et al (2007) The impact of admission diagnosis on gastric emptying in critically ill patients. Crit Care 11(1): R16. doi: 10.1186/cc5685
3. * Streefkerk JO et al (2016) Gastric feeding intolerance is not caused by mucosal ischemia measured by intragastric air tonometry in the critically ill. Clin Nutr 35(3): 731-734. doi: 10.1016/j.clnu.2015.05.015

Additional papers identified (2):

Observational studies

1. * Reintam Blaser A (2011) Intra-abdominal hypertension and gastrointestinal symptoms in mechanically ventilated patients. Crit Care Res Pract 2011:982507. doi: 10.1155/2011/982507.

Reviews

1. * Kirkpatrick AW et al (2013) Intra-abdominal hypertension and the abdominal compartment syndrome: updated consensus definitions and clinical practice guidelines from the World Society of the Abdominal Compartment Syndrome. Intensive Care Med 39(7): 1190–1206, doi: 10.1007/ s00134-013-2906-z

**Topic 7 Prokinetics**

RCT = 19

Observational = 13

Review = 20

Case series = 3

Other = 7

Papers identified from electronic databases (45):

RCTs

1. * Baradari AG et al (2017) Effects of combined prokinetic administration on gastric emptying in critically ill patients. Arab J Gastroenterol 18(1): 30-34. doi: 10.1016/j.ajg.2017.01.007
2. * Davis B, Ferrone M (2008) Prokinetic Therapy for Feed Intolerance in Critical Illness: One Drug or Two? Nutr Clin Pract 23(6): 660-661. doi: 10.1177/0884533608326322
3. * Heyland DK et al (2019) A multicenter, randomized, double-blind study of ulimorelin and metoclopramide in the treatment of critically ill patients with enteral feeding intolerance: PROMOTE trial. Intensive Care Med 45(5):647-656. doi: 10.1007/s00134-019-05593-2.
4. * Hu B et al (2015) Metoclopramide or domperidone improves post-pyloric placement of spiral nasojejunal tubes in critically ill patients: a prospective, multicenter, open-label, randomized, controlled clinical trial. Crit Care 19: 61. doi: 10.1186/s13054-015-0784-1
5. Malekolkottab M et al (2017) Metoclopramide as intermittent and continuous infusions in critically ill patients: a pilot randomized clinical trial. J Comp Eff Res 6(2): 127-136. doi: 10.2217/cer-2016-0067
6. * Nguyen NQ et al (2007) Prokinetic therapy for feed intolerance in critical illness: one drug or two? Crit Care Med 35(11): 2561-2567. doi: 10.1097/01.CCM.0000286397.04815.B1
7. * Nguyen NQ et al (2007) Erythromycin is more effective than metoclopramide in the treatment of feed intolerance in critical illness. Crit Care Med 35(2): 483-489. doi: 10.1097/01.CCM.0000253410.36492.E9
8. Spapen HD et al (1995) Gastric emptying in critically ill patients is accelerated by adding cisapride to a standard enteral feeding protocol: results of a prospective, randomized, controlled trial. Crit Care Med 23(3): 481-485.
9. Sustic A et al (2005) Metoclopramide improves gastric but not gallbladder emptying in cardiac surgery patients with early intragastric enteral feeding: randomized controlled trial. Croat Med J 46(2): 239-244.
10. * Taylor SJ et al (2016) A randomised controlled feasibility and proof-of-concept trial in delayed gastric emptying when metoclopramide fails: We should revisit nasointestinal feeding versus dual prokinetic treatment: Achieving goal nutrition in critical illness and delayed gastric emptying: Trial of nasointestinal feeding versus nasogastric feeding plus prokinetics. Clin Nutr ESPEN 14: 1-8. doi: 10.1016/j.clnesp.2016.04.020
11. van den Bosch S et al (2011) Erythromycin to promote bedside placement of a self-propelled nasojejunal feeding tube in non-critically ill patients having pancreatitis: a randomized, double-blind, placebo-controlled study. Nutr Clin Pract 26(2):181-185. doi: 10.1177/0884533611399924.
12. van der Spoel JI et al (2001) Neostigmine resolves critical illness-related colonic ileus in intensive care patients with multiple organ failure--a prospective, double-blind, placebo-controlled trial. Intensive Care Med 27(5): 822-827.

Observational studies

1. * Hersch M et al (2015) Prokinetic drugs for gastric emptying in critically ill ventilated patients: Analysis through breath testing. J Crit Care 30(3): 655.e657-613. doi: 10.1016/j.jcrc.2014.12.019
2. * Jooste CA et al (1999) Metoclopramide improves gastric motility in critically ill patients. Intensive Care Med 25(5): 464-468.
3. Landzinski J et al (2008) Gastric motility function in critically ill patients tolerant vs intolerant to gastric nutrition. JPEN J Parenter Enteral Nutr 32(1): 45-50. doi: 10.1177/014860710803200145
4. Martin M et al (2019) Nutrition During Targeted Temperature Management After Cardiac Arrest: Observational Study of Neurological Outcomes and Nutrition Tolerance. JPEN J Parenter Enteral Nutr. 2019 Apr 22. doi: 10.1002/jpen.1596. [Epub ahead of print]
5. * Nguyen NQ et al (2008) Risk of Clostridium difficile diarrhoea in critically ill patients treated with erythromycin-based prokinetic therapy for feed intolerance. Intensive Care Med 34(1): 169-173. doi: 10.1007/s00134-007-0834-5
6. Perez-Sanchez J et al (2017) Evaluation and handling of constipation in critical patients. Enferm Intensiva 28(4): 160-168. doi: 10.1016/j.enfi.2017.01.001
7. Ramos FL et al (2014) Azithromycin and COPD Exacerbations in the Presence or Absence of Symptoms or Active Treatment for Gastroesophageal Reflux. Chronic Obstr Pulm Dis 1(2): 221-228. doi: 10.15326/jcopdf.1.2.2014.0132
8. Stephens DP et al (2007) A clinical audit of the efficacy of tegaserod as a prokinetic agent in the intensive care unit. Crit Care Resusc 9(2): 148-150.
9. Taylor SJ et al (2010) Treating delayed gastric emptying in critical illness: metoclopramide, erythromycin, and bedside (cortrak) nasointestinal tube placement. JPEN J Parenter Enteral Nutr 34(3): 289-294. doi: 10.1177/0148607110362533

Reviews

1. * Chapman MJ et al (2007) Gastrointestinal motility and prokinetics in the critically ill. Curr Opin Crit Care 13(2): 187-194. doi: 10.1097/MCC.0b013e3280523a88
2. Davies AR, Bellomo R (2004) Establishment of enteral nutrition: prokinetic agents and small bowel feeding tubes. Curr Opin Crit Care 10(2): 156-161.
3. * Deane AM et al (2009) Prokinetic drugs for feed intolerance in critical illness: current and potential therapies. Crit Care Resusc 11(2): 132-143.
4. Diamond SJ et al (2017) In Search of the Ideal Promotility Agent: Optimal Use of Currently Available Promotility Agents for Nutrition Therapy of the Critically Ill Patient. Curr Gastroenterol Rep 19(12): 63. doi: 10.1007/s11894-017-0604-7
5. * Fraser RJ, Bryant L (2010) Current and future therapeutic prokinetic therapy to improve enteral feed intolerance in the ICU patient. Nutr Clin Pract 25(1): 26-31. doi: 10.1177/0884533609357570
6. * Fruhwald S et al (2007) Intestinal motility disturbances in intensive care patients pathogenesis and clinical impact. Intensive Care Med 33(1): 36-44. doi: 10.1007/s00134-006-0452-7
7. * Hawkyard CV, Koerner RJ (2007) The use of erythromycin as a gastrointestinal prokinetic agent in adult critical care: benefits versus risks. J Antimicrob Chemother 59(3): 347-358. doi: 10.1093/jac/dkl537
8. * Herbert MK, Holzer P (2008) Standardized concept for the treatment of gastrointestinal dysmotility in critically ill patients--current status and future options. Clin Nutr 27(1): 25-41. doi: 10.1016/j.clnu.2007.08.001
9. * Lewis K et al (2016) The efficacy and safety of prokinetic agents in critically ill patients receiving enteral nutrition: a systematic review and meta-analysis of randomized trials. Crit Care 20(1): 259. doi: 10.1186/s13054-016-1441-z
10. * Nguyen NQ (2014) Pharmacological therapy of feed intolerance in the critically ills. World J Gastrointest Pharmacol Ther 5(3): 148-155. doi: 10.4292/wjgpt.v5.i3.148
11. Roberts DJ et al (2016) Increased pressure within the abdominal compartment: intra-abdominal hypertension and the abdominal compartment syndrome. Curr Opin Crit Care 22(2): 174-185. doi: 10.1097/MCC.0000000000000289
12. * Rohm KD et al (2009) Motility disorders in the ICU: recent therapeutic options and clinical practice. Curr Opin Clin Nutr Metab Care 12(2): 161-167. doi: 10.1097/MCO.0b013e32832182c4
13. Stevens JE et al (2013) Pathophysiology and pharmacotherapy of gastroparesis: current and future perspectives. Expert Opin Pharmacother 14(9): 1171-1186. doi: 10.1517/14656566.2013.795948
14. Stupak DP et al (2012) Motility disorders of the upper gastrointestinal tract in the intensive care unit: pathophysiology and contemporary management. J Clin Gastroenterol 46(6): 449-456. doi: 10.1097/MCG.0b013e31824e14c1
15. van der Meer YG et al (2014) Should we stop prescribing metoclopramide as a prokinetic drug in critically ill patients? Crit Care 18(5): 502. doi: 10.1186/s13054-014-0502-4
16. * van Zanten AR (2016) Do we need new prokinetics to reduce enteral feeding intolerance during critical illness? Crit Care 20(1): 294. doi: 10.1186/s13054-016-1466-3

Case series

1. Banh HL et al (2005) The use of tegaserod in critically ill patients with impaired gastric motility. Clin Pharmacol Ther 77(6): 583-586. doi: 10.1016/j.clpt.2005.02.002
2. Clanton LJ Jr, Bender J (1999) Refractory spinal cord injury induced gastroparesis: resolution with erythromycin lactobionate, a case report. J Spinal Cord Med 22(4): 236-238. doi: 10.1080/10790268.1999.11719575
3. Komenaka IK et al (2000) Erythromycin and position facilitated placement of postpyloric feeding tubes in burned patients. Dig Surg 17(6): 578-580. doi: 10.1159/000051965

Other

1. Cook-Sather SD et al (2002) Cisapride does not prevent postoperative vomiting in children. Anesth Analg 94(1): 50-54. doi: 10.1097/00000539-200201000-00009
2. Eras Z et al (2013) Is metoclopramide safe for the premature infant? Eur Rev Med Pharmacol Sci 17(12): 1655-1657.
3. Ericson JE et al (2015) Use and Safety of Erythromycin and Metoclopramide in Hospitalized Infants. J Pediatr Gastroenterol Nutr 61(3): 334-339. doi: 10.1097/MPG.000000000000079
4. Rohm KD et al (2008). Nutrition support and treatment of motility disorders in critically ill patients - results of a survey on German intensive care units. Eur J Anaesthesiol 25(1): 58-66. doi: 10.1017/S0265021507002657
5. Woosley KP (2004) The problem of gastric atony. Clin Tech Small Anim Pract 19(1): 43-48. doi: 10.1053/S1096-2867(03)00083-5

Additional papers identified (17):

RCTs

1. Baradari AG et al (2016) A double-blind randomized clinical trial comparing the effect of neostigmine and metoclopramide on gastric residual volume of mechanically ventilated ICU patients. Acta informatica medica 24(6): 385-38. doi: 10.5455/aim.2016.24.385-389
2. * Berne JD et al (2002) Erythromycin reduces delayed gastric emptying in critically ill trauma patients: a randomized, controlled trial. J Trauma 53(3):422-425. doi: 10.1097/01.TA.0000019795.11972.FD
3. Chapman MJ et al (2016) The effect of camicinal (GSK962040), a motilin agonist, on gastric emptying and glucose absorption in feed-intolerant critically ill patients: a randomized, blinded, placebo-controlled, clinical trial. Crit Care 20(1): 232. doi: doi: 10.1186/s13054-016-1420-4
4. * Deane AM et al (2018) Nutrition Adequacy Therapeutic Enhancement in the Critically Ill: a Randomized Double-Blind, Placebo-Controlled Trial of the Motilin Receptor Agonist Camicinal (GSK962040): the NUTRIATE Study. JPEN J Parenter Enteral Nutr 42(5):949-959. doi: 10.1002/jpen.1038
5. * Deane AM et al (2012) Randomized double-blind crossover study to determine the effects of erythromycin on small intestinal nutrient absorption and transit in the critically ill. Am J Clin Nutr 95(6):1396-402. doi: 10.3945/ajcn.112.035691
6. MacLaren R et al (2000) Sequential single doses of cisapride, erythromycin, and metoclopramide in critically ill patients intolerant to enteral nutrition: a randomized, placebo-controlled, crossover study. Crit Care Med 28(2): 438-444.
7. MacLaren R et al (2001) Comparison of cisapride and metoclopramide for facilitating gastric emptying and improving tolerance to intragastric enteral nutrition in critically III, mechanically ventilated adults. Clin Ther 23(11): 1855-1866.

Observational studies (studies number 9 and 10 in this list are on opioid antagonists and not ‘prokinetics’)

1. Kram B et al (2018) Efficacy and Safety of Subcutaneous Neostigmine for Ileus, Acute Colonic Pseudo-obstruction, or Refractory Constipation. Ann Pharmacother 52(6):505-512. doi: 10.1177/1060028018754302
2. Merchan C et al (2017) Methylnaltrexone Versus Naloxone for Opioid-Induced Constipation in the Medical Intensive Care Unit. Ann Pharmacother 51(3):203-208. doi: 10.1177/1060028016677310
3. Sawh SB et al (2012) Use of methylnaltrexone for the treatment of opioid-induced constipation in critical care patients. Mayo Clin Proc 87(3):255-9. doi: 10.1016/j.mayocp.2011.11.014.
4. Smedley LW et al (2018) Safety and Efficacy of Intermittent Bolus and Continuous Infusion Neostigmine for Acute Colonic Pseudo-Obstruction. J Intensive Care Med 29:885066618809010. doi: 10.1177/0885066618809010

Reviews

1. * Fruhwald S et al (2008) Gastrointestinal motility in acute illness. Wien Klin Wochenschr 120(1-2):6-17. doi: 10.1007/s00508-007-0920-2
2. * Giudicessi JR et al (2018) Cardiovascular safety of prokinetic agents: A focus on drug-induced arrhythmias. Neurogastroenterol Motil 30(6):e13302. doi: 10.1111/nmo.13302
3. * Singer P et al (2018) ESPEN guideline on clinical nutrition in the intensive care unit. Clin Nutr 38(1):48-79. doi: 10.1016/j.clnu.2018.08.037
4. * Valle RG, Godoy FL (2014) Neostigmine for acute colonic pseudo-obstruction: A meta-analysis. Ann Med Surg (Lond) 3(3):60-64. doi: 10.1016/j.amsu.2014.04.002

Other

1. * Fruhwald S, Holzer P (2016) In: Webb A, Angus D, Finfer S, Gattinoni L, Singer M (eds) Gastrointestinal motility drugs in critical illness. Oxford Textbook of Critical Care, 2nd edn.: Oxford University Press, Oxford, pp 175-180
2. Thompson JS, Quigley EM (1999) Prokinetic agents in the surgical patient. Am J Surg 177(6):508-514. doi: 10.1016/s0002-9610(99)00104-x

**Topic 8 Laxatives**

RCT = 5

Observational = 2

Review = 5

Case series = 1

Other = 8

Papers identified from electronic databases (4):

RCTs

1. Alikiaii B, et al. (2019) Comparing the Efficacy of Two Drugs Senalin and Bisacodyl in Treatment of Constipation in Intensive Care Units' Patients. Adv Biomed Res. 8. Doi: 10.4103/abr.abr_165_18
2. * Hay T et al (2019) The hospital-based evaluation of laxative prophylaxis in ICU (HELP-ICU): a pilot cluster-crossover randomized clinical trial. J Crit Care. 52:86‐91. 10.1016/j.jcrc.2019.04.010
3. * Masri Y et al (2010) Prophylactic use of laxative for constipation in critically ill patients. Ann Thorac Med 5(4): 228-231. doi: 10.4103/1817-1737.69113

Reviews

1. * Hay T et al (2019) Constipation, diarrhea, and prophylactic laxative bowel regimens in the critically ill: A systematic review and meta-analysis. J Crit Care 52: 242-250. 10.1016/j.jcrc.2019.01.004

Additional papers identified (17):

RCTs

1. * de Azevedo RP et al (2015) Daily laxative therapy reduces organ dysfunction in mechanically ventilated patients: a phase II randomized controlled trial. Crit Care 19:329. doi: 10.1186/s13054-015-1047-x
2. * van der Spoel JI et al (2007) Laxation of critically ill patients with lactulose or polyethylene glycol: a two-center randomized, double-blind, placebo-controlled trial. Crit Care Med 35(12):2726-2731. doi: 10.1097/01.CCM.0000287526.08794.29

Observational studies

1. * Guardiola B et al (2016) Prophylaxis Versus Treatment Use of Laxative for Paralysis of Lower Gastrointestinal Tract in Critically Ill Patients. J Clin Gastroenterol 50(2):e13-18. doi: 10.1097/MCG.0000000000000316
2. Patanwala AE et al (2006) Pharmacologic management of constipation in the critically ill patient. Pharmacotherapy 26(7):896-902. doi: 10.1592/phco.26.7.896

Reviews

1. Fruhwald S et al. Gastrointestinal motility in acute illness. Wien Klin Wochenschr 120(1-2):6-17. doi: 10.1007/s00508-007-0920-2
2. Omer A, Quigley EMM (2017) An update on prucalopride in the treatment of chronic constipation. Therap Adv Gastroenterol 10(11):877-887. doi: 10.1177/1756283X17734809
3. * Thompson JS, Quigley EM (1999) Prokinetic agents in the surgical patient. Am J Surg 177(6):508-514. doi: 10.1016/s0002-9610(99)00104-x
4. Vazquez-Sandoval A et al (2017) Critically ill patients and gut motility: Are we addressing it? World J Gastrointest Pharmacol Ther 8(3):174-179. doi: 10.4292/wjgpt.v8.i3.174

Case series

1. * Haj M et al (2018) Ogilvie's syndrome: management and outcomes. Medicine (Baltimore) 97(27):e11187. doi: 10.1097/MD.0000000000011187.

Other

1. Bouras EP et al (2001) Prucalopride accelerates gastrointestinal and colonic transit in patients with constipation without a rectal evacuation disorder. Gastroenterology 120(2):354-360
2. Camilleri M et al (2016) Efficacy and Safety of Prucalopride in Chronic Constipation: An Integrated Analysis of Six Randomized, Controlled Clinical Trials. Dig Dis Sci 61(8):2357-2372. doi: 10.1007/s10620-016-4147-9
3. Cryer B et al (2017) Analysis of Nausea in Clinical Studies of Lubiprostone for the Treatment of Constipation Disorders. Dig Dis Sci 62(12):3568-3578. doi: 10.1007/s10620-017-4680-1
4. * Fruhwald S, Holzer P (2016) In: Webb A, Angus D, Finfer S, Gattinoni L, Singer M (eds) Gastrointestinal motility drugs in critical illness. Oxford Textbook of Critical Care, 2nd edn.: Oxford University Press, Oxford, pp 175-180
5. Okamura T et al (2017) Impact of lubiprostone on gastric-emptying profile and the possible effect of concomitant domperidone in healthy adults. Int J Clin Pharmacol Ther 55(11):861-865. doi: 10.5414/CP203014
6. Rey E et al (2017) Optimizing the Use of Linaclotide in Patients with Constipation-Predominant Irritable Bowel Syndrome: An Expert Consensus Report. Adv Ther 34(3):587-598. doi: 10.1007/s12325-016-0473-8
7. Spierings ELH et al (2017) Efficacy and Safety of Lubiprostone in Patients with Opioid-Induced Constipation: Phase 3 Study Results and Pooled Analysis of the Effect of Concomitant Methadone Use on Clinical Outcomes. Pain Med 19(6):1184-1194. doi: 10.1093/pm/pnx156
8. * Wald A (2007) Appropriate use of laxatives in the management of constipation. Curr Gastroenterol Rep 9(5):410-414

**Topic 9 Postpyloric feeding**

RCT = 22

Observational = 0

Review = 9

Case series = 0

Other = 0

Papers identified from electronic databases (18):

RCTs

1. * Davies AR et al (2002) Randomized comparison of nasojejunal and nasogastric feeding in critically ill patients. Crit Care Med 30(3): 586‐590. 10.1097/00003246-200203000-00016
2. * Davies AR et al (2012) A multicenter, randomized controlled trial comparing early nasojejunal with nasogastric nutrition in critical illness. Crit Care Med 40(8):2342-2348. doi: 10.1097/CCM.0b013e318255d87e
3. * Friedman G et al (2015) Randomized study to compare nasojejunal with nasogastric nutrition in critically ill patients without prior evidence of altered gastric emptying. Indian J Crit Care Med 19(2):71-75. doi: 10.4103/0972-5229.151013
4. Ge, W et al (2019) Nasointestinal Tube in Mechanical Ventilation Patients is More Advantageous. Open Med (WARS) 14: 426-430. doi: 10.1515/med-2019-0045.
5. * Heyland DK et al (2001) Effect of postpyloric feeding on gastroesophageal regurgitation and pulmonary microaspiration: results of a randomized controlled trial. Crit Care Med 29(8): 1495‐1501. doi: 10.1097/00003246-200108000-00001
6. * Hsu CW et al (2009) Duodenal versus gastric feeding in medical intensive care unit patients: a prospective, randomized, clinical study. Crit Care Med 37(6): 1866‐1872. doi: 10.1097/CCM.0b013e31819ffcda
7. * Huang HH et al (2012) Severity of illness influences the efficacy of enteral feeding route on clinical outcomes in patients with critical illness. J Acad Nutr Diet 112(8):1138-46. doi: 10.1016/j.jand.2012.04.013
8. * Montejo JC et al (2002) Multicenter, prospective, randomized, single-blind study comparing the efficacy and gastrointestinal complications of early jejunal feeding with early gastric feeding in critically ill patients. Crit Care Med 30(4): 796‐800. doi: 10.1097/00003246-200204000-00013
9. * Taylor SJ et al (2016) A randomised controlled feasibility and proof-of-concept trial in delayed gastric emptying when metoclopramide fails: we should revisit nasointestinal feeding versus dual prokinetic treatment: achieving goal nutrition in critical illness and delayed gastric emptying: trial of nasointestinal feeding versus nasogastric feeding plus prokinetics. Clin Nutr ESPEN 14: 1‐8. doi: 10.1016/j.clnesp.2016.04.020
10. Wan B et al (2015) Early jejunal feeding by bedside placement of a nasointestinal tube significantly improves nutritional status and reduces complications in critically ill patients versus enteral nutrition by a nasogastric tube. Asia Pac J Clin Nutr 24(1):51-7. doi: 10.6133/apjcn.2015.24.1.03
11. * Zhu Y et al (2018) Gastric versus postpyloric enteral nutrition in elderly patients (age >= 75 years) on mechanical ventilation: a single-center randomized trial. Crit Care 22(1):170. doi: 10.1186/s13054-018-2092-z

Reviews

1. Alkhawaja S et al (2015) Post-pyloric versus gastric tube feeding for preventing pneumonia and improving nutritional outcomes in critically ill adults. Cochrane Database Syst Rev (8): Cd008875. doi: 10.1002/14651858.CD008875.pub2
2. Drover JW. (2007). Gastric versus Postpyloric Feeding. Gastrointestinal endoscopy clinics of North America. 4: 765‐775. 10.1016/j.giec.2007.07.006
3. Ho KM et al (2006) A comparison of early gastric and postpyloric feeding in critically ill patients: a meta-analysis. Intensive Care Med 32(5): 639-649. doi: 10.1007/s00134-006-0128-3
4. Jiyong J et al (2013) Effect of gastric versus post-pyloric feeding on the incidence of pneumonia in critically ill patients: observations from traditional and Bayesian random-effects metaanalysis. Clin Nutr 32(1): 8-15. doi: 10.1016/j.clnu.2012.07.002
5. Marik PE and Zaloga GP (2003) Gastric versus post-pyloric feeding: a systematic review. Crit Care 7(3): R46-51. doi: 10.1186/cc2190
6. Sajid MS et al (2014) An integrated systematic review and meta-analysis of published randomized controlled trials evaluating nasogastric against postpyloris (nasoduodenal and nasojejunal) feeding in critically ill patients admitted in intensive care unit. Eur J Clin Nutr 68(4): 424-432. doi: 10.1038/ejcn.2014.6
7. Zhang Z et al (2013) Comparison of postpyloric tube feeding and gastric tube feeding in intensive care unit patients: a metaanalysis. Nutr Clin Pract 28(3): 371-380. doi: 10.1177/0884533613485987

Additional papers identified (13):

RCTs

1. * Acosta-Escribano J et al (2010) Gastric versus transpyloric feeding in severe traumatic brain injury: a prospective, randomized trial. Intensive Care Med 36:1532-1539. doi: 10.1007/s00134-010-1908-3
2. * Boivin MA, Levy H (2001) Gastric feeding with erythromycin is equivalent to transpyloric feeding in the critically ill. Crit Care Med 29:1916-1919. 10.1097/00003246-200110000-00011
3. * Day L et al (2001) Gastric versus duodenal feeding in patients with neurological disease: a pilot study. J Neurosci Nurs 33:148-149,155-159.
4. Esparza J et al (2001) Equal aspiration rates in gastrically and transpylorically fed critically ill patients. Intensive Care Med 27:660-664.
5. * Kearns PJ et al (2000) The incidence of ventilator-associated pneumonia and success in nutrient delivery with gastric versus small intestinal feeding: a randomized clinical trial. Crit Care Med 28:1742-1746. doi: 10.1097/00003246-200006000-00007
6. * Kortbeek JB et al (1999) Duodenal versus gastric feeding in ventilated blunt trauma patients: a randomized controlled trial. J Trauma 46:992-996
7. Kumar A et al (2006) Early enteral nutrition in severe acute pancreatitis: A prospective randomized controlled trial comparing nasojejunal and nasogastric routes. J Clin Gastroenterol 40:431-4.
8. * Montecalvo MA et al (1992) Nutritional outcome and pneumonia in critical care patients randomized to gastric versus jejunal tube feedings. The Critical Care Research Team. Crit Care Med 20:1377-1387. doi: 10.1097/00003246-199210000-00004
9. * Neumann DA, DeLegge MH (2002) Gastric versus small-bowel tube feeding in the Intensive Care Unit: A prospective comparison of efficacy. Crit Care Med 30:1436-1438. doi: 10.1097/00003246-200207000-00006
10. Singh N et al (2012) Evaluation of early enteral feeding through nasogastric and nasojejunal tube in severe acute pancreatitis: A noninferiority randomized controlled trial. Pancreas 41:153-159. doi: 10.1097/MPA.0b013e318221c4a8
11. * White H et al (2009) A randomised controlled comparison of early post-pyloric versus early gastric feeding to meet nutritional targets in ventilated intensive care patients. Crit Care 13: R187. doi: 10.1186/cc8181

Reviews:

1. * Deane AM et al (2013) Comparisons between intragastric and small intestinal delivery of enteral nutrition in the critically ill: a systematic review and meta-analysis. Crit Care 17(3):R125. doi: doi: 10.1186/cc12800
2. * Alhazzani W et al (2013) Small bowel feeding and risk of pneumonia in adult critically ill patients: a systematic review and meta-analysis of randomized trials. Crit Care 17(4):R127. doi: 10.1186/cc12806.

**Topic 10 Other management of GI dysfunction**

RCT = 12

Observational = 7

Review = 8

Case series = 1

Other = 3

Papers identified from electronic databases (11):

RCTs

1. * Dehghan M et al (2018) Does abdominal massage improve gastrointestinal functions of intensive care patients with an endotracheal tube?: A randomized clinical trial. Complement Ther Clin Pract 30: 122-128. doi: 10.1016/j.ctcp.2017.12.018
2. * McNaught CE et al (2005). A prospective randomised trial of probiotics in critically ill patients. Clin Nutr 24(2): 211‐219. doi: 10.1016/j.clnu.2004.08.008
3. * Meng JB et al (2018) Electroacupuncture Improves Intestinal Dysfunction in Septic Patients: a Randomised Controlled Trial. Biomed Res Int 2018:8293594. doi: 10.1155/2018/8293594
4. * Ralph CJ et al (2002) A randomised controlled trial investigating the effects of dopexamine on gastrointestinal function and organ dysfunction in the critically ill. Intensive Care Med 28(7): 884‐890. doi: 10.1007/s00134-002-1322-6
5. * Sun JJ et al (2013) Perirenal space blocking restores gastrointestinal function in patients with severe acute pancreatitis. World J Gastroenterol 19(46):8752-7. doi: 10.3748/wjg.v19.i46.8752
6. * Tyagi A et al (2017) Effect of thoracic epidural block on infection-induced inflammatory response: a randomized controlled trial. J Crit Care 38: 6‐12. doi: 10.1016/j.jcrc.2016.10.006

Observational studies

1. * Zhang X et al (2018) Effect of Rhubarb on Gastrointestinal Dysfunction in Critically Ill Patients: A Retrospective Study Based on Propensity Score Matching. Chin Med J (Engl) 131(10): 1142-1150. doi: 10.4103/0366-6999.231523
2. * Merchan C et al (2017) Methylnaltrexone Versus Naloxone for Opioid-Induced Constipation in the Medical Intensive Care Unit. Ann Pharmacother 51(3):203-208. doi: 10.1177/1060028016677310
3. * Sawh SB et al (2012) Use of methylnaltrexone for the treatment of opioid-induced constipation in critical care patients. Mayo Clin Proc 87(3):255-259. doi: 10.1016/j.mayocp.2011.11.014

Reviews

1. * Popping DM et al (2014) Impact of epidural analgesia on mortality and morbidity after surgery: systematic review and meta-analysis of randomized controlled trials. Ann Surg 259(6): 1056-1067. doi: 10.1097/SLA.0000000000000237
2. * Nee J et al (2018) Efficacy of Treatments for Opioid-induced Constipation: A Systematic Review and Meta-Analysis. Clin Gastroenterol Hepatol 16(10):1569-1584.e2. doi: 10.1016/j.cgh.2018.01.021

Additional papers identified (20):

RCTs

1. Kooshki A et al (2018) Effects of fenugreek seed powder on enteral nutrition tolerance and clinical outcomes in critically ill patients: A randomized clinical trial. Biomedical Research and Therapy 5(7): 2528-2537. DOI: 10.15419/bmrat.v5i7.462
2. * Lobo DN et al (2002) Effect of salt and water balance on recovery of gastrointestinal function after elective colonic resection: a randomised controlled trial. Lancet 359(9320):1812-1818. doi: 10.1016/S0140-6736(02)08711-1
3. Mohammadpour, ALI et al (2018) The effect of gastric gas emptying on the residual gastric volume in mechanically-ventilated intensive care unit patients fed through nasogastric tubes: a randomized, single-blind, clinical trial. Asian journal of pharmaceutical and clinical research 11(9): 492‐495.
4. Momenfar F et al Studying the effect of abdominal massage on the gastric residual volume in patients hospitalized in intensive care units. J Intensive Care. 2018 Aug 10;6:47. doi: 10.1186/s40560-018-0317-5. eCollection 2018.
5. * Myles PS et al (2018) Restrictive versus Liberal Fluid Therapy for Major Abdominal Surgery. N Engl J Med 378(24):2263-2274. doi: 10.1056/NEJMoa1801601.
6. Xing X et al (2019) Traditional Chinese medicine bundle therapy for septic acute gastrointestinal injury: A multicenter randomized controlled trial. Complement Ther Med 47:102194. doi: 10.1016/j.ctim.2019.102194.

Observational

1. Barletta JF et al (2011) Influence of intravenous opioid dose on postoperative ileus. Ann Pharmacother 45(7-8):916-923. doi: 10.1345/aph.1Q041
2. Malbrain ML et al (2012) Relationship between intra-abdominal pressure and indocyanine green plasma disappearance rate: hepatic perfusion may be impaired in critically ill patients with intra-abdominal hypertension. Ann Intensive Care 2 Suppl 1:S19. doi: 10.1186/2110-5820-2-S1-S19
3. Reintam Blaser A et al (2011) Intra-abdominal hypertension and gastrointestinal symptoms in mechanically ventilated patients. Crit Care Res Pract 2011:982507. doi: 10.1155/2011/982507
4. Reintam Blaser A et al (2019) Incidence, Risk Factors, and Outcomes of Intra-Abdominal Hypertension in Critically Ill Patients - A Prospective Multicenter Study (IROI Study). Crit Care Med 47(4):535-542. doi: 10.1097/CCM.0000000000003623.

Reviews

1. * Brigode WM et al (2015) Scrutinizing the evidence linking hypokalemia and ileus: A commentary on fact and dogma. Int J Acad Med 1:21-26
2. * Corcoran T, et al (2012) Perioperative fluid management strategies in major surgery: a stratified meta-analysis. Anesth Analg 114(3):640-51. doi: 10.1213/ANE.0b013e318240d6eb
3. * Kirkpatrick AW et al (2013) Intra-abdominal hypertension and the abdominal compartment syndrome: updated consensus definitions and clinical practice guidelines from the World Society of the Abdominal Compartment Syndrome. Intensive Care Med 39(7):1190-206. doi: 10.1007/s00134-013-2906
4. * Reintam Blaser A et al (2015) Diarrhoea in the critically ill. Curr Opin Crit Care 21(2):142-53. doi: 10.1097/MCC.0000000000000188
5. * Schol PB et al (2016) Liberal or restrictive fluid management during elective surgery: a systematic review and meta-analysis. J Clin Anesth 35:26-39. doi: 10.1016/j.jclinane.2016.07.010
6. * Wallström A, Frisman GH (2014) Facilitating early recovery of bowel motility after colorectal surgery: a systematic review. J Clin Nurs 23(1-2):24-44. doi: 10.1111/jocn.12258

Case series

1. Rubinoff MJ et al (1989) Clonidine prolongs human small intestine transit time: use of the lactulose-breath hydrogen test. Am J Gastroenterol 84:372-374

Other

1. Schvarcz E et al (1997) Physiological hyperglycemia slows gastric emptying in normal subjects and patients with insulin-dependent diabetes mellitus. Gastroenterology 113(1):60-66
2. Kreiss C et al (2004) Alpha2-adrenergic regulation of NO production alters postoperative intestinal smooth muscle dysfunction in rodents. Am J Physiol Gastrointest Liver Physiol 287(3):G658-666. 10.1152/ajpgi.00526.2003
3. Slatkin N et al (2009) Methylnaltrexone for treatment of opioid-induced constipation in advanced illness patients. J Support Oncol 7(1):39-46

**Topic 11 GI function and nutrition**

RCT = 16

Observational = 6

Review = 5

Case series = 0

Other = 1

Papers identified from electronic databases (14):

RCTs

1. * Hadfield RJ et al (1995) Effects of enteral and parenteral nutrition on gut mucosal permeability in the critically ill. Am J Respir Crit Care Med 152: 1545-1548. doi: 10.1164/ajrccm.152.5.7582291
2. * Kompan L et al (1999) Effects of early enteral nutrition on intestinal permeability and the development of multiple organ failure after multiple injury. Intensive Care Med 25: 157-161
3. * Kompan L et al (2004) Is early enteral nutrition a risk factor for gastric intolerance and pneumonia? Clin Nutr 23: 527‐532. doi: 10.1016/j.clnu.2003.09.013
4. * Lu K et al (2018) A more physiological feeding process in ICU: Intermittent infusion with semi-solid nutrients (CONSORT-compliant). 97: e12173. doi: 10.1097/MD.0000000000012173
5. * MacLeod JB et al (2007) Prospective randomized control trial of intermittent versus continuous gastric feeds for critically ill trauma patients. J Trauma 63: 57‐61. doi: 10.1097/01.ta.0000249294.58703.11
6. * Nasiri M et al (2017) Comparison of intermittent and bolus enteral feeding methods on enteral feeding intolerance of patients with sepsis: a triple-blind controlled trial in intensive care units. Middle East J Dig Dis 9(4):218-227. doi: 10.15171/mejdd.2017.77
7. * Petros S et al (2016) Hypocaloric vs Normocaloric Nutrition in Critically Ill Patients: A Prospective Randomized Pilot Trial. JPEN J Parenter Enteral Nutr 40: 242-249. doi: 10.1177/0148607114528980
8. * Qiu C et al (2017) Fat-Modified Enteral Formula Improves Feeding Tolerance in Critically Ill Patients: a Multicenter, Single-Blind, Randomized Controlled Trial. JPEN J Parenter Enteral Nutr 41(5):785-795. doi: 10.1177/0148607115601858
9. * Rice TW et al (2011) Randomized trial of initial trophic versus full-energy enteral nutrition in mechanically ventilated patients with acute respiratory failure. Crit Care Med 39: 967‐974. doi: 10.1097/CCM.0b013e31820a905a

Observational studies

1. * Kadamani I et al (2014) Incidence of aspiration and gastrointestinal complications in critically ill patients using continuous versus bolus infusion of enteral nutrition: a pseudo-randomised controlled trial. Aust Crit Care 27(4):188-93. doi: 10.1016/j.aucc.2013.12.001
2. * Montejo JC (1999) Enteral nutrition-related gastrointestinal complications in critically ill patients: a multicenter study. The Nutritional and Metabolic Working Group of the Spanish Society of Intensive Care Medicine and Coronary Units. Crit Care Med 27: 1447-1453. 10.1097/00003246-199908000-00006
3. * Serpa LF et al (2003) Effects of continuous versus bolus infusion of enteral nutrition in critical patients. Rev Hosp Clin Fac Med Sao Paulo 58(1):9-14
4. * Thibault R (2013) Diarrhoea in the ICU: respective contribution of feeding and antibiotics. Crit Care 17: R153. doi: 10.1186/cc12832

Reviews

1. * Fuentes Padilla P, et al. Early enteral nutrition (within 48 hours) versus delayed enteral nutrition (after 48 hours) with or without supplemental parenteral nutrition in critically ill adults. Cochrane Database Syst Rev. 2019 Oct 31;2019(10). doi: 10.1002/14651858.CD012340.pub2.

Additional papers identified (14):

RCTs

1. * Casaer MP et al (2011) Early versus late parenteral nutrition in critically ill adults. N Engl J Med 365(6):506-17. doi: 10.1056/NEJMoa1102662
2. * Doig GS et al (2015) Restricted versus continued standard caloric intake during the management of refeeding syndrome in critically ill adults: a randomised, parallel-group, multicentre, single-blind controlled trial. Lancet Respir Med 3(12):943-952. doi: 10.1016/S2213-2600(15)00418-X.
3. * El-Kersh K et al (2018) Enteral nutrition as stress ulcer prophylaxis in critically ill patients: a randomized controlled exploratory study. J Crit Care 43: 108-113. doi: 10.1016/j.jcrc.2017.08.036
4. * Harvey SE et al (2014) Trial of the route of early nutritional support in critically ill adults. N Engl J Med 371(18):1673-1684. doi: 10.1056/NEJMoa1409860
5. * Nguyen NQ et al (2012) Delayed enteral feeding impairs intestinal carbohydrate absorption in critically ill patients. Crit Care Med 40(1):50-54. doi: 10.1097/CCM.0b013e31822d71a6
6. * Reignier J etal (2018) Enteral versus parenteral early nutrition in ventilated adults with shock: a randomised, controlled, multicentre, open-label, parallel-group study (NUTRIREA-2). Lancet. 2018 Jan 13;391(10116):133-143. doi: 10.1016/S0140-6736(17)32146-3.
7. * Wang G et al (2013) Effect of enteral nutrition and ecoimmunonutrition on bacterial translocation and cytokine production in patients with severe acute pancreatitis. J Surg Res 183(2):592-7. doi: 10.1016/j.jss.2012.12.010

Observational studies

1. * Nguyen NQ et al (2011) Relationship between altered small intestinal motility and absorption after abdominal aortic aneurysm repair. Intensive Care Med 37(4):610-618. doi: 10.1007/s00134-010-2094-z
2. * Wierdsma NJ et al (2011) Malabsorption and nutritional balance in the ICU: fecal weight as a biomarker: a prospective observational pilot study. Crit Care 15(6):R264. doi: 10.1186/cc10530

Reviews

1. * Barrett M et al (2015) Intestine, immunity, and parenteral nutrition in an era of preferred enteral feeding. Curr Opin Clin Nutr Metab Care 18(5):496-500. doi: 10.1097/MCO.0000000000000208
2. * Casaer MP, Van den Berghe G (2014) Nutrition in the acute phase of critical illness. N Engl J Med 370(13):1227-1236. doi: 10.1056/NEJMra1304623
3. * Fraipont V, Preiser JC (2013) Energy estimation and measurement in critically ill patients. JPEN J Parenter Enteral Nutr 37(6):705-713. doi: 10.1177/0148607113505868
4. * Reintam Blaser A et al (2017) Early enteral nutrition in critically ill patients: ESICM clinical practice guidelines. Intensive Care Med 43(3):380-398. doi: 10.1007/s00134-016-4665-0

Other

1. * Ralls MW et al (2015) Enteral nutrient deprivation in patients leads to a loss of intestinal epithelial barrier function. Surgery 157(4):732-742. doi: 10.1016/j.surg.2014.12.004

**Topic 12 The gut and multiple organ failure**

RCT = 5

Observational = 9

Review = 20

Case series = 1

Other = 5

Papers identified from electronic databases (26):

RCTs

1. Alberda C et al (2007) Effects of probiotic therapy in critically ill patients: a randomized, double-blind, placebo-controlled trial. Am J Clin Nutr 85(3): 816-823. doi: 10.1093/ajcn/85.3.816
2. Cerra FB et al (1992) Selective gut decontamination reduces nosocomial infections and length of stay but not mortality or organ failure in surgical intensive care unit patients. Arch Surg 127(2): 163-167. 10.1001/archsurg.1992.01420020045007
3. Kompan L et al (1999) Effects of early enteral nutrition on intestinal permeability and the development of multiple organ failure after multiple injury. Intensive Care Med 25(2): 157-161.

Observational studies

1. * Border JR et al (1987) The gut origin septic states in blunt multiple trauma (ISS = 40) in the ICU. Ann Surg 206(4): 427-448. doi: 10.1097/00000658-198710000-00004
2. Jakob SM et al (2009) Increased splanchnic oxygen extraction because of routine nursing procedures. Crit Care Med 37(2): 483-489. doi: 10.1097/CCM.0b013e3181958821
3. * Marshall JC et al (1993) The gastrointestinal tract. The "undrained abscess" of multiple organ failure. Ann Surg 218(2): 111-119. doi: 10.1097/00000658-199308000-00001
4. * Mythen MG, Webb AR (1994) Intra-operative gut mucosal hypoperfusion is associated with increased post-operative complications and cost. Intensive Care Med 20(2): 99-104.
5. * Osuka A et al (2017) Acute intestinal damage following severe burn correlates with the development of multiple organ dysfunction syndrome: A prospective cohort study. Burns 43(4): 824-829. doi: 10.1016/j.burns.2016.10.015
6. * Piton G et al (2010) Plasma citrulline kinetics and prognostic value in critically ill patients. Intensive Care Med 36(4): 702-706. doi: 10.1007/s00134-010-1751-6
7. * Zou L et al (2018) Intestinal fatty acid-binding protein as a predictor of prognosis in postoperative cardiac surgery patients. Medicine (Baltimore) 97(33): e11782. doi: 10.1097/MD.0000000000011782

Reviews

1. * Clark JA, Coopersmith CM (2007) Intestinal crosstalk: a new paradigm for understanding the gut as the "motor" of critical illness. Shock 28(4): 384-393. doi: 10.1097/shk.0b013e31805569df
2. * Donati A et al (2016) The role of cardiac dysfunction in multiorgan dysfunction. Curr Opin Anaesthesiol 29(2): 172-177. doi: 10.1097/ACO.0000000000000296
3. Fink MP (2003) Intestinal epithelial hyperpermeability: update on the pathogenesis of gut mucosal barrier dysfunction in critical illness. Curr Opin Crit Care 9(2): 143-151.
4. Fink MP, Delude RL (2005) Epithelial barrier dysfunction: a unifying theme to explain the pathogenesis of multiple organ dysfunction at the cellular level. Crit Care Clin 21(2): 177-196. doi: 10.1016/j.ccc.2005.01.005
5. * Hedenstierna G, Larsson A (2012) Influence of abdominal pressure on respiratory and abdominal organ function. Curr Opin Crit Care 18(1): 80-85. doi: 10.1097/MCC.0b013e32834e7c3a
6. * Klingensmith NJ, Coopersmith CM (2016) The Gut as the Motor of Multiple Organ Dysfunction in Critical Illness. Crit Care Clin 32(2): 203-212. doi: 10.1016/j.ccc.2015.11.004
7. * Meng M et al (2017) New insights into the gut as the driver of critical illness and organ failure. Curr Opin Crit Care 23(2): 143-148. doi: 10.1097/MCC.0000000000000386
8. Patel JJ et al (2016) The gut in trauma. Curr Opin Crit Care 22(4): 339-346. doi: 10.1097/MCC.0000000000000331
9. * Piton G et al (2011) Acute intestinal failure in critically ill patients: is plasma citrulline the right marker? Intensive Care Med 37(6): 911-917. doi: 10.1007/s00134-011-2172-x
10. Puleo F et al (2011) Gut failure in the ICU. Semin Respir Crit Care Med 32(5): 626-638. doi: 10.1055/s-0031-1287871
11. Schmidt H, Martindale R (2003) The gastrointestinal tract in critical illness: nutritional implications. Curr Opin Clin Nutr Metab Care 6(5): 587-591. doi: 10.1097/01.mco.0000087973.83880.9a
12. Stechmiller JK et al (1997) Gut dysfunction in critically ill patients: a review of the literature. Am J Crit Care 6(3): 204-209.
13. * Swank GM, Deitch EA (1996) Role of the gut in multiple organ failure: bacterial translocation and permeability changes. World J Surg 20(4): 411-417.
14. Wang HW et al (2016) Indications, techniques, and clinical outcomes of thoracic duct interventions in patients: a forgotten literature? J Surg Res 204(1): 213-227. doi: 10.1016/j.jss.2016.04.050

Other

1. Deitch EA et al (2014) Trauma-hemorrhagic shock induces a CD36-dependent RBC endothelial-adhesive phenotype. Crit Care Med 42(3): e200-210. doi: 10.1097/CCM.0000000000000119
2. Meier JJ (2010) Waking up the gut in critically ill patients. Crit Care 14(5): 183. doi: 10.1186/cc9079

Additional papers identified (14):

RCT

1. Harvey SE et al (2016) A multicentre, randomised controlled trial comparing the clinical effectiveness and cost-effectiveness of early nutritional support via the parenteral versus the enteral route in critically ill patients (CALORIES). Health Technol Assess. 2016;20:1-144. doi: 10.3310/hta20280
2. Reignier J et al (2018) Enteral versus parenteral early nutrition in ventilated adults with shock: a randomised, controlled, multicentre, open-label, parallel-group study (NUTRIREA-2). Lancet 391(10116):133-143. doi: 10.1016/S0140-6736(17)32146-3

Observational studies

1. Habes QLM et al (2018) Norepinephrine contributes to enterocyte damage in septic shock patients ; a prospective cohort study. Shock 49:137-143. doi: 10.1097/SHK.0000000000000955
2. * Dickson RP et al (2016) Enrichment of the lung microbiome with gut bacteria in sepsis and the acute respiratory distress syndrome. Nat Microbiol 1(10):16113. doi: 10.1038/nmicrobiol.2016.113. *Combined with an experimental study.*

Case series

1. Versyck G et al (2017) Non-occlusive mesenteric ischemia: two case reports and a short review of the literature. Acta Chir Belg 25:1-6. doi: 10.1080/00015458.2017.1408280

Reviews

1. Carrico CJ et al (1986) Multiple-organ-failure syndrome. Arch Surg 121:196-208
2. * de Jong PR et al (2016) The digestive tract as the origin of systemic inflammation. Crit Care 20:279. doi: 10.1001/archsurg.1986.01400020082010
3. * Haak BW, Wiersinga WJ (2017) The role of the gut microbiota in sepsis. Lancet Gastroenterol Hepatol 2:135-143. doi: 10.1016/S2468-1253(16)30119-4
4. Klek S et al (2016) Management of acute intestinal failure: A position paper from the European Society for Clinical Nutrition and Metabolism (ESPEN) Special Interest Group. Clin Nutr 35:1209-1218. doi: 10.1016/j.clnu.2016.04.009
5. Kolkman JJ, Mensink PB (2003) Non-occlusive mesenteric ischaemia: a common disorder in gastroenterology and intensive care. Best Pract Res Clin Gastroenterol 17:457-473.
6. Sertaridou E et al (2015) Gut failure in critical care: old school versus new school. Ann Gastroenterol 28:309-322

Other

1. Andrade-Oliveira V et al (2015) Gut Bacteria Products Prevent AKI Induced by Ischemia-Reperfusion. J Am Soc Nephrol 26:1877-1888. doi: 10.1681/ASN.2014030288
2. Chen G, Huang B, Fu S, Li B, Ran X, He D, Jiang L, Li Y, Liu B, Xie L, Liu J, Wang W (2018) G Protein-Coupled Receptor 109A and Host Microbiota Modulate Intestinal Epithelial Integrity During Sepsis. Front Immunol 9:2079. doi: 10.3389/fimmu.2018.02079
3. Deitch EA et al (2001) A time course study of the protective effect of mesenteric lymph duct ligation on hemorrhagic shock-induced pulmonary injury and the toxic effects of lymph from shocked rats on endothelial cell monolayer permeability. Surgery 129:39-47

**Topic 13 Microbiome**

RCT = 0

Observational = 6

Review = 10

Case series = 0

Other = 7

Papers identified from electronic databases (5):

Reviews

1. McClave SA, et al (2018) Should fecal microbial transplantation be used in the ICU? Curr Opin Crit Care 24:105-111. doi: 10.1097/MCC.0000000000000489

Other

1. * Bai J et al (2018) The gut microbiome, symptoms, and targeted interventions in children with cancer: a systematic review. Support Care Cancer 26(2): 427-439. doi: 10.1007/s00520-017-3982-3
2. * Costeloe K et al (2016) A randomised controlled trial of the probiotic Bifidobacterium breve BBG-001 in preterm babies to prevent sepsis, necrotising enterocolitis and death: the Probiotics in Preterm infantS (PiPS) trial. Health Technol Assess 20(66):1-194. doi: 10.3310/hta20660
3. * Sherman MP et al (2016) Randomized Control Trial of Human Recombinant Lactoferrin: a Substudy Reveals Effects on the Fecal Microbiome of Very Low Birth Weight Infants. J Pediatr 173 Suppl: S37‐42. doi: 10.1016/j.jpeds.2016.02.074
4. * Zaborin A et al (2014) Phosphate-containing polyethylene glycol polymers prevent lethal sepsis by multidrug-resistant pathogens. Antimicrob Agents Chemother 58(2): 966-977. doi: 10.1128/AAC.02183-13

Additional papers identified (18):

Observational studies

1. * Buelow E et al (2017) Comparative gut microbiota and resistome profiling of intensive care patients receiving selective digestive tract decontamination and healthy subjects. Microbiome 5(1):88. doi: 10.1186/s40168-017-0309-z
2. * Freedberg DE et al (2018) Pathogen colonization of the gastrointestinal microbiome at intensive care unit admission and risk for subsequent death or infection. Intensive Care Med 44(8):1203-1211. doi: 10.1007/s00134-018-5268-8
3. * Howard BM et al (2017) Characterizing the gut microbiome in trauma: significant changes in microbial diversity occur early after severe injury. Trauma Surg Acute Care Open 2(1):e000108. doi: 10.1136/tsaco-2017-000108
4. * Lankelma JM et al. (2017) Critically ill patients demonstrate large interpersonal variation in intestinal microbiota dysregulation: a pilot study. Intensive Care Med 43:59-68. doi: 10.1007/s00134-016-4613-z
5. * McDonald D et al (2016) Extreme Dysbiosis of the Microbiome in Critical Illness. mSphere 1(4). pii: e00199-16. doi: 10.1128/mSphere.00199-16
6. * Yeh A et al (2016) Dysbiosis Across Multiple Body Sites in Critically Ill Adult Surgical Patients. Shock 46(6):649-654. doi: 10.1097/SHK.0000000000000691

Reviews

1. * Akrami K, Sweeney DA (2018) The microbiome of the critically ill patient. Curr Opin Crit Care 24:49-54. doi: 10.1097/MCC.0000000000000469
2. * Alverdy JC, Krezalek MA (2017) Collapse of the Microbiome, Emergence of the Pathobiome, and the Immunopathology of Sepsis. Crit Care Med 45:337-347. doi: 10.1097/CCM.0000000000002172
3. * Alverdy JC, Luo JN (2017) The Influence of Host Stress on the Mechanism of Infection: Lost Microbiomes, Emergent Pathobiomes, and the Role of Interkingdom Signaling. Front Microbiol 8:322. doi: 10.3389/fmicb.2017.00322
4. * Kitsios GD et al (2017) Dysbiosis in the intensive care unit: Microbiome science coming to the bedside. J Crit Care 38:84-91. doi: 10.1016/j.jcrc.2016.09.029
5. * Krezalek MA et al (2016) The Shift of an Intestinal “Microbiome” to a “Pathobiome” Governs the Course and Outcome of Sepsis Following Surgical Injury. Shock 45:475-482. doi: 10.1097/SHK.0000000000000534
6. * Le Bastard Q et al (2018) Systematic review: human gut dysbiosis induced by non-antibiotic prescription medications. Aliment Pharmacol Ther 47:332-345. doi: 10.1111/apt.14451
7. * Mittal R, Coopersmith CM (2014) Redefining the gut as the motor of critical illness. Trends Mol Med 20:214-223. doi: 10.1016/j.molmed.2013.08.004
8. * Wiersinga WJ (2017) The gut microbiome takes center stage in critical care. Curr Opin Crit Care 23:140-142. doi: 10.1097/MCC.0000000000000390
9. * Wolff NS et al (2018) The emerging role of the microbiota in the ICU. Crit Care 22:78. doi: 10.1186/s13054-018-1999-8

Other

1. * Chen G et al (2018) G Protein-Coupled Receptor 109A and Host Microbiota Modulate Intestinal Epithelial Integrity During Sepsis. Front Immunol 9:2079. doi: 10.3389/fimmu.2018.02079
2. * Fox AC et al (2012) The endogenous bacteria alter gut epithelial apoptosis and decrease mortality following Pseudomonas aeruginosa pneumonia. Shock 38:508-514. doi: 10.1097/SHK.0b013e31826e47e8
3. * Iapichino G et al (2017) Gut microbiota disruption in critically ill patients: Discussion on “Critically ill patients demonstrate large interpersonal variation of intestinal microbiota dysregulation: a pilot study. Intensive Care Med; 43:718-719. doi: 10.1007/s00134-017-4713-4

**Topic 14 Bacterial translocation/mucosal integrity**

RCT = 9

Observational = 31

Review = 14

Case series = 4

Other = 24

Papers identified from electronic databases (33):

RCTs

1. Brinkmann A et al (1996) Perioperative endotoxemia and bacterial translocation during major abdominal surgery: evidence for the protective effect of endogenous prostacyclin? Crit Care Med 24(8): 1293-1301. doi: 10.1097/00003246-199608000-00005
2. * Hadfield RJ et al (1995) Effects of enteral and parenteral nutrition on gut mucosal permeability in the critically ill. Am J Respir Crit Care Med 152(5 Pt 1): 1545-1548. doi: 10.1164/ajrccm.152.5.7582291
3. Jain PK et al (2004) Influence of synbiotic containing Lactobacillus acidophilus La5, Bifidobacterium lactis Bb 12, Streptococcus thermophilus, Lactobacillus bulgaricus and oligofructose on gut barrier function and sepsis in critically ill patients: a randomised controlled trial. Clin Nutr 23(4):467-75. 10.1016/j.clnu.2003.12.002
4. * Nguyen NQ et al (2012) Delayed enteral feeding impairs intestinal carbohydrate absorption in critically ill patients. Crit Care Med 40(1): 50-54. doi: 10.1097/CCM.0b013e31822d71a6
5. Shariatpanahi ZV et al (2019) Effects of Early Enteral Glutamine Supplementation on Intestinal Permeability in Critically Ill Patients. Indian J Crit Care Med 23(8):356-362. doi: 10.5005/jp-journals-10071-23218
6. Stadlbauer V et al (2019) Dysbiosis in early sepsis can be modulated by a multispecies probiotic: a randomised controlled pilot trial. Benef Microbes 10(3):265-278. doi: 10.3920/BM2018.0067

Observational studies

1. * Burgstad CM et al (2013) Sucrose malabsorption and impaired mucosal integrity in enterally fed critically ill patients: a prospective cohort observational study. Crit Care Med 41(5): 1221-1228. doi: 10.1097/CCM.0b013e31827ca2fa
2. Buttenschoen K et al (1996) Plasma concentrations of endotoxin and antiendotoxin antibodies in patients with multiple injuries: a prospective clinical study. Eur J Surg 162(11): 853-860.
3. Charbonney E et al (2016) Endotoxemia Following Multiple Trauma: Risk Factors and Prognostic Implications. Crit Care Med 44(2): 335-341. doi: 10.1097/CCM.0000000000001404
4. * Doig CJ et al (1998) Increased intestinal permeability is associated with the development of multiple organ dysfunction syndrome in critically ill ICU patients. Am J Respir Crit Care Med 158(2): 444-451. 10.1164/ajrccm.158.2.9710092
5. Kim OY et al (2009) Translocation of bacterial NOD2 agonist and its link with inflammation. Crit Care 13(4): R124. doi: 10.1186/cc7980
6. Oudemans-van Straaten HM et al (2002) Pitfalls in gastrointestinal permeability measurement in ICU patients with multiple organ failure using differential sugar absorption. Intensive Care Med 28(2): 130-138. doi: 10.1007/s00134-001-1140-2
7. Sekino M et al (2019) Association between endotoxemia and enterocyte injury and clinical course in patients with gram-positive septic shock: A posthoc analysis of a prospective observational study. Medicine (Baltimore) 98(28):e16452. doi: 10.1097/MD.0000000000016452
8. Sinclair DG et al (1995) The effect of cardiopulmonary bypass on intestinal and pulmonary endothelial permeability. Chest 108(3): 718-724. doi: 10.1378/chest.108.3.718

Reviews

1. * Assimakopoulos SF et al (2018) Gut-origin sepsis in the critically ill patient: pathophysiology and treatment. Infection 46:751-760. doi: 10.1007/s15010-018-1178-5
2. Deitch EA (1993) Nutrition and the gut mucosal barrier. Curr Opin Gen Surg. 1993:85-91. doi: 10.1079/pns2001103
3. De-Souza DA, Greene LJ (2005) Intestinal permeability and systemic infections in critically ill patients: effect of glutamine. Crit Care Med 33(5): 1125-1135. doi: 10.1097/01.ccm.0000162680.52397.97
4. Jeejeebhoy KN (2001) Enteral and parenteral nutrition: evidence-based approach. Proc Nutr Soc 60(3):399-402
5. Lipman TO (1998) Grains or veins: is enteral nutrition really better than parenteral nutrition? A look at the evidence. JPEN J Parenter Enteral Nutr 22(3):167-82. doi: 10.1177/0148607198022003167
6. Mu J et al (2019) Influence of gut microbiota and intestinal barrier on enterogenic infection after liver transplantation. Curr Med Res Opin 35(2):241-248. doi: 10.1080/03007995.2018.1470085
7. * Vermette D et al (2018) Tight junction structure, function, and assessment in the critically ill: a systematic review. Intensive Care Med Exp 6(1): 37. doi: 10.1186/s40635-018-0203-4

Other

1. Diepenhorst GM et al (2011) Influence of prophylactic probiotics and selective decontamination on bacterial translocation in patients undergoing pancreatic surgery: a randomized controlled trial. Shock 35(1):9-16. doi: 10.1097/SHK.0b013e3181ed8f17.
2. Hu J et al (2019) Regulatory T Cells Could Improve Intestinal Barrier Dysfunction in Heatstroke. Inflammation 42(4):1228-1238. doi: 10.1007/s10753-019-00983-6
3. Ikeda M et al (2018) Hydrogen-Rich Saline Regulates Intestinal Barrier Dysfunction, Dysbiosis, and Bacterial Translocation in a Murine Model of Sepsis. Shock 50(6):640-647. doi: 10.1097/SHK.0000000000001098.
4. Kelly P et al (2010) Gastric and intestinal barrier impairment in tropical enteropathy and HIV: limited impact of micronutrient supplementation during a randomised controlled trial. BMC Gastroenterol 10:72. doi: 10.1186/1471-230X-10-72
5. Lages PC et al (2018) Postoperative symbiotic in patients with head and neck cancer: a double-blind randomised trial. Br J Nutr 119(2):190-195. doi: 10.1017/S0007114517003403
6. Lanyero B et al (2019) Correlates of Gut Function in Children Hospitalized for Severe Acute Malnutrition, a Cross-sectional Study in Uganda. J Pediatr Gastroenterol Nutr 69(3):292-298. doi: 10.1097/MPG.0000000000002381
7. March DS et al (2019) The effect of bovine colostrum supplementation on intestinal injury and circulating intestinal bacterial DNA following exercise in the heat. Eur J Nutr 58(4):1441-1451. doi: 10.1007/s00394-018-1670-9
8. O’Dwyer ST et al (1988) A single dose of endotoxin increases intestinal permeability in healthy humans. Arch Surg 123(12): 1459-1464. doi: 10.1001/archsurg.1988.01400360029003
9. Sato J et al (2017) Probiotic reduces bacterial translocation in type 2 diabetes mellitus: A randomised controlled study. Sci Rep 7(1):12115. doi: 10.1038/s41598-017-12535-9.
10. Sinkala E et al (2018) Rifaximin Reduces Markers of Inflammation and Bacterial 16S rRNA in Zambian Adults with Hepatosplenic Schistosomiasis: A Randomized Control Trial. Am J Trop Med Hyg 98(4):1152-1158. doi: 10.4269/ajtmh.17-0637
11. Wen ZL et al (2019) Effect of broad-spectrum antibiotics on bacterial translocation in burned or septic rats. Chin Med J (Engl) 132(10):1179-1187. doi: 10.1097/CM9.0000000000000242.

Additional papers identified (49):

RCTs

1. Besselink MG et al (2009) Intestinal barrier dysfunction in a randomized trial of a specific probiotic composition in acute pancreatitis. Ann Surg 250(5):712-9. doi: 10.1097/SLA.0b013e3181bce5bd
2. Mangell P et al (2012) Lactobacillus plantarum 299v does not reduce enteric bacteria or bacterial translocation in patients undergoing colon resection. Dig Dis Sci 57: 1915-1924. doi: 10.1007/s10620-012-2102-y

Observational studies

1. Deitch EA (1990) Intestinal permeability is increased in burn patients shortly after injury. Surgery 107: 411-6
2. Deitch EA (1989) Simple intestinal obstruction causes bacterial translocation in man. Arch Surg 124: 699-701. doi: 10.1001/archsurg.1989.01410060065013
3. Derikx JPM et al (2007) Evidence for intestinal and liver epithelial cell injury in the early phase of sepsis. Shock 28:544–548. doi: 10.1097/shk.0b013e3180644e32
4. Greis C et al (2017) Intestinal T lymphocyte homing is associated with gastric emptying and epithelial barrier function in critically ill: a prospective observational study. Crit Care 21(1):70. doi: 10.1186/s13054-017-1654-9
5. Guidet B et al (1994) Endotoxemia and bacteremia in patients with sepsis syndrome in the intensive care unit. Chest 106: 1194-1201. 10.1378/chest.106.4.1194
6. Harris CE et al (1992) Intestinal permeability in the critically ill. Intensive Care Med 18(1):38-41.
7. * Holland J et al (2005) Intraoperative splanchnic hypoperfusion, increased intestinal permeability, down-regulation of monocyte class II major histocompatibility complex expression, exaggerated acute phase response, and sepsis. Am J Surg 190(3): 393-400. doi: 10.1016/j.amjsurg.2005.03.038
8. * Li H et al (2017) Association between acute gastrointestinal injury and biomarkers of intestinal barrier function in critically ill patients. BMC Gastroenterol 17(1): 45. doi: 10.1186/s12876-017-0603-z
9. * MacFie J et al (1999) Gut origin of sepsis: a prospective study investigating association between bacterial translocation, gastric microflora, and septic morbidity. Gut 45:223–228. doi: 10.1136/gut.45.2.223
10. * Marshall JC et al (2004) Diagnostic and prognostic implications of endotoxemia in critical illness: results of MEDIC study. J Infect Dis 190(3):527-534. doi: 10.1086/422254
11. * Mizuno T et al (2010) Intraoperative bacterial translocation detected by bacterium-specific ribosomal rna-targeted reverse-transcriptase polymerase chain reaction for the mesenteric lymph node strongly predicts postoperative infectious complications after major hepatectomy for biliary malignancies. Ann Surg 252(6):1013-1019. doi: 10.1097/SLA.0b013e3181f3f355
12. Moore FA et al (1991) Gut bacterial translocation via the portal vein: a clinical perspective with major torso trauma. J Trauma 31: 629-636
13. Moore FA et al (1992) Postinjury shock and early bacteremia. A lethal combination. Arch Surg 127: 893-897. doi: 10.1001/archsurg.1992.01420080027004
14. * O’Boyle CJ et al (1998) Microbiology of bacterial translocation in humans. Gut 42:29–35. doi: 10.1136/gut.42.1.29
15. Ono S et al (2005) Detection of microbial DNA in the blood of surgical patients for diagnosing bacterial translocation. World J Surg 29: 535-539. doi: 10.1007/s00268-004-7618-7
16. Pijls KE et al (2014) Large intestine permeability is increased in patients with compensated liver cirrhosis. Am J Physiol Gastrointest Liver Physiol 306(2):G147-153. doi: 10.1152/ajpgi.00330.2013
17. Peitzman AB et al (1991) Bacterial translocation in trauma patients. J Trauma 31: 1083-1086
18. * Piton G et al (2010) Plasma citrulline kinetics and prognostic value in critically ill patients. Intensive Care Med 36:702–706. doi: 10.1007/s00134-010-1751-6
19. * Piton G et al (2013) Enterocyte damage in critically ill patients is associated with shock condition and 28-day mortality. Crit Care Med 41(9):2169-2176. doi: 10.1097/CCM.0b013e31828c26b5.
20. Reddy BS et al (2006) Surgical manipulation of the large intestine increases bacterial translocation in patients undergoing elective colorectal surgery. Colorectal Dis 8: 596-600. doi: 10.1111/j.1463-1318.2006.01024.x
21. Roumen RM et al (1993) Intestinal permeability after severe trauma and hemorrhagic shock is increased without relation to septic complications. Arch Surg 128: 453-457. doi: 10.1001/archsurg.1993.01420160095016
22. * Rush BF Jr et al (1988) Endotoxemia and bacteremia during hemorrhagic shock. The link between trauma and sepsis? Ann Surg 207: 549-554. 10.1097/00000658-198805000-00009
23. Hotchkiss RS et al (1999) Apoptotic cell death in patients with sepsis, shock, and multiple organ dysfunction. Crit Care Med 27(7):1230-1251. doi: 10.1097/00003246-199907000-00002

Case series

1. Hernandez G et al (2007) Splanchnic ischemia and gut permeability after acute brain injury secondary to intracranial hemorrhage. Neurocrit Care 7: 40-44. doi: 10.1007/s12028-007-0026-8
2. Lemaire LC et al (1999) Thoracic duct in patients with multiple organ failure: no major route of bacterial translocation. Ann Surg 229: 128-136. 10.1097/00000658-199901000-00017
3. * MacFie J et al (2006) Bacterial translocation studied in 927 patients over 13 years. Br J Surg 93(1):87-93. doi: 10.1002/bjs.5184
4. Sedman PC et al (1994) The prevalence of gut translocation in humans. Gastroenterology 107: 643-649. 10.1016/0016-5085(94)90110-4

Reviews

1. Berg RD (1999) Bacterial translocation from the gastrointestinal tract. Adv Exp Med Biol 473: 11-30. 10.1007/978-1-4615-4143-1_2
2. * Fink MP (1994) Effect of critical illness on microbial translocation and gastrointestinal mucosa permeability. Semin Respir Infect 9(4): 256-260.
3. Klingensmith NJ, Coopersmith CM (2016) The Gut as the Motor of Multiple Organ Dysfunction in Critical Illness. Crit Care Clin 32(2):203-12. doi: 10.1016/j.ccc.2015.11.004
4. * Meng M et al (2017) New insights into the gut as the driver of critical illness and organ failure. Curr Opin Crit Care 23:143-148. doi: 10.1097/MCC.0000000000000386
5. Peterson LW, Artis D (2014) Intestinal epithelial cells: regulators of barrier function and immune homeostasis. Nat Rev Immunol 14(3):141-153. doi: 10.1038/nri3608.
6. Tsujimoto H et al (2009) Role of translocation of pathogen-associated molecular patterns in sepsis. Dig Surg 26: 100-109. doi: 10.1159/000206143
7. * Wen Z et al (2019) A Protective Role of the NRF2-Keap1 Pathway in Maintaining Intestinal Barrier Function. Oxid Med Cell Longev 2019:1759149. doi: 10.1155/2019/1759149

Other

1. * Armacki M et al (2018) Thirty-eight-negative kinase 1 mediates trauma-induced intestinal injury and multi-organ failure. J Clin Invest 128(11):5056-5072. doi: 10.1172/JCI97912
2. * Chen G et al (2018) G Protein-Coupled Receptor 109A and Host Microbiota Modulate Intestinal Epithelial Integrity During Sepsis. Front Immunol 9:2079. doi: 10.3389/fimmu.2018.02079
3. Demaude J et al (2006) Phenotypic changes in colonocytes following acute stress or activation of mast cells in mice: implications for delayed epithelial barrier dysfunction. Gut 55: 655-661. doi: 10.1136/gut.2005.078675
4. Derikx JP et al (2008) New Insight in Loss of Gut Barrier during Major Non-Abdominal Surgery. PLoS.One 3: e3954. doi: 10.1371/journal.pone.0003954
5. Fazal N et al (2000) Neutrophil depletion in rats reduces burn-injury induced intestinal bacterial translocation. Crit Care Med 28(5):1550-5.
6. Han X et al (2004) Increased iNOS activity is essential for intestinal epithelial tight junction dysfunction in endotoxemic mice. Shock 21(3): 261-270. doi: 10.1097/01.shk.0000112346.38599.10
7. Hietbrink F et al (2009) Systemic inflammation increases intestinal permeability during experimental human endotoxemia. Shock 32: 374-378. doi: 10.1097/SHK.0b013e3181a2bcd6
8. Kramski M et al (2011) Novel sensitive real-time PCR for quantification of bacterial 16S rRNA genes in plasma of HIV-infected patients as a marker for microbial translocation. J.Clin.Microbiol 49: 3691-3693. doi: 10.1128/JCM.01018-11
9. Marchbank T et al (2011) The nutriceutical bovine colostrum truncates the increase in gut permeability caused by heavy exercise in athletes. Am.J.Physiol Gastrointest.Liver Physiol 300: G477-G484. doi: 10.1152/ajpgi.00281.2010
10. Rentea RM et al (2018) Role of intestinal Hsp70 in barrier maintenance: contribution of milk to the induction of Hsp70.2. Pediatr Surg Int 34(3): 323-330. doi: 10.1007/s00383-017-4211-3
11. Sandler NG et al (2011) Plasma levels of soluble CD14 independently predict mortality in HIV infection. J Infect Dis 203(6):780-90. doi: 10.1093/infdis/jiq118
12. Subbarao S et al (2015) Raised Venous Lactate and Markers of Intestinal Translocation Are Associated With Mortality Among In-Patients With HIV-Associated TB in Rural South Africa. J Acquir Immune Defic Syndr 70(4):406-13. doi: 10.1097/QAI.0000000000000763
13. * Wen ZS et al (2019) Low Molecular Seleno-Aminopolysaccharides Protect the Intestinal Mucosal Barrier of Rats under Weaning Stress. Int J Mol Sci 20(22). pii: E5727. doi: 10.3390/ijms20225727

**Topic 15 GI hormones**

RCT = 14

Observational = 10

Review = 10

Case series = 1

Other = 1

Papers identified from electronic databases (4):

Observational

1. * Crona D, MacLaren R (2012) Gastrointestinal hormone concentrations associated with gastric feeding in critically ill patients. JPEN J Parenter Enteral Nutr 36(2): 189-196. doi: 10.1177/0148607111413770

Reviews

1. * Deane A et al (2010) Bench-to-bedside review: the gut as an endocrine organ in the critically ill. Crit Care 14(5): 228. doi: 10.1186/cc9039
2. * Khoo J et al (2010). Gastrointestinal hormonal dysfunction in gastroparesis and functional dyspepsia. Neurogastroenterol Motil 22(12): 1270-1278. doi: 10.1111/j.1365-2982.2010.01609.x

Other

1. Martinez EE et al. Gastrointestinal Hormone Profiles Associated With Enteral Nutrition Tolerance and Gastric Emptying in Pediatric Critical Illness: A Pilot Study. JPEN J Parenter Enteral Nutr. 2019 Jul 15. doi: 10.1002/jpen.1678. [Epub ahead of print]

Additional papers identified (32):

RCTs

1. Chapman MJ et al (2016) The effect of camicinal (GSK962040), a motilin agonist, on gastric emptying and glucose absorption in feed-intolerant critically ill patients: a randomized, blinded, placebo-controlled, clinical trial. Crit Care 20(1):232. doi: 10.1186/s13054-016-1420-4
2. Deane AM et al (2009) The effect of exogenous glucagon-like peptide-1 on the glycaemic response to small intestinal nutrient in the critically ill: a randomised double-blind placebo controlled cross over study. Crit Care 13(3):R67. doi: 10.1186/cc7874
3. Deane AM et al (2010) Effects of exogenous glucagon-like peptide-1 on gastric emptying and glucose absorption in the critically ill: Relationship to glycemia. Crit Care Med 38(5):1261-1269. doi: 10.1097/CCM.0b013e3181d9d87a
4. Deane AM et al (2017) Nutrition Adequacy Therapeutic Enhancement in the Critically Ill: A Randomized Double-Blind, Placebo-Controlled Trial of the Motilin Receptor Agonist Camicinal (GSK962040): The NUTRIATE Study. JPEN J Parenter Enteral Nutr 42(5):949-959. doi: 10.1002/jpen.1038
5. Galiatsatos P et al (2014) The glucoregulatory benefits of glucagon-like peptide-1 (7-36) amide infusion during intensive insulin therapy in critically ill surgical patients: a pilot study. Crit Care Med 42(3):638-645. doi: 10.1097/CCM.0000000000000035
6. Hayakawa M et al (2014) Effects of Rikkunshito (traditional Japanese medicine) on enteral feeding and the plasma ghrelin level in critically ill patients: a pilot study. J Intensive Care 2(1):53. doi: 10.1186/s40560-014-0053-4
7. Kar P et al (2015) Effects of glucose-dependent insulinotropic polypeptide on gastric emptying, glycaemia and insulinaemia during critical illness: a prospective, double blind, randomised, crossover study. Crit Care 19:20. doi: 10.1186/s13054-014-0718-3
8. Kohl BA et al (2014) Intravenous GLP-1 (7-36) amide for prevention of hyperglycemia during cardiac surgery: a randomized, double-blind, placebo-controlled study. J Cardiothorac Vasc Anesth 28(3):618-25. doi: 10.1053/j.jvca.2013.06.021
9. Miller A et al (2017) Exogenous glucagon-like peptide-1 attenuates glucose absorption and reduces blood glucose concentration after small intestinal glucose delivery in critical illness. Crit Care Resusc 19(1):37-42.
10. * Nguyen NQ et al (2008) The impact of delaying enteral feeding on gastric emptying, plasma cholecystokinin, and peptide YY concentrations in critically ill patients. Crit Care Med 36(5):1469-1474. doi: 10.1097/CCM.0b013e31816fc457
11. Nguyen NQ et al (2007) Erythromycin is more effective than metoclopramide in the treatment of feed intolerance in critical illness. Crit Care Med 35(2):483-489. 10.1097/01.CCM.0000253410.36492.E9
12. MacLaren R et al (2008) Erythromycin vs metoclopramide for facilitating gastric emptying and tolerance to intragastric nutrition in critically ill patients. JPEN J Parenter Enteral Nutr 32(4):412-419. doi: 10.1177/0148607108319803
13. Temel JS et al (2016) Anamorelin in patients with non-small-cell lung cancer and cachexia (ROMANA 1 and ROMANA 2): results from two randomised, double-blind, phase 3 trials. Lancet Oncol 17(4):519-531. doi: 10.1016/S1470-2045(15)00558-6
14. Wiberg S et al (2016) Neuroprotective Effects of the Glucagon-Like Peptide-1 Analog Exenatide After Out-of-Hospital Cardiac Arrest: A Randomized Controlled Trial. Circulation 134(25):2115-2124. 10.1161/CIRCULATIONAHA.116.024088

Observational studies

1. * Lebherz C et al (2017) GLP-1 Levels Predict Mortality in Patients with Critical Illness as Well as End-Stage Renal Disease. Am J Med 130(7):833-841.e3. doi: 10.1016/j.amjmed.2017.03.010
2. Llompart-Pou JA et al (2012) Stress hyperglycaemia in critically ill patients: potential role of incretin hormones; a preliminary study. Nutr Hosp 27(1):130-137. doi: 10.1590/S0212-16112012000100015
3. * Nematy M et al (2006) Changes in appetite related gut hormones in intensive care unit patients: a pilot cohort study. Crit Care 10(1):R10. doi: 10.1186/cc3957
4. Nguyen NQ et al (2007) The relationship between gastric emptying, plasma cholecystokinin, and peptide YY in critically ill patients. Crit Care 11(6):R132. doi: 10.1186/cc6205
5. * Nguyen NQ et al (2006) Fasting and nutrient-stimulated plasma peptide-YY levels are elevated in critical illness and associated with feed intolerance: an observational, controlled study. Crit Care 10(6):R175. 10.1186/cc5127
6. * Nguyen NQ et al (2007) Feed intolerance in critical illness is associated with increased basal and nutrient-stimulated plasma cholecystokinin concentrations. Crit Care Med 35(1):82-88. doi: 10.1097/01.CCM.0000250317.10791.6C
7. Plummer MP et al (2016) Critical Illness Is Associated With Impaired Gallbladder Emptying as Assessed by 3D Ultrasound. Crit Care Med 44(9):e790-796. doi: 10.1097/CCM.0000000000001715
8. * Santacruz CA et al (2017) Is There a Role for Enterohormones in the Gastroparesis of Critically Ill Patients? Crit Care Med 45(10):1696-1701. doi: 10.1097/CCM.0000000000002625
9. * Summers MJ et al (2014) Endogenous amylin and glucagon-like peptide-1 concentrations are not associated with gastric emptying in critical illness. Acta Anaesthesiol Scand 58(2):235-242. doi: 10.1111/aas.12252

Reviews

1. * Chapman MJ et al (2013) Gastrointestinal dysmotility: evidence and clinical management. Curr Opin Clin Nutr Metab Care 16(2):209-216. doi: 10.1097/MCO.0b013e32835c1fa5
2. * Deane AM, Jeppesen PB (2014) Understanding incretins. Intensive Care Med 40(11):1751-1754. doi: 10.1007/s00134-014-3435-0
3. * Hill NE et al (2012) Ghrelin, appetite and critical illness. Curr Opin Crit Care 18(2):199-205. doi: 10.1097/MCC.0b013e3283514b01
4. * Hulst AH et al (2018) Systematic review of incretin therapy during peri-operative and intensive care. Crit Care 22(1):299. doi: 10.1186/s13054-018-2197-4.
5. * Luttikhold J et al (2013) Review article: the role of gastrointestinal hormones in the treatment of delayed gastric emptying in critically ill patients. Aliment Pharmacol Ther 38(6):573-583. doi: 10.1111/apt.12421
6. * Narula T, deBoisblanc BP (2015) Ghrelin in Critical Illness. Am J Respir Cell Mol Biol 53(4):437-442. doi: 10.1165/rcmb.2014-0226TR
7. * Olariu E et al (2018) A systematic scoping review on the consequences of stress-related hyperglycaemia. PLoS One 13(4):e0194952. doi: 10.1371/journal.pone.0194952
8. * Plummer MP et al (2014) Incretins and the intensivist: what are they and what does an intensivist need to know about them? Crit Care 18(2):205. doi: 10.1186/cc13737

Case series

1. Nielsen ST et al (2015) The incretin effect in critically ill patients: a case-control study. Crit Care 19:402. doi: 10.1186/s13054-015-1118-z

**Topic 16 Bile acids**

RCT = 0

Observational = 4

Review = 12

Case series = 0

Other = 10

Papers identified from electronic databases (6):

Reviews

1. * Cheung A, Flamm S (2019) Hepatobiliary Complications in Critically Ill Patients. Clin Liver Dis 23(2):221-232. doi: 10.1016/j.cld.2018.12.005.
2. * Jenniskens M et al (2016) Cholestatic liver (dys)function during sepsis and other critical illnesses. Intensive Care Med 42(1):16-27. doi: 10.1007/s00134-015-4054-0
3. * Theiler-Schwetz V et al (2019) Bile acids and glucocorticoid metabolism in health and disease. Biochim Biophys Acta Mol Basis Dis.1865(1):243-251. doi: 10.1016/j.bbadis.2018.08.001.

Other

1. Claus SP et al (2011) Colonization-induced host-gut microbial metabolic interaction. MBio 2(2): e00271-00210. doi: 10.1128/mBio.00271-10
2. * Vanwijngaerden YM et al (2014) Impact of parenteral nutrition versus fasting on hepatic bile acid production and transport in a rabbit model of prolonged critical illness. Shock 41(1):48-54. doi: 10.1097/SHK.0000000000000046
3. Wijeyesekera A et al (2019) Multi-Compartment Profiling of Bacterial and Host Metabolites Identifies Intestinal Dysbiosis and Its Functional Consequences in the Critically Ill Child. Crit Care Med. 2019 Sep;47(9):e727-e734. doi: 10.1097/CCM.0000000000003841.

Additional papers identified (20):

Observational studies

1. Fuhrmann V et al (2011) Impact of hypoxic hepatitis on mortality in the intensive care unit. Intensive Care Med 37:1302-1310. doi: 10.1007/s00134-011-2248-7
2. * Horvatits T et al (2017) Circulating bile acids predict outcome in critically ill patients. Ann Intensive Care 7:48. doi: 10.1186/s13613-017-0272-7
3. * Horvatits T et al (2017) Serum bile acids as marker for acute decompensation and acute-on-chronic liver failure in patients with non-cholestatic cirrhosis. Liver Int 37:224-231. doi: 10.1111/liv.13201
4. Jäger B et al (2012) Jaundice increases the rate of complications and one-year mortality in patients with hypoxic hepatitis. Hepatology 56:2297-2304. doi: 10.1002/hep.25896

Reviews

1. Cai JS, Chen JH (2014) The mechanism of enterohepatic circulation in the formation of gallstone disease. J Membr Biol 247(11):1067-82. doi: 10.1007/s00232-014-9715-3
2. European Association for the Study of the Liver. Electronic address eee, Clinical practice guidelines panel, Wendon J et al (2017) EASL Clinical Practical Guidelines on the management of acute (fulminant) liver failure. J Hepatol 66:1047-1081. doi: 10.1016/j.jhep.2016.12.003
3. Fuhrmann V et al (2018) The ten tips to manage critically ill patients with acute-on-chronic liver failure. doi: 10.1007/s00134-018-5078-z
4. Horvatits T et al (2013) Hypoxic liver injury and cholestasis in critically ill patients. Curr Opin Crit Care 19:128-132. doi: 10.1097/MCC.0b013e32835ec9e6
5. * Jenniskens M et al (2018) Cholestatic Alterations in the Critically Ill: Some New Light on an Old Problem. Chest 153:733-743. doi: 10.1016/j.chest.2017.08.018
6. Keating N, Keely SJ (2009) Bile acids in regulation of intestinal physiology. Curr Gastroenterol Rep 11(5):375-382.
7. Li T, Chiang JY (2015) Bile acids as metabolic regulators. Curr Opin Gastroenterol 31:159-165. doi: 10.1097/MOG.0000000000000156
8. Manley S, Ding W (2015) Role of farnesoid X receptor and bile acids in alcoholic liver disease. Acta Pharm Sin B 5:158-167. doi: 10.1016/j.apsb.2014.12.011
9. Ridlon JM et al (2013) Cirrhosis, bile acids and gut microbiota: unraveling a complex relationship. Gut Microbes 4:382-387. doi: 10.4161/gmic.25723

Other

1. Feingold K et al (2004) Altered expression of nuclear hormone receptors and coactivators in mouse heart during the acute-phase response. Am J Physiol Endocrinol Metab 286:E201-207. 10.1152/ajpendo.00205.2003
2. Out C et al (2015) Gut microbiota inhibit Asbt-dependent intestinal bile acid reabsorption via Gata4. J Hepatol 63(3):697-704. doi: 10.1016/j.jhep.2015.04.030
3. Ferreira M et al (2005) Bile acids are toxic for isolated cardiac mitochondria: a possible cause for hepatic-derived cardiomyopathies? Cardiovasc Toxicol 5:63-73
4. * Praslickova D et al (2012) The ileal lipid binding protein is required for efficient absorption and transport of bile acids in the distal portion of the murine small intestine. PLoS One 7(12):e50810. doi: 10.1371/journal.pone.0050810
5. Recknagel P et al (2012) Liver dysfunction and phosphatidylinositol-3-kinase signalling in early sepsis: experimental studies in rodent models of peritonitis. PLOS Med 9:1001338. doi: 10.1371/journal.pmed.1001338
6. * Stelzner M et al (2001) Systemic effects of acute terminal ileitis on uninflamed gut aggravate bile acid malabsorption. J Surg Res 99(2):359-64. doi: 10.1006/jsre.2001.6137
7. * Xiao YT et al (2016) Altered systemic bile acid homeostasis contributes to liver disease in pediatric patients with intestinal failure. Sci Rep 6:39264. doi: 10.1038/srep39264

**Topic 17 Other pathophysiological mechanisms**

RCT = 1

Observational = 1

Review = 2

Case series = 0

Other = 4

Papers identified from electronic databases (1):

1. Reddy S et al (2016) Effect of saline 0.9% or Plasma-Lyte 148 therapy on feeding intolerance in patients receiving nasogastric enteral nutrition. Crit Care Resusc 18(3): 198-204

Additional papers identified (7):

Observational studies

1. Zhao C et al (2017) Acute Colonic Pseudo-Obstruction with Feeding Intolerance in Critically Ill Patients: A Study according to Gut Wall Analysis. Gastroenterol Res Pract 2017:9574592. doi: 10.1155/2017/9574592

Reviews

1. Reintam Blaser A et al (2018) Perioperative gastrointestinal problems in the ICU. Anaesthesiol Intensive Ther 50(1):59-71. doi: 10.5603/AIT.a2017.0064
2. * Verbrugge FH et al (2013) Abdominal contributions to cardiorenal dysfunction in congestive heart failure. J Am Coll Cardiol 62(6):485-95. doi: 10.1016/j.jacc.2013.04.070

Other

1. * Gorrasi J et al (2017) Perioperative Fluid Accumulation Impairs Intestinal Contractility to a Similar Extent as Peritonitis and Endotoxemia. Shock 50(6):735-740. doi: 10.1097/SHK.0000000000001088
2. * Lee TC, Huang YC, Lu YZ, Yeh YC, Yu LC (2018) Hypoxia-induced intestinal barrier changes in balloon-assisted enteroscopy. J Physiol 596:3411-3424. doi: 10.1113/JP275277
3. * Turnage RH, Guice KS, Oldham KT (1994) Endotoxemia and remote organ injury following intestinal reperfusion. J Surg Res 56(6):571-578. doi: 10.1006/jsre.1994.1091
4. Unthank JL, Bohlen HG (1988) Lymphatic pathways and role of valves in lymph propulsion from small intestine. Am J Physiol 254(3 Pt 1):G389-398. doi: 10.1152/ajpgi.1988.254.3.G389
