# Supplementary material for: Gastrointestinal dysfunction in the critically ill: a systematic scoping review and research agenda proposed by the Section of Metabolism, Endocrinology and Nutrition of the European Society of Intensive Care Medicine
Source: Crit Care. 2020 May 15;24:224. doi: 10.1186/s13054-020-02889-4 (PMC7226709; doi:10.1186/s13054-020-02889-4)
Supplement: Supplementary file 3 — Additional file 3: All study proposals. This file includes Table S4. presenting all study proposals. [file 13054_2020_2889_MOESM3_ESM.docx]

**Additional file 3. All study proposals.**

**Table S4. Study proposals.**

| Rank Vote1 | Rank Vote2 | Study acronym | Research questions/aims | Study design | Subjects | | Details/  Intervention | Outcome variables | Comments /  open questions |
| --- | --- | --- | --- | --- | --- | --- | --- | --- | --- |
| DIAGNOSIS OF GI DYSFUNCTION | | | | | | | | | |
| A. Epidemiology, classification and prognosis | | | | | | | | | |
| 24-28 | NA | GI - doc | How are GI symptoms monitored in the ICU | International survey | Nurses, dietitians and physicians working in ICUs | | Questionnaire on practices worldwide | List of GI symptoms with operational definitions. Monitoring methods.  Differences between continents/regions and nurses, dietitians vs physicians. | Using ESICM mail based survey platform (an anonymous survey but still send reminders to non-responders). Could be the first step in consensus process. |
| 9-11 | 9 | AGI prospective | Does subjectively given AGI score (AGI I-IV) predict the outcome? | Prospective observational | Consecutive ICU patients being mechanically ventilated for non-elective reason (planned MV after elective surgery excluded) | | AGI score documented daily. Decisions for diagnostics or treatment taken based on daily assessed GI symptoms * documented daily. | GI symptoms *, pneumonia, COS^#^,  ICU outcome, 90-day outcome, long-term patient-centered outcome, NOBN, GI anastomosis leakage (if relevant) | No standard definition of gastric FI (a part of AGI score).  Preferably adoption of a similar feeding protocol (& suggestions for prokinetics) in all centers. |
| 21-23 | NA | FI prediction | Which clinical symptoms are associated with FI? | Prospective observational | ICU patients with EN. Unified feeding protocol | | Daily (or 8-12-hrly) documentation of GI symptoms and EN | Success of EN (% of target), GI symptoms *. Symptoms leading to reduction of EN. Association between GI symptoms (before EN) and subsequent FI | Definition of FI needs to be established a priori. FI to be assesssed by assessors blinded to previous clinical observations, ideally not a team member |
| 1. Assessment of motility | | | | | | | | | |
| 12-17 | 15 | GI diagnosis | 1. Is motility, bowel diameter and wall thickness as assessed by US associated with GI symptoms * and outcome?  2. Can clinical assess-ment and abdominal US predict adverse outcomes? | Prospective observational | 1. Adult ICU patients  2. Undergoing planned abdominal CT or laparotomy/ scopy | | 1. Clinical examination with vs. without ultrasound.  GRV and IAP measured  2. computed tomography and/or surgical findings. US performed before CT/surgery | 1. GI symptoms *, feeding intolerance, success of EN, ICU and hospital outcome  2. Verified clinical outcome:  1) Gut distension (stomach/ small/large bowel)  2) Perforation  3) Peritonitis  4) Gut ischemia | Clinical and radiological assessors need to be blinded for radiological and clinical results respectively. Ideally they are not part of the treating team. Potentially, this study may help to define and grade FI. |
| 7-8 | 11 | US-Gastric 1 | Is US a reliable technique for routine bedside assessment of gastric emptying? | Multicenter observational | Ventilated adult ICU patients with tube feeding | | Repeated US assess-ments against labeled carbon absorption and exhalation (and GRV) | Correlation of US-assessed changes in gastric volume and labeled carbon exhalation and GRV |  |
| 24-28 | NA | US-Gastric 2 | Should US replace GRV measurements in diagnosing of feeding intolerance? | Randomized controlled trial | Ventilated adult ICU patients in need of EN expected to stay for >= 3 days. | | GRV measurements vs. US assessment vs. standard care (in centres without GRV measurement). | Success of EN; amount of calories delivered  GI symptoms *  Pneumonia  Mortality | Operator dependency. Requires US-training.  After US-Gastric 1. Mea-surement of gastric empty-ing (e.g. scintigraphy)? |
| 12-17 | 17 | US-Bowel 1 | Can US be used for assessment of  a) bowel peristalsis (and EN tolerance)?  b) bowel distension and bowel wall thickness? | Proof-of- concept  Observational study | a) Adult ICU patients in need of tube feeding expected to stay for at least 3 days  b) with indication for abdominal CT | | US daily  Abdominal ultrasound. Observer blinded to CT-scan results  Measurement of bowel motility (e.g. gastro-graphin X-ray and /or high-resolution mano-metry large bowel) | Success of EN, GI symptoms *  Correlation of US and clinical symptoms with CT scan results (Comparison of small and large bowel diameter and wall thickness US vs. CT and X-ray) | Potential difficulties to correlate US location with CT-scan.  Radiologist blinded for the other investigation. |
| 31 | NA | Balloon 1  (Gastric motility measurement as readout of gastric emptying) | Is measurement of gastric motility mea-sured by intra-gastric balloon safe and does it correlate with gastric emptying? | Prospective interventional | Ventilated adult ICU patients with tube feeding | | Establish the relationship between gastric motility and gastric emptying (measured by 13C-octanoate breath test) | Correlation between gastric motility and emptying  Safety: bleeding/ulcers/ long-term stenosis potentially explained by balloon inflation | Reference for intragastric balloon measurement: Janssen et al. Neurogastro-enterology & Motility 2018 |
| 32 | NA | Balloon 2  (Gastric motility measurement versus GRV) | Is measurement of gastric motility with intragastric balloon superior to GRV for monitoring FI? | Multicenter RCT | Ventilated adult ICU patients in need of EN and expected to stay >= 3d in ICU | | Standard feeding protocol (incl. GRV) vs. EN guided by motility measurement (no GRV) | Time to reaching feeding target. Use of PN  Safety: aspiration, pneumonia, non-occlusive bowel ischemia | Only after the above-mentioned pilot study. |
| 1. Biomarkers | | | | | | | | | |
| 1 | 14 | Bio-  markers for GI dysfunction | Validate biomarkers for absorption of nutrients and GI dysfunction | Prospective observational multicenter | Ventilated adult ICU patients with tube feeding expected to stay for at least 3 days | | BOMB calorimetry of feces (24-h fecal coll.), Increase in plasma AA, D lactate, Fecal fat/fatty acids, stool details (Bristol scale, volume, pH). Absorp-tion (3-OMG). I-FABP. Citrulline. Lactulose/ rhamnose or cellobiose /rhamnose test | COS ^#^  Associations between biomarkers and clinically relevant outcomes, or scores (e.g. SOFA) | Gold standards for absorption of specific nutrients? Stool specific analyses for malabsorption of different macronutrients to be considered/specified.  Potentially additional biomarkers based on iSOFA results |
| 24-28 | NA | US-Bowel 2 (+ Bowel-inflame) | Define jejunal feeding intolerance. Are clinical symptoms and biomarkers associated with US findings? Are bowel distension and wall thickness related to markers of gut barrier loss & systemic inflammation? | Observational | Adult ICU patients in need of EN with expected ICU stay of at least 3 days | | Progressive EN, stan-dard protocol.  Repetitive US to identify small bowel diameter and wall thickness. Gut permeability biomar-kers & tests, and markers of systemic inflammation (CRP, IL-6, TNF, IL-1β …) | Association between small and large bowel diameter/wall thickness (US/CT) with symptoms of FI, compliations of EN (incl. NOMI) and biomarkers of gut barrier loss, permeability & markers of systemic inflammation | Time points of US measurements to be defined |
| 2-3 | 12 | Gut barrier and EN | To study whether early EN is able to preserve intestinal wall integrity | RCT  Early EN vs early PN (isocaloric and in both groups slow progression to target) | Ventilated ICU patients on vasopressor support, after stabilisation of shock | | Early EN vs early PN  Measurement of I-FABP; I-BABP; zonulin-1, citrullin, lactulose/ rhamnose or cello-biose/ rhamnose), duodenal biopsies if feasible. Indirect calorimetry. | Inflammatory and gut permeability markers, Endotoxin, bacterial DNA, peptidoglycans in blood. Monocyte activation. CD-4 T cell reactivity. Infections. NOMI. 90d outcome.  Hypermetabolic response. | Timing of measurements to be defined |
| 2-3 | 20 | Biomarkers of intestinal ischemia and barrier function in ICU | Validate biomarkers in predicting mesenteric ischemia in ICU patients.  Barrier function: characterize epithelial first and second line defense in critically ill patients with intestinal ischemia | Observational large scale cohort study.  Subgroups: 1) abdominal surgery  2) medical ICU pt (with shock) | Consecutive adult MV ICU pt with increased risk of intestinal ischemia | | I-FABP, α-GST, SMA, citrulline, D-lactate, sCD14, Claudin 3, RT PCR Bacteroides and Entero-coccus sp. Stool analysis (mucins, AMPs, IgA, eosinophil cationic protein, calprotectin). Analysis of intest. fluid (endosc). | Predictive value of biomarkers to diagnose mesenteric ischemia – confirmed by endoscopy or surgery. Link with short and long term intestinal, vital and functional outcome (including Sepsis, LOS ICU and hospital, 28 & 90 days mortality). Association between clinical symptoms and biomarkers. | Systematic review needed to define study group (risk pt).  All patients with suspected ischemia (e.g. GI bleeding) need endoscopy if no surgery.  Endoscopy or surgery needed for final diagnosis of mesenteric ischemia. |
| 9-11 | 13 | Pancreatic Insufficiency in ICU | What is the real incidence and prevalence of exocrine pancreatic enzyme deficiency in ICU patients? | Prospective observational | Adult ICU patients receiving EN | | None | GI symptoms *  Success of EN  Fecal fat  Elastase-1 stool  Serum trypsinogen  BOMB calorimetry | Additional option to study the correlation with pancreatic endocrine function (C-peptide). Consider assessment of small bowel fluid in patients undergoing endocsopy |
| PATHOPHYSIOLOGICAL MECHANISMS OF GI DYSFUNCTION | | | | | | | | | |
| Rank Vote1 | Rank Vote2 | Study acronym | **Research questions/aims** | **Study design** | **Subjects** | | **Details/**  **Intervention** | **Outcome variables** | **Comments /**  **open questions** |
| 29-30 | NA | Monitoring of splanchnic perfusion | To validate US as non-invasive tool for monitoring of splanchnic perfusion | 1. Experimental study. Occlusion of sup. mesent. artery in large animal  2. Clinical observational | 1. Large animal  2. Pt with endoscopy or surgery planned because of suspected ischemia | | Doppler flow of intestinal wall | Predictive value of US findings vs. findings at endoscopy and/or surgery | Consider validation in patients with an existing ileostoma or colostoma allowing direct monitoring (visual, micro-circulation Doppler, biopsies). |
| 18-20 | 19 | BA-MA | To validate bile acid signaling molecules as biomarkers for malabsorption | Prospective, multicenter observational study | MV ICU pt expected stay for >=3 days | | Measurement of biomarkers (serum BA, FXR, FGF-19). 3-OMG. Cholestasis-parameters,  BOMB-calorimetry | Association between BAs (and regulators/ligands) and malabsorption. Associations between clinical symptoms and bile acid metabolites | Timing of measurements to be defined.  Definition/reference standard for malabsorbtion of different macronutrients needed. |
| 21-23 | NA | Fluids 1  (GI function in liberal vs rest-rictive fluids) | Does restrictive fluid therapy reduce GI dysmotility (result in less GI symptoms)? | Systematic review and meta-analysis | All studies restrictive vs liberal. Subgroup of adult ICU pt | | NA | - GI symptoms *  - Success of EN  - MV duration  - mortality, infections | Data on GI symptoms and EN possibly not always available. Clear-cut definition between restric-tive and liberal needed. |
| 12-17 | 16 | Fluids 2 | Are bowel distension and wall thickness and dysmotility related to vasopressor dose and fluid balance? | Observational study | ICU patients scheduled for abdominal CT scan | | Vasopressor dose and  (cumulative) fluid balance | Association between small and large bowel diameter/wall thickness (US/CT) and vasopressor dose/cumulative fluid balance | Vasopressor dose difficult to quantify in patients receiving different vaso-pressors. A priori agree-ment on the defini-tion of vasopressor dose and (cumulative) fluid balance |
| 21-23 | NA | Opioids and GI  (Liberal vs restrictive opioids) | Does restrictive opioid regimens improve GI dysmotility (symptoms)? | RCT | Adult ICU patients (MV, no GI surgery, predicted LOS above 48 hrs) | | Different opioid regimens | - GI symptoms *. Pain  - Success of EN  - LOS  - Duration of MV  (- Bowel diameter x ray/US) | Patient group & intervention to be defined. Pain scale target – identical, non-opioid strategy standardized |
| 18-20 | 8 | GI and IAP | Which GI symptoms * should trigger IAP measurements?  Which IAP values should trigger specific monitoring of GI? | Post-hoc analysis of combined databases (prospective observational) | Patients in performed studies IROI, iSOFA)? | | identification and merging of existing databases (iSOFA, IROI, others?) | Association of GI symptoms with IAH, mesenteric ischemia and mortality |  |
| 12-17 | 6 | IAH-GI + NOMI-AGI | 1) Does protocolised monitoring of IAP and management of IAH improve outcome?  2) Is increased IAP associated with GI dysmotility? | 1) RCT  2) observational substudy | MV patients at risk of IAH. | | Intervention: Monitoring and management of IAP based on the protocol (bundle of preventive measures). Control: standard care.  US in intervention group | Mesenteric ischemia  Incidence of infections / sepsis  Mortality 90d, LOS, GI symptoms*  Obervational substudy:  Correlation between IAP and GI motility as assessed by US |  |
| 9-11 | 18 | AGIbiome (+Abxbiome) | Identification of intestinal microbiome signatures and correlation with AGI and SOFA score | Prospective, observational | >200 ICU patients with EN and/or PN | | Microbiome (multiple body site) sampling  Effects of different antibiotics / feeding routes | Variability and specificity of dysbiosis patterns according to different AGI grades  Intra- and interindividual microbial changes | Large sample size required  Adjustment for different (antimicrobial) treatment  Standardized sampling and analysis to be established |
| 12-17 | 10 | PPI and dysbiosis | Does usage of PPI versus no PPI alters the intestinal microbiome? | RCT | Mechanically ventilated ICU patients in need of EN and without an absolute indi-cation for PPI | | Faecal microbiome  Clostridium difficile infection | Faecal microbiome pattern  Incidence of Clostridium difficile colitis | Large sample size required if also powered for clinical endpoints |
| PREVENTION AND MANAGEMENT | | | | | | | | | |
| Rank Vote1 | Rank Vote2 | Study acronym | **Research questions/aims** | **Study design** | **Subjects** | | **Details/**  **Intervention** | **Outcome variables** | **Comments /**  **open questions** |
| 6 | 4 | Prophylaxis vs Treatment of upper GI intolerance | Is the prophylactic use of prokinetics superior to therapeutic use? | Multicenter RCT | Adult ICU pt. at high risk of gastroparesis | | Two study arms, same drugs (erythromycin, metoclopramide, alizapride, ..) and dosa-ges, different timing (routine vs confirmed gastroparesis) | COS ^#^  Pneumonia incidence  Long-term outcomes  Adverse effects (prolonged QT, extrapyramidal side effects, colonisation with multi-resistant microbes) | Patients at risk may include patients receiving high doses of opioids, patients having undergone GI surgery, and patients with peritonitis, sepsis, diabetes or recovering from shock |
| 12-17 | 5 | Prophylaxis vs Treatment of lower GI intolerance | Does the prophylactic use of motility agents (prokinetics and laxative drugs) reduce time to defecation, improve feeding tolerance and GI dysfunction based on AGI grading? | Multicenter RCT | Adult consecutive ICU patients with with expected stay of >3d? | | Two study arms, same drugs (e.g. Macrogol, Laxatives) and dosages, different timing (routine administration or only in confirmed constipation/ bowel paralysis) | AGI dynamics  COS (clinical outcomes)  Infections  Diarrhoea  Mesenteric ischemia | Study duration to be defined |
| 7-8 | 7 | Indication of postpyloric feeding | Is postpyloric feeding superior to PN in case of gastroparesis? | Multicenter RCT | Adult ICU patients with gastroparesis (e.g. GRV>500ml with prokinetics) | | Postpyloric feeding versus PN | Infections  Gastrointestinal complications (including non-occlusive bowel ischemia)  Mortality  Meeting nutritional target |  |
| 24-28 | NA | Novel pro-motility drugs | Are there novel pro-motility drugs with comparable effect and more appealing adverse effect profile? | Proof of principle con-firming stu-dies through to multicenter CTs | Depends on phase of trial | Study drug vs placebo or other drug? | | Primary outcomes: safety profile, nutritional delivery, feed-tolerance and gastric emptying. Secondary outcome: patient-centred outcomes | Addresses Inadequate secretion or effect of a GI hormone that may respond to interventions that augment secretion to ‘normal’ levels |
| 4-5 | 1 | Diarrhea prevention | Does routine use of fiber feeds reduce diarrhea? | RCT | Critically ill patients in need of EN | Fiber feeds versus non-fiber feeds | | Bristol stool chart  GI symptoms * | Standard protocol for diarrhea management is advisable.  Study EN solution to be discussed (mixed fibers vs. only soluble fibers) |
| 4-5 | 3 | Diarrhea management | Does reduction or discontinuation of EN reduce diarrhea? | RCT (3-armed study) | Patients with severe diarrhea during EN | 1. Continuation of EN  2. Reduction of EN by 50%  3. Trophic EN + supplemental PN (after 3-7 days) | | Bristol stool scale  GI symptoms *  LOS  infections | Severe diarrhoea = requiring interventions (fluid, electrolyte replacement).  Use of laxatives needs to be standardized. |
| 29-30 | NA | Colitis management | Should patients with colitis receive enteral fluids or EN? Timing of EN? | RCT (3-armed study) | Adult ICU patients with colitis | 1. clear fluids (PN after 7d)  2. trophic EN for 7 days  3. full EN  4. PN | | GI complications (bleed, perforation, interventions)  Systemic infections  Bacterial translocation  LOS  Mortality | Criteria for diagnosis of colitis to be established |
| 18-20 | NA | Opioid-antagonists for bowel paralysis | Do opioid antagonists reduce time to defecation and GI symptoms?  (Validate opioid antagonists for constipation in ICU)  Potential substudy: study the impact on intestinal absorption | Multicenter RCT | Adult ICU patients with opioid requirement above a minimal dosage | Methylnaltrexone vs Placebo.  Other opioid antagonists. | | Time to first defecation  COS #  Diarrhoea  GI symptoms *  (Absorption)  Infections |  |
| 24-28 | NA | Splanchnic vasodilators | To identify effect of splanchnic vasodilators on splanchnic perfusion | Observational cohort study | MV ICU patients with risk or evidence of borderline intestinal flow. | Potential study drugs: dopaminergic agonists, iloprost, NO donors, PDE inhibitors, ACE inhibitors, glucagon analogs,  Endothelin receptor inhibitor, C5a inhibitors,..  Splanchnic perfusion assessed by  US/ Doppler bedside | | Change in splanchnic perfusion after initiation of study drug  Clinical outcome measures  Signs of non-splanchnic hypoperfusion due to „steal-effect“ (cardiac ischemia, peripheral poor circulation,..)  Potential biomarkers of intestinal damage | Can only be performed after having validated bedside US Doppler or any other bedside method as measure of splanchnic perfusion. |

All study proposals were included in Voting 1, and 20 studies receiving highset ranking in Votibg 1 were included in Voting 2. Voting procedure is described in Supplement 1.

α-GST – α-glutathione S-tranferase; AA – amino acids; AGI – acute gastrointestinal injury; BA – bile acids; CH - carbohydrate; COS – core outcome set for studies on GI (dys)function; CRP – C-reactive protein; CT – computed tomography; EN – enteral nutrition; FGF-19 - Fibroblast Growth Factor 19; FI – feeding intolerance; FXR – Farnesoid X receptor; GI – gastrointestinal; GRV – gastric residual volume; IAH – intra-abdominal hypertension; IAP – intra-abdominal pressure; IC – indirect calorimetry; ICU – intensive care unit; I-FABP – intestinal fatty acid binding protein; IL - interleukin; LOS – length of stay; MV – mechanical ventilation; NOMI – non-occlusive mesenteric ischemia; PN – parenteral nutrition; PPI – proton pump inhibitors; RCT – randomized controlled trial; RT PCR – real time polymerase chain reaction; SMA – smooth muscle actin; SOFA – sequential organ failure assessment; TNF – tumor necrosis factor; US – ultrasound; 3-OMG - 3-O-methylglucose

* GI symptoms include vomiting/regurgitation, abdominal distension, GI bleeding, diarrhoea, lower GI paralysis ^1^. Expanded (if performed/possible to assess): nausea, abdominal pain, absence of bowel sounds, large GRV (>500 ml/6h), bowel dilatation (radiological), bowel wall thickening/bowel edema (radiological) ^1,2^

# Core outcome set (COS) to be identified in consensus process

1. Reintam Blaser A, Malbrain ML, Starkopf J, Fruhwald S, Jakob SM, De Waele J, Braun JP, Poeze M, Spies C. Gastrointestinal function in intensive care patients: terminology, definitions and management. Recommendations of the ESICM Working Group on Abdominal Problems. Intensive Care Med. 2012 Mar;38(3):384-94. doi:10.1007/s00134-011-2459-y
2. Reintam Blaser A, Starkopf J, Moonen PJ, Malbrain MLNG, Oudemans-van Straaten HM. Perioperative gastrointestinal problems in the ICU. Anaesthesiol Intensive Ther. 2018;50(1):59-71. doi: 10.5603/AIT.a2017.0064
