# Supplementary material for: Gastrointestinal dysfunction in the critically ill: a systematic scoping review and research agenda proposed by the Section of Metabolism, Endocrinology and Nutrition of the European Society of Intensive Care Medicine
Source: Crit Care. 2020 May 15;24:224. doi: 10.1186/s13054-020-02889-4 (PMC7226709; doi:10.1186/s13054-020-02889-4)
Supplement: Supplementary file 4 — Additional file 4: Monitoring and motility. This file includes summary on monitoring of GI function, biomarkers of GI dysfunction with description of specific aspects and pifalls in laboratory measurements, and summary of drugs influencing GI motility. Table S5. presents clinical assessment, imaging and specific tools used to assess motility and perfusion. Table S6. presents possible laboratory biomarkers of GI dysfunction. Table S7. presents summary on GI motility drugs based on systematic review. [file 13054_2020_2889_MOESM4_ESM.docx]

**Additional file 4. Monitoring and motility.**

Contents:

1. Monitoring of GI function (Table S5) with references………………….page 1-4
2. Biomarkers of GI dysfunction (Table S6) with references……………...page 5-6
3. Specific aspects of laboratory measurements with references…………..page 7-10
4. GI motility drugs (Table S7)……………………………………….........page 11-12

**Table S5. Clinical assessment, motility, imaging and perfusion in monitoring of GI function**

| **Modality** | **Sign/**  **Technique** | **Description** | **Pro** | **Con** |
| --- | --- | --- | --- | --- |
| General abdominal signs and symptoms | Abdominal pain | The type, location, chronology and duration can help in diagnosis | - Key symptom in acute events - Can be scored - Can be located | - Impossible in the unconscious patient [1, 2] - Non-specific and subjective - Incidence and association with outcome are unknown [2] |
|  | Abdominal distention | A non-specific sign | - Can be a sign of increased abdominal volume | - Impossible to measure/see in the (morbidly) obese - Clinical relevance depends on other clinical findings [3] |
|  | Bowel sounds | The presence/absence may indicate presence or absence of peristalsis | Presence and type of bowel sounds can be indicative for the presence of pathology (e.g. ileus, obstruction) [4] and is possibly associated with disease severity and related outcome [3,5] | - No correlation between return of bowel sound and restored motility. - Importance and reliability are subject for debate [4] - No uniform definitions on bowel sounds, subjective [6] |
|  | Abdominal palpation | Systematic palpation, after auscultation | - Locating pain, masses - Guarding or rebound can indicate peritonitis [7] | - Post-operative abdomen more sensible to touch - Subjective interpretation, unreliable in sedation |
|  | Abdominal percussion | Presence and location of tympanism and pain | - Allocating peritoneal irritation - Hypertympanism may indicate bowel dilatation/distension or free air (perforation) | - Subjective - Reliability postoperative and in sedated patients is unclear |
|  | Vomiting | Visible regurgitation of gastric content | - Can be a sign of disease, medication (ab)use, ileus, gastroparesis - May cause pneumonia [8] | - Non-specific sign - Can be missed in ventilated patients [6] |
|  | Diarrhoea | Three or more loose/ liquid stools daily with a stool weight >250 g/day. | Can be a sign of   - - intestinal infection/ischemia [9]   - malabsorption [9] | - High prevalence in ICU, relevance highly depended on a case by case evaluation [6,9] - Can be caused by laxatives [2,6,9] |
|  | Lower GI paralysis | The absence of bowel movements for ≥3 days | - Can be a sign of obstruction, peritonitis [10] - Can be drug-induced [10] | - Unreliable in postoperative phase (inevitable in abdominal surgery) [10] |
|  | Bowel dilatation | Colonic diameter >6 cm (>9 cm for caecum) or small bowel >3 cm (2,6) | - Common sign of obstruction and Ogilvie’s syndrome [2,6] | - Can be non-specific in nature and its relevance unsure - Detection requires imaging [2,6] |
|  | Abdominal dis-coloration | Abdominal wall discoloration associated with local bleeding, infection or pancreatitis | - Specific locoregional sign - Cullen sign around the umbilicus - Grey Turner sign on the flank [2] | - Seldomly seen - Impact or relevance on course of the disease is unknown |
|  | GI bleeding | Any macroscopically evident bleeding into the GI tract lumen | - can be a clinically relevant symptom influencing outcome [11] | - may not be related to GI dyfunction as a part of multiple organ dysfunction in critical illness (e.g. oesophageal variceal bleeding, gastric / duodenal ulcus) [12,13] |
| Gastric emptying | Gastric residual volume | Estimate of gastric emptying | - Easily performed by nurses [14] - Low cost [14] | - Usefulness and accuracy are unclear [15, 16] - Neither validated nor standardized measurement [15,16] |
|  | Tests based on detection of tracers | Tracers or markers infu-sed via NG-tube, move-ment or absorption de-tected by scintigraphy, in blood or exhaled air | - Accurate [17, 18, 19] | - Special equipment needed [18, 19] - Time consuming and expensive [18,19] - Radioactivity [19] - Results depend on both gastric emptying and absorption [20] |
|  | Refracto-metry | Measurement of gastric fluid refractive index to assess its composition | - Can discriminate between gastric fluid and (liquid) meal [21] - Easy and cheap [21] | - Validation in broader clinical setting is missing - Gastric emptying is not directly measured |
|  | Scinti-graphy |  | - Gold standard [17, 19] | - Time- and work-expensive [19] - Not applicable for everyday use [17,19] |
|  | Ultrasound | Measurement of gastric diameter and estimation of volume | - Relative cheap, accurate, non-invasive and accessible [22, 23] | - Highly investigator dependent and can be influenced by patient and liquid nutrient factors [22, 24] |
| Intestinal motility | Acoustic GI surveillance biosensor | Detection of post-operative ileus (POI), using a microphone to detect bowel sounds | - Non invasive [25, 26] - Precise detection of bowel sounds - Easy in use [25, 26] | - Availability - No or barely validation for clinical use and in ICU setting [25, 26] |
|  | High-resolution fiberoptic manometry | Visualization of colonic motility pattern | - New method - Tested in healthy subjects and in patients after hemicolectomy [27, 28] | - Not used in ICU setting [27, 28] |
| Imaging techniques | Ultrasound | Capturing reflection of ultrasound waves to create moving images | - No x-rays, relative cheap equipment - Direct dynamic bedside visualization - Can evaluate all aspects of intra-abdominal wall and organs [29, 30] | - Investigator dependent - Influenced by gastrointestinal contents and patient morphology [29, 30] |
|  | X-ray | Plain X-ray | - Can detect obstruction, free air, obstipation, bowel diameter, fluid levels (as marker of stasis) - Cheap, easily accessible | - Exposure to radiation - Low sensibility and sensitivity in comparison to CT, ultrasound and MRI in evaluation of free fluid / solid and hollow organ |
|  | CT-scan | With or without iv or intraluminal contrast | - Imaging of the entire abdomen - Can evaluate intra-abdominal free fluid, perfusion, organ status, inflammation? [29] - Good image quality [29] | - More expensive - Patient transport to the radiology department - Higher exposure to X-rays - Use of IV/PO contrast + risk of allergic reactions [30] |
|  | MRI | Magnetic resonance imaging of specific organs (e.g. MRCP) or the entire abdomen | - High image quality [31] - Advanced diagnostic imaging | - Expensive - Not readily accessible in every hospital - Long duration of investigation - Patient transport to the radiology department - Use of IV/PO contrast [30] |
| Perfusion | Refractance spectro-photometry | Refraction of light to measure the average Hb oxygen saturation of blood in the GI tract | - Real-time monitoring [32] - Gives a direct estimate of oxygen delivery [32] | - Does not measure bloodflow - Close range detection in 90° angle - Influenced by several factors [32] |
|  | Infra-red spectro-scopy | Near infrared (NI) light used to measure Hb oxygenation. | - Less invasive [33] - Transcutaneous | - Accessibility of the liver, subcutaneous fat and edema [33] - Inter-individual variation in single-point tissue oxygenation |
|  | Gastro-intestinal tonometry | Using the diffusion of CO_2_ from the surrounding tissue into the gastric lumen to estimate gut perfusion | - Correct measurement [34] - CO_2_ gap accurate measure for hypoperfusion [35] | - Technical and measurement errors (either continuous or in blood gas analyzer) [36] - Prone to procedural errors, incl. catheter position - Influenced by enteral feeding and medication [33] |
|  | Laser Doppler flowmetry | Measurement of microcirculatory blood flow using Doppler shift | - Can be used intra-operative - Accurate measurement [33] | - Unable to measure absolute blood flow [33] - Unable to detect flow in individual vessels [36] |
|  | Videomic-roscopic imaging | Measures vascular density, heterogeneity of perfusion and micro-vascular blood flow | - Direct access - Easily performed - Can evaluate microcirculation [36] | - Secretions and movement artifacts influence imaging of the microvessels - Investigation is limited to the sublingual area or stoma [36] |

Cm – centimetre; CO_2_ – Carbon Dioxide; CT – Computed Tomography; g- Gram; GI – Gastrointestinal; Hb – Haemoglobin; ICU – Intensive Care Unit; IV – Intravenous; MRCP – Magnetic Resonance Cholangiopancreatography; MRI – Magnetic Resonance Imaging; NG – Nasogastric; PO – per oral

Definitions of GI symptoms in this Table are based on reference 6.

**References for Table S5**

1. Moonen PJ, Reintam Blaser A, Starkopf J et al (2018) The black box revelation: monitoring gastrointestinal function. Anaesthesiol Intensive Ther 50(1):72-81. doi: 10.5603/AIT.a2017.0065
2. Reintam Blaser A, Starkopf J, Malbrain ML (2015) Abdominal signs and symptoms in intensive care patients. Anaesthesiol Intensive Ther 47(4):379–387. doi: 10.5603/AIT.a2015.0022
3. Reintam Blaser A, Poeze M, Malbrain ML et al (2013) Gastrointestinal symptoms during the first week of intensive care are associated with poor outcome: a prospective multicentre study. Intensive Care Med 39(5): 899–909. doi: 10.1007/s00134- 013-2831-1
4. Li B, Tang S, Ma YL, Tang J, Wang B, Wang JR (2014) Analysis of bowel sounds application status for gastrointestinal function monitoring in the intensive care unit. Crit Care Nurs Q 37(2):199-206. doi: 10.1097/CNQ.0000000000000019
5. Reintam A, Parm P, Kitus R et al (2009) Gastrointestinal symptoms in intensive care patients. Acta Anaesthesiol Scand 53(3): 318–324. doi: 10.1111/j.1399-6576.2008.01860.x
6. Reintam Blaser A, Malbrain ML, Starkopf J et al (2012) Gastrointestinal function in intensive care patients: terminology, definitions and management. Recommendations of the ESICM Working Group on Abdominal Problems. Intensive Care Med 38(3): 384–394. doi: 10.1007/ s00134-011-2459-y
7. Staniland JR, Ditchburn J, De Dombal FT (1972) Clinical presentation of acute abdomen: study of 600 patients. Br Med J 12;3(5823):393-398. doi: 10.1136/bmj.3.5823.393
8. Metheny NA, Clouse RE, Chang YH, Stewart BJ, Oliver DA, Kollef MH (2006) Tracheobronchial aspiration of gastric contents in critically ill tube-fed patients: frequency, outcomes, and risk factors. Crit Care Med 34(4):1007-15. doi: 10.1097/01.CCM.0000206106.65220.59
9. Reintam Blaser A, Deane AM, Fruhwald S (2015) Diarrhoea in the critically ill. Curr Opin Crit Care 21(2): 142–153. doi: 10.1097/ MCC.0000000000000188
10. Fruhwald S, Holzer P, Metzler H (2008) Gastrointestinal motility in acute illness. Wien Klin Wochenschr 120: 6−17. doi: 10.1007/s00508007-0920-2
11. Cook DJ, Griffith LE, Walter SD, et al. (2001) The attributable mortality and length of intensive care unit stay of clinically important gastrointestinal bleeding in critically ill patients. Crit Care 2001;5:368-375
12. D'Hondt A, Haentjens L, Brassart N, Flamme F, Preiser JC (2017) Uncontrolled bleeding of the gastrointestinal tract. Curr Opin Crit Care 23(6):549-555. doi: 10.1097/MCC.000000000000045
13. Ovenden C, Plummer MP, Selvanderan S, et al. (2017) Occult upper gastrointestinal mucosal abnormalities in critically ill patients. Acta Anaesthesiol Scand 61(2):216-223. doi: 10.1111/aas.12844
14. Van Stappen J, Pigozzi C, Tepaske R et al (2014) Validation of a novel method for measuring intra-abdominal pressure and gastric residual volume in critically ill patients. Anaesthesiol Intensive Ther 46(4): 245–254. doi: 10.5603/AIT.2014.0042
15. Elke G, Felbinger TW, Heyland DK (2015) Gastric residual volume in critically ill patients: a dead marker or still alive? Nutr Clin Pract 30(1):59–71. doi: 10.1177/0884533614562841
16. Reignier J, Mercier E, Le Gouge A, et al (2013) Effect of not monitoring residual gastric volume on risk of ventilator-associated pneumonia in adults receiving mechanical ventilation and early enteral feeding: a randomized control- led trial. JAMA 309(3):249–256. doi: 10.1001/jama.2012.196377
17. Chapman MJ, Besanko LK, Burgstad CM et al (2011) Gastric emptying of a li- quid nutrient meal in the critically ill: relationship between scintigraphic and carbon breath test measurement. Gut 60(10):1336–1343. doi: 10.1136/gut.2010.227934
18. Cohen J, Aharon A, Singer P (2000) The paracetamol absorption test: a useful addition to the enteral nutrition algorithm? Clin Nutr 19(4):233–236. doi: 10.1054/clnu.2000.0097
19. Kar P, Jones KL, Horowitz M et al (2015) Measurement of gastric emptying in the critically ill. Clin Nutr 34(4):557–564. doi: 10.1016/j. clnu.2014.11.003
20. Berger MM, Werner D, Revelly JP et al (2003) Serum paracetamol concentration: an alternative to X-rays to determine feeding tube location in the critically ill. JPEN J Parenter Enteral Nutr 27(2):151-155. doi: 10.1177/0148607103027002151
21. Chang WK, McClave SA, Hsieh CB et al (2007) Gastric residual volume (GRV) and gastric contents measurement by refractometry. JPEN J Parenter Enteral Nutr 31(1):63–68. doi: 10.1177/014860710703100163
22. Van de Putte P, Perlas A (2014) Ultrasound assessment of gastric content and volume. Br J Anaesth 113(1):12–22. doi: 10.1093/bja/aeu151
23. Manini ML, Burton DD, Meixner DD et al (2009) Feasibility and application of 3-dimensional ultrasound for measurement of gastric volumes in healthy adults and adolescents. J Pediatr Gastroenterol Nutr 48(3):287-93.
24. Perlas A, Chan VW, Lupu CM, Mitsakakis N, Hanbidge A (2009) Ultrasound assessment of gastric content and volume. Anesthesiology 111(1):82-9. doi: 10.1097/ALN.0b013e3181a97250.
25. Spiegel BMR, Kaneshiro M, Russell MM et al (2014) Validation of an acoustic gastrointestinal surveillance biosensor for postoperative ileus. J Gastrointest Surg 18(10): 1795–1803. doi: 10.1007/s11605-014-2597-y
26. Kaneshiro M, Kaiser W, Pourmorady J et al (2016) Postoperative gastrointestinal telemetry with an acoustic biosensor predicts ileus vs. uneventful GI Recovery. J Gastrointest Surg 20(1):132–139. doi: 10.1007/ s11605-015-2956-3
27. Lin AY, Dinning PG, Milne T, Bissett IP, O'Grady G (2017) The "rectosigmoid brake": Review of an emerging neuromodulation target for colorectal functional disorders. Clin Exp Pharmacol Physiol 44(7):719-728. doi: 10.1111/1440-1681.12760.
28. Vather R, O'Grady G, Lin AY et al (2018) Hyperactive cyclic motor activity in the distal colon after colonic surgery as defined by high-resolution colonic manometry. Br J Surg 105(7):907-917. doi: 10.1002/bjs.10808
29. Cartwright SL, Knudson MP, Cartwright SL et al (2008) Evaluation of acute abdominal pain in adults. Am Fam Physician 77(7): 971–978
30. Semelka RC, Armao DM, Elias J Jr, Huda W (2007) Imaging strategies to reduce the risk of radiation in CT studies, including selective substitution with MRI. J Magn Reson Imaging. 2007 25(5):900-909. doi: 10.1002/jmri.20895
31. Gangadhar K, Kielar A, Dighe MK et al (2016) Multimodality approach for imaging of non-traumatic acute abdominal emergencies. Abdom Radiol (NY) 41(1): 136–148. doi: 10.1007/s00261-015-0586-6
32. Friedland S, Soetikno R, Benaron D (2004) Reflectance spectrophotometry for the assessment of mucosal perfusion in the gastrointestinal tract. Gastrointest Endosc Clin N Am 14(3): 539–553, doi: 10.1016/j. giec.2004.03.011
33. van Haren FMP, Sleigh JW, Pickkers P et al (2007) Gastrointestinal perfusion in septic shock. Anaesth Intensive Care 35(5):679–694. doi: 10.1177/0310057X0703500505
34. Ackland G, Grocott MP, Mythen MG (2000) Understanding gastrointestinal perfusion in critical care: so near, and yet so far. Crit Care 4(5): 269–281. doi: 10.1186/cc709
35. Marshall AP, West SH (2004) Gastric tonometry and monitoring gastrointestinal perfusion: using research to support nursing practice. Nurs Crit Care 9(3):123–133 (
36. De Backer D, Ospina-Tascon G, Salgado D et al (2010) Monitoring the microcirculation in the critically ill patient: current methods and future approaches. Intensive Care Med 36(11):1813–1825, doi: 10.1007/ s00134-010-2005-3

**Table S6. Possible biomarkers for GI functioning in the critically ill based on systematic review on this subtopic.**

| **Biomarker** | **Description** | **Available evidence** | **Future perspectives Problems*** |
| --- | --- | --- | --- |
| **Intestinal ischemia/barrier function** | | **Sensitivity and specificity presented as taken from ref [1]** |  |
| **I-FABP** | Small protein bound within the cytoplasm of mature enterocytes at the villus tip. Released into blood when enterocyte integrity is compromised [1] | Sensitivity: 79.0 (66.5–88.5)   - Specificity 91.3 (87.0–94.6) - Good performance in several clinical studies [2-4]   Not all studies are positive [5] | Potential marker of ischemia.  Uncertainty as to how it will inform clinical decision-making |
| **α-GST** | A nonspecific oxidative stress marker released from various tissues during ischemia [1] | Sensitivity: 67.8 (54.2–79.5)   - Specificity 84.2 (75.3–90.9) - Very few clinical studies [6] | - Uncertain clinical utility due to nonspecific origin |
| **IMA** | Ischemia modified albumin is human serum albumin that is less capable of binding cobalt due to ischemia [1] | Sensitivity: 94.7 (74.0–99.9)   - Specificity 86.4 (65.1–97.1) Few clinical studies. | Uncertain clinical utility due to nonspecific origin |
| **SMA** | Smooth muscle actin is released from the intestinal muscularis during ischemia | Reported in one study to be superior to the other biomarkers at identifying transmural necrosis requiring surgery in patients post cardiac surgery [7] | Potential marker of ischemia.  Not clear how to use in clinical decision-making |
| **D-lactate** | Produced by colonic bacteria such as *Escherichia coli*, released into blood if mucosal injury [6] | Sensitivity: 71.7 (58.6–82.5)   - Specificity 74.2 (69.0–79.0) - Few clinical studies [4,8,9]   Not all studies are positive [7]. | - Poor stability, demands specific handling of blood samples |
| - **Enterocyte function** | | | |
| **Citrulline** | Amino acid synthetized from glutamine in the mitochondria of mature enterocytes, converted into arginine in kidneys. | Sensitivity: 100%  Specificity 39%  Few clinical studies [10,11]. | - Interpretation complicated by increased concentrations in renal failure [12]. |
| - **Motility (Enterohormones)** | | | |
| **CCK** | Released from entero- endocrine cells predominantly in the proximal small intestine in response to nutrients (mainly fat and protein). Organ effects include deceleration of gastric emptying. | Plasma levels elevated in critically ill, especially in case of enteral feed intolerance/delayed gastric emptying [13,14]. Gastric emptying was inversely related to both fasting and postprandial plasma CCK concentrations, whereas the postprandial increases in plasma CCK were directly related to gastric emptying. | Other factors such as medication and diagnosis may influence. |
| **Peptide YY** | Actions of peptide YY are similar to CCK, however release is from the distal small intestine | Plasma concentrations elevated in critically ill, especially in case of FI/delayed GE [13]. Relations with GE similar to CCK. | - Other factors such as medication and diagnosis may influence. |
| **GLP-1** | Incretin hormone released in response to nutrition. Stimulates insulin and suppresses glucagon secretion and slows gastric emptying | Fasting GLP-1 elevated in critically ill and associated with FI but no clear relation to GE. Evidence inconsistent regarding baseline concentration. [15]. | - No clear relation with endogenous release and GI function |
| **GLP-2** | Stimulates growth of intestinal epithelium | Role during critical illness is uncertain. | - No clear relation with GI function |
| **Ghrelin** | Released from the stomach during fasting, suppressed with nutrition [16,17]. Increases appetite and accelerates gastric emptying. | Plasma levels markedly reduced during fasting in critically ill, patients with FI had lower concentrations of the biologically active (acylated) form [17,18] | - Ghrelin agonists to stimulate GI motility and appetite being evaluated |
| **Motilin** | Released in the small intestine, stimulates gastric emptying. | Limited published data [19] | - More information required regarding mechanism underlying receptor downregulation during prolonged treatment with motilin agonist. |

For sensitivity and specificity, 95% confidence interval is presented in brackets.

* Problems that apply for all listed biomarkers are: no routine use available due to methodology and costs, insufficient data in critically ill

α-SMA - α-smooth muscle actin; CCK – Cholecystokinin; FI – feeding intolerance; GE – gastric emptying; GLP - Glucagon-like peptide; GST - glutathione S-transferase; i-FABP - intestinal fatty acid–binding protein

**References for Table S6**

1. Treskes N, Persoon AM, van Zanten ARH (2017) Diagnostic accuracy of novel serological biomarkers to detect acute mesenteric ischemia: a systematic review and meta-analysis. Intern Emerg Med 12(6):821-836. doi: 10.1007/s11739-017-1668-y
2. Schellekens DH, Grootjans J, Dello SA et al (2014) Plasma intestinal fatty acid-binding protein levels correlate with morphologic epithelial intestinal damage in a human translational ischemia-reperfusion model. J Clin Gastroenterol 48(3): 253–260. doi: 10.1097/MCG.0b013e- 3182a87e3e
3. de Haan JJ, Lubbers T, Derikx JP et al (2009) Rapid development of intestinal cell damage following severe trauma: a prospective observational cohort study. Crit Care 13(3): R86. doi: 10.1186/cc7910
4. Shi H, Wu B, Wan J et al (2015) The role of serum intestinal fatty acid binding protein levels and D-lactate levels in the diagnosis of acute intestinal ischemia. Clin Res Hepatol Gastroenterol 39(3): 373–378. doi: 10.1016/j.clinre.2014.12.005
5. van der Voort PH, Westra B, Wester JP et al (2014) Can serum L-lactate, D-lactate, creatine kinase and I-FABP be used as diagnostic markers in critically ill patients suspected for bowel ischemia. BMC Anesthesiol 14:111. doi: 10.1186/1471-2253-14-111
6. Evennett NJ, Petrov MS, Mittal A, Windsor JA (2009) Systematic review and pooled estimates for the diagnostic accuracy of serological markers for intestinal ischemia. World J Surg 33: 1374-1383. doi: 10.1007/s00268-009-0074-7.
7. Hong J, Gilder E, Blenkiron C et al (2017) Nonocclusive mesenteric infarction after cardiac surgery: potential biomarkers. J Surg Res 211: 21–29. doi: 10.1016/j.jss.2016.12.001
8. Block T, Nilsson TK, Björck M et al (2008) Diagnostic accuracy of plasma bio- markers for intestinal ischaemia. Scand J Clin Lab Invest 68(3): 242–248, doi: 10.1080/00365510701646264
9. Assadian A, Assadian O, Senekowitsch C et al (2006) Plasma D-lactate as a potential early marker for colon ischaemia after open aortic reconstruction. Eur J Vasc Endovasc Surg 31(5):470–474. doi: 10.1016/j. ejvs.2005.10.031
10. Crenn P, Messing B, Cynober L (2008) Citrulline as a biomarker of intestinal failure due to enterocyte mass reduction. Clin Nutr 27:328-339. doi: 10.1016/j.clnu.2008.02.005
11. Shen LJ, Guan YY, Wu XP et al (2015) Serum citrulline as a diagnostic marker of sepsis-induced intestinal dysfunction. Clin Res Hepatol Gastroenterol 39(2):230–236. doi:10.1016/j.clinre.2014.10.002
12. Piton G, Manzon C, Monnet E et al (2010) Plasma citrulline kinetics and prognostic value in critically ill patients. Intensive Care Med 36(4):702-6. doi: 10.1007/s00134-010-1751-6
13. Nguyen NQ, Fraser RJ, Bryant LK et al (2007) The relationship between gastric emptying, plasma cholecystokinin, and peptide YY in critically ill patients. Crit Care 11(6): R132. doi: 10.1186/cc6205.
14. Nguyen NQ, Fraser RJ, Chapman MJ et al (2007) Feed intolerance in critical illness is associated with increased basal and nutrient-stimulated plasma cholecystokinin concentrations. Crit Care Med 35(1):82–88, doi: 10.1097/01.CCM.0000250317.10791.6C
15. Summers MJ, DI Bartolomeo AE, Zaknic AV et al (2014) Endogenous amylin and glucagon-like peptide-1 concentrations are not associated with gastric emptying in critical illness. Acta Anaesthesiol Scand 58(2):235–242. doi: 10.1111/aas.12252
16. Wren AM, Seal LJ, Cohen MA et al (2001) Ghrelin enhances appetite and increases food intake in humans. J Clin Endocrinol Metab 86(12): 5992. doi: 10.1210/jcem.86.12.8111
17. Crona D, MacLaren R (2012) Gastrointestinal hormone concentrations associated with gastric feeding in critically ill patients. JPEN J Parenter Enteral Nutr 36(2):189–196. doi: 10.1177/0148607111413770
18. Nematy M, O’Flynn JE, Wandrag L et al (2006) Changes in appetite related gut hormones in intensive care unit patients: a pilot cohort study. Crit Care 10(1):R10. doi: 10.1186/cc3957
19. Nguyen N, Fraser R, Bryant L et al (2010) M1722 Abnormalities in Plasma Motilin Response to Small Intestinal Nutrient Stimulation in Critically Ill Patients. Gastroenterology 138(5):S-405–S-406, doi: 10.1016/ s0016-5085(10)61865-3.

**Specific aspects and pitfalls for laboratory measurements.**

Here we will discuss strengths and weaknesses of different approaches to estimate absorption of nutrients and their usage in development of biomarkers for gut function.

1. Absorption of nutrients
   1. Whole body balance studies

This is in principle a black-box approach. Whole-body balance studies measure the amount of the nutrient or nutrients that are taken in by the body and subtract the excretion of the same nutrient or nutrients from the body. A positive number indicated a net uptake and a negative number a net loss from the body. The approach mostly used is the nitrogen balance for estimating whole body protein balance, but the principles apply to basically any nutrient. Proteins are the nutrients that carry basically all nitrogen and since nitrogen is easy to measure, this is used to estimate the protein balance. For the nitrogen balance technique there are several limitations. The first one is that all intakes and all losses should be accurately collected and quantified. For patients or volunteers in the hospital or research unit for the period of measurements these can be achieved, although the minimal losses via N2 gas or via loss of hair and skin are missed. For subjects studied in the “wild” this is more problematic. Also, it has been debated how much time is needed to reach a new equilibrium when protein intakes are changed. Some state that this might be a couple of weeks. This makes the use of the technique in short intervention studies less reliable. Also, the technique assumes that all nitrogen from protein breakdown is excreted. This assumption will be compromised in patients with liver and kidney failure. Last but not least, since the approach is a black-box only information of a net uptake or net loss are obtained and no information on absorption and metabolism.

- 1. Studies using marker substances

There are in principle 2 approaches for measuring absorption using marker substrates. 1) Marker substances that are absorbed by the same mechanisms as the nutrient of interest but not being metabolized can be enterally administered and subsequently sampled from blood and/or other body fluids (e.g. 3-O-methyl-glucose) [1]. 2) Isotopic labelling, where the marker substance is metabolized simultaneous with the nutrients but can be distinguished by an isotope label and it appearance and/or metabolism can be measured in the blood (e.g. ^13^C-glucose and D_5_-phenylalanine) [2] or in the breath as ^13^CO_2_ (e.g. ^13^C-sucrose, ^13^C-triolein, ^13^C-octanoate) [3,4,5]. All of these approaches have assumptions and only some have been validated in the critically ill patient. For exemple, ^13^C-octanoate breath test has been shown to correlate well with gastric emptying measured by scintigraphy [5].

Several small studies have demonstrated reduced nutrient absorption in the critically ill patients [3-7].. It needs to be emphasized that „absorption studies“ using labeled glucose or paracetamol absorption assume normal motility if absorption is assessed and normal absorption if motility is assessed.

Nutrient analogues or nutrient labeled with isotope (e.g. 3-O-methyl-glucose or ^13^C-glucose) can be administered with enteral nutrition and subsequently sampled from blood and/or other body fluids to quantify nutrient absorption. Depending on gastric emptying, different results might be obtained when these markers are given intragastrical or intraduodenally. The latter approach will better reflect actual absorption, whereas the former might be more relevant for the actual (bio)-availability of the nutrient for the patient under study. Both strategies carry the underlying assumption that the marker substance will mix with and be handled identically with the reference substance. An inert substance will eventually be excreted un-metabolized, and a kinetic model taking this into account may be used. Non-inert metabolites are also metabolized and this needs to be taken into consideration. One example is the possible utilization of the nutrient in the splanchnic tissues before it appears into the circulation. This can be corrected for by simultaneously infusing a parenteral tracer of the same nutrient. Another example is when using the breath-test, one assumes that the nutrient oxidation is not affected by the critical illness, something that needs to be validated.

When using isotopic labelling, it will be necessary to also monitor the metabolism of the substance, which deserves special concerns but also gives new possibilities. Although absorption can be estimated with these methods, if the patient cannot utilize the nutrients it is not very useful. So, to obtain the full picture utilization might be of interest in some studies. When blood sampling is used, it comes natural to consider a 2-pool model; the sampling pool and the whole-body pool. Anatomically the sampling pool may be looked upon as the circulation blood, but it is important to always remember that the sampling pool is a theoretical construct that is in short term equilibrium with the sampled blood or plasma. This actually may be less than the blood or plasma volume, for example when there is a first pass elimination of the substance from the intestinal lumen through the gut and liver into the general circulation. Sometimes it may be fruitful to consider a 3-pool model, including 3 compartments with different exchangeability. This may be the sampling pool, a free pool that does not equilibrate fast (for example intracellularly) and “bound” pool (for example amino acids being constituents of proteins). Depending on the research question elaborating with multiple pools may be helpful or just confusing. The form of the elimination curve of the plasma concentrations of the marker substance over time will guide if multiple pools are helpful or not. A particular difficulty may be to obtain concentration measurements from other pools than the sampling pool. This may be doable by tissue biopsies, but most often theoretical assumptions and simulations must be used.

A practical example of the usefulness to be able to determine the nutrient utilization is when the effect of feeding upon whole body protein turnover is studied. If nutrition intake is by the parenteral route, the calculations of intake are simple. If nutrition delivery is by the enteral route only a limited fraction will appear in the sampling pool, due to splanchnic extraction. If calculations do not consider this difference of appearance, the calculation of whole body protein turnovers will systematically erroneous and therefore very difficult to interpret. There is a substantial inter-individual variability concerning the time pattern and also the size of the fraction of given nutrition that will appear in the sampling pool. This is true for healthy volunteers and even more so for critically ill patients.

1. Biomarker measurements

Identification of biomarkers for gut function in the critically ill would make diagnosis much easier. Biomarkers can be measured in blood, serum or plasma, or urine and methodology of sampling as well as handling of samples (i.e. derivation and freezing) must be unified within each study. Development of biomarkers can be done by validating previously known proteins or metabolites or can be done by finding novel biomarkers. For the latter often, omics approaches (proteomics or metabolomics) are used. These approaches are very powerful for identifying possible novel biomarkers but due to mainly methodological weaknesses, these always need to be validated in one or more independent cohorts. When biomarkers are used for predicting a clinical status or outcome it is important to validate the specificity and the sensitivity of the predictor.

Different laboratories may have different assays with largely different reference levels for identification of concentrations of the same biomarker. This may lead to largely different reference values and complicate interpretation of results in comparison of other studies [8,9]. This can be solved by standardizing the different assays.

**References for “Specific aspects of laboratory measurements”**

1. Deane AM, Summers MJ, Zaknic AV et al (2011) Glucose absorption and small intestinal transit in critical illness. Crit Care Med 39(6):1282-1288. doi: 10.1097/CCM.0b013e31820ee21f
2. Liebau F, Wernerman J, van Loon LJ, Rooyackers O (2015) Effect of initiating enteral protein feeding on whole-body protein turnover in critically ill patients. Am J Clin Nutr 101(3):549-557. doi: 10.3945/ajcn.114.091934
3. Ali Abdelhamid Y, Cousins CE, Sim JA et al (2015) Effect of Critical Illness on Triglyceride Absorption. JPEN J Parenter Enteral Nutr 39(8):966-972. doi: 10.1177/0148607114540214
4. Burgstad CM, Besanko LK, Deane AM et al (2013) Sucrose malabsorption and impaired mucosal integrity in enterally fed critically ill patients: a prospective cohort observational study. Crit Care Med 41(5):1221-1228. doi: 10.1097/CCM.0b013e31827ca2fa
5. Nguyen NQ, Bryant LK, Burgstad CM et al (2013) Gastric emptying measurement of liquid nutrients using the (13)C-octanoate breath test in critically ill patients: a comparison with scintigraphy. Intensive Care Med 39(7):1238-1246. doi: 10.1007/s00134-013-2881-4
6. Sim JA, Horowitz M, Summers MJ et al (2013) Mesenteric blood flow, glucose absorption and blood pressure responses to small intestinal glucose in critically ill patients older than 65 years. Intensive Care Med 39(2):258-266. doi: 10.1007/s00134-012-2719-5
7. Deane AM, Rayner CK, Keeshan A et al (2014) The effects of critical illness on intestinal glucose sensing, transporters, and absorption. Crit Care Med 42(1):57-65. doi: 10.1097/CCM.0b013e318298a8af
8. Treskes N, Persoon AM, van Zanten ARH (2017) Diagnostic accuracy of novel serological biomarkers to detect acute mesenteric ischemia: a systematic review and meta-analysis. Intern Emerg Med 12(6):821-836. doi: 10.1007/s11739-017-1668-y
9. Reintam Blaser A, Padar M, Tang J, Dutton J, Forbes A (2019) Citrulline and intestinal fatty acid-binding protein as biomarkers for gastrointestinal dysfunction in the critically ill. Anaesthesiology Intensive Therapy 51 (2): 1–10 doi:10.5114/ait.2019.86049

**Table S7. GI motility drugs based on systematic review.**

| **Substance** | **Mechanism of action** | **Indication** | **Recommended dose** | **Side effects** | **Attention** | **Current use in the ICU.**  **Evidence** |
| --- | --- | --- | --- | --- | --- | --- |
| **Domperidone** | Dopamin 2 antagonist. | Gastroparesis  nausea/ vomiting | <30 mg/d orally | Gynecomastia, galactor-rhea, arrhythmia , long QT | Dose limitation 30mg/d  Oral use only | Insufficient data in ICU patients [1] |
| **Metoclopramide** | Dopamin 2 antagonist and nonselective 5-HT_4_ agonist | Gastroparesis  nausea/ vomiting | 10 mg TID i.v.  10 mg once a day in renal failure | Galactorrhea, long QT,cardiac arrhythmia,  extrapyramidal side effects (e.g. dyskinesia) | Contraindicated in Parkinson’s disease,  Tachyphylaxis. Dose reduction in renal failure | Used frequently.  RCTs [2, 3] |
| **Erythromycin** | Motilin agonist | Gastroparesis | 100 mg TID i.v. | Diarrhea,  QT prolongation | Tachyphylaxis  Antibiotic resistance? | Used frequently  RCTs [4, 2, 3, 5] |
| **Selective 5-HT_4_ agonist**s (e.g. prucalopride) | | chronic idiopathic constipation | 1-2 mg/d? | Headache, nausea, and diarrhea | Dose reduction in severe renal and liver failure | Insufficient data in ICU patients [6,7] |
| **Ghrelin agonists (several drugs in studies, e.g.** relamorelin, ulimorelin) | | Gastroparesis  Anorexia | Ulimorelin 600 µg/kg TID in critically ill | Worsening of glycemic control |  | Ulimorelin vs Metoclopramide no dif-ference in therapeutic and side effects  [8] |
| **Neostigmine** | Acetylcholine-esterase inhibitor | Lower GI para-lysis, Ogilvie’s syndrome | 0.5-1.5-(2.5) mg short infusion (0.4-0.8 mg/h) | Bronchospasm,  Bradycardia | Up to 2.5 mg in Ogilvie syndrome, higher doses inhibit motility | Commonly used.  RCTs [1, 9, 10, 11, 12] |
| **Lactulose, Sorbitol** | Osmotic laxatives | Constipation | 20 mg/d | Flatulence, abdominal pain, diarrhea | Contraindicated in Ogilvie’s syndrome | Commonly used, several RCTs [13, 14, 15, 16] |
| **Polyethylene glycol (PEG)** | Osmotic laxative | Constipation | 13-30 mg orally | Flatulence, abdominal pain, diarrhea | Contraindicated in Ogilvie’s syndrome  Attention using potassium enriched preparation in renal failure | The most frequently studied intervention for use as prophylactic bowel regimen. Lower incidence and recurrence of Ogilvie’s Syndrome [13, 14, 15, 16, 17] |
| **Bisacodyl** | Hydragog laxative | Constipation | 10-(20) mg/d rectally | Flatulence, abdominal pain, diarrhea | Contraindicated in Ogilvie’s syndrome | Commonly used but insufficient data for critically ill patients |
| **Naloxone** | Opioid antagonist | Constipation, lower GI paralysis | 3-12 mg TID orally | Symptoms of opioid withdrawal |  | Insufficient data in ICU patients |
| **Alvimopan** | μ -opioid receptor antagonist | Constipation | 12 mg BID orally | Flatulence, abdominal pain, diarrhea | Limited to 15 doses because of cardio-vascular side effects | Currently not in use any more. |
| **Methylnaltrexone** | μ -opioid receptor antagonist | Constipation, lower GI paralysis | 8-12 mg s.c. | Flatulence,  diarrhea,  abdominal perforation |  | Insufficient data in ICU patients, off-label use [18, 19, 20] |
| **Lubiprostone** | Chloride-channel 2 activator | Constipation | 24 µg BID for chronic consti-pation and 8 µg BID for irritable bowel syndrome | Nausea, vomiting, flatulence, diarrhea |  | Insufficient data in ICU patients [21, 22] |
| **Linaclotide** | Guanylate cyclase 2C agonist | Irritable bowel syndrome with constipation | 290 µg OD | Diarrhea, abdominal pain, bloating |  | Insufficient data in ICU patients [23] |

**Legend:** BID – twice per day; GI- gastrointestinal; ICU – intensive care unit; OD – once per day; RCT – randomized controlled trial; TID – three times per day.

**References for Table S7**

1. Fruhwald S, Holzer P. Gastrointestinal motility drugs in critical illness. In :Webb A, Angus D, Finfer S, Gattinoni L, Singer M. Oxford Textbook of Critical Care. 2. Auflage. Oxford: Oxford University Press; 2016: 175-180.
2. Nguyen NQ, Chapman MJ, Fraser RJ, et al. Erythromycin is more effective than metoclopramide in the treatment of feed intolerance in critical illness. Crit Care Med 2007;35(2):483-489.
3. Nguyen NQ, Chapman M, Fraser RJ, et al. Prokinetic therapy for feed intolerance in critical illness: one drug or two? Crit Care Med 2007;35(11):2561-2567.
4. Berne JD, Norwood SH, McAuley CE, et al. Erythromycin reduces delayed gastric emptying in critically ill trauma patients: a randomized, controlled trial. J Trauma 2002;53(3):422-425.
5. Hersch M, Krasilnikov V, Helviz Y, Zevin S, Reissman P, Einav S. Prokinetic drugs for gastric emptying in critically ill ventilated patients: Analysis through breath testing. J Crit Care 2015; 30(3): 655 e7-13
6. Bouras EP, Camilleri M, Burton DD, et al. Prucalopride accelerates gastrointestinal and colonic transit in patients with constipation without a rectal evacuation disorder. Gastroenterology 2001;120(2):354-360.
7. Camilleri M, Piessevaux H, Yiannakou Y, et al. Efficacy and Safety of Prucalopride in Chronic Constipation: An Integrated Analysis of Six Randomized, Controlled Clinical Trials. Dig Dis Sci 2016;61(8):2357-2372.

Heyland DK, van Zanten ARH, Grau-Carmona T, et al. A multicenter, randomized, double-blind study of ulimorelin and metoclopramide in the treatment of critically ill patients with enteral feeding intolerance: PROMOTE trial. Intensive Care Med. 2019 May;45(5):647-656. doi: 10.1007/s00134-019-05593-2.

1. Nee J, Zakari M, Sugarman MA, et al. Efficacy of Treatments for Opioid-induced Constipation: A Systematic Review and Meta-Analysis. Clin Gastroenterol Hepatol 2018.
2. Patanwala AE, Abarca J, Huckleberry Y, et al. Pharmacologic management of constipation in the critically ill patient. Pharmacotherapy 2006;26(7):896-902.
3. van der Spoel JI, Oudemans-van Straaten HM, Kuiper MA, et al. Laxation of critically ill patients with lactulose or polyethylene glycol: a two-center randomized, double-blind, placebo-controlled trial. Crit Care Med 2007;35(12):2726-2731.
4. Vazquez-Sandoval A, Ghamande S, Surani S. Critically ill patients and gut motility: Are we addressing it? World J Gastrointest Pharmacol Ther 2017;8(3):174-179.
5. Masri Y, Abubaker J, Ahmed R. Prophylactic use of laxative for constipation in critically ill patients. Ann Thorac Med 2010;5(4):228-231.
6. Guardiola B, Llompart-Pou JA, Ibanez J, et al. Prophylaxis Versus Treatment Use of Laxative for Paralysis of Lower Gastrointestinal Tract in Critically Ill Patients. J Clin Gastroenterol 2016;50(2):e13-18.
7. de Azevedo RP, Freitas FG, Ferreira EM, et al. Daily laxative therapy reduces organ dysfunction in mechanically ventilated patients: a phase II randomized controlled trial. Crit Care 2015;19:329.
8. Wald A. Appropriate use of laxatives in the management of constipation. Curr Gastroenterol Rep 2007;9(5):410-414.
9. Plummer MP, Reintam Blaser A, Deane AM. Gut dysmotility in the ICU: diagnosis and therapeutic options. Curr Opin Crit Care 2019; 25(2): 138-144
10. Slatkin N, Thomas J, Lipman AG, et al. Methylnaltrexone for treatment of opioid-induced constipation in advanced illness patients. J Support Oncol 2009;7(1):39-46.
11. Sawh SB, Selvaraj IP, Danga A, et al. Use of methylnaltrexone for the treatment of opioid-induced constipation in critical care patients. Mayo Clin Proc 2012;87(3):255-259.
12. Merchan C, Altshuler D, Papadopoulos J. Methylnaltrexone Versus Naloxone for Opioid-Induced Constipation in the Medical Intensive Care Unit. Ann Pharmacother 2017;51(3):203-208.
13. Spierings ELH, Drossman DA, Cryer B, et al. Efficacy and Safety of Lubiprostone in Patients with Opioid-Induced Constipation: Phase 3 Study Results and Pooled Analysis of the Effect of Concomitant Methadone Use on Clinical Outcomes. Pain Med 2017.
14. Cryer B, Drossman DA, Chey WD, et al. Analysis of Nausea in Clinical Studies of Lubiprostone for the Treatment of Constipation Disorders. Dig Dis Sci 2017;62(12):3568-3578.
15. Rey E, Mearin F, Alcedo J, et al. Optimizing the Use of Linaclotide in Patients with Constipation-Predominant Irritable Bowel Syndrome: An Expert Consensus Report. Adv Ther 2017;34(3):587-598. (23)
