# Supplementary material for: Gastrointestinal dysfunction in the critically ill: a systematic scoping review and research agenda proposed by the Section of Metabolism, Endocrinology and Nutrition of the European Society of Intensive Care Medicine
Source: Crit Care. 2020 May 15;24:224. doi: 10.1186/s13054-020-02889-4 (PMC7226709; doi:10.1186/s13054-020-02889-4)
Supplement: Supplementary file 6 — Additional file 6. PRISMA Flow diagrams. This file presents PRISMA (Preferred Reporting Items for Systematic reviews and Meta-Analyses) Flow diagrams for each of 16 systematic reviews separately. [file 13054_2020_2889_MOESM6_ESM.docx]

**Flow diagram topic i**

## Eligibility

## Screening

## Identification

Studies included in study overview
(n = 45)

Records excluded
(n = 597)

Records screened
(n = 642)

Records after duplicates removed
(n =39)

Additional records identified through other sources
(n =7)

Records identified through database searching
(n =674)

**Flow diagram topic ii**

## Identification

## Eligibility

## Screening

Full-text articles assessed for eligibility
(n = 10)

Records excluded
(n = 35)

Records screened
(n =45)

Records after duplicates removed
(n = 0)

Additional records identified through other sources
(n = 6)

Records identified through database searching
(n =39)

**Flow diagram topic iii**

## Screening

## Identification

## Eligibility

Studies included in study overview

(n = 34)

Records excluded
(n =37)

Records screened
(n = 71)

Records after duplicates removed
(n = 0)

Additional records identified through other sources
(n = 19)

Records identified through database searching
(n = 52)

**Flow diagram topic iv**

## Identification

## Eligibility

## Screening

Studies included in study overview
(n = 33)

Records excluded
(n = 41)

Records screened
(n = 74)

Records after duplicates removed
(n = 6)

Additional records identified through other sources
(n = 23)

Records identified through database searching
(n =57)

**Flow diagram topic v**

## Eligibility

## Screening

## Identification

Studies included in study overview
(n = 50)

Records screened
(n = 103)

Records after duplicates removed
(n = 7)

Additional records identified through other sources
(n = 39)

Records identified through database searching
(n = 71)

Records excluded
(n = 53)

**Flow diagram topic vi**

Studies included in study overview
(n = 11)

Records excluded
(n = 23)

Records screened
(n =34)

Records after duplicates removed
(n = 1)

Additional records identified through other sources
(n = 4)

## Identification

## Eligibility

## Screening

Records identified through database searching
(n =31)

**Flow diagram topic vii**

Studies included in study overview
(n = 62)

Records excluded
(n = 130)

Records screened
(n =192)

Records after duplicates removed
(n = 30)

Additional records identified through other sources
(n = 16)

## Identification

## Eligibility

## Screening

Records identified through database searching
(n = 206)

**Flow diagram topic viii**

Studies included in study overview
(n = 21)

Records excluded
(n = 91)

Records screened
(n = 112)

Records after duplicates removed
(n =7)

Additional records identified through other sources
(n = 17)

## Identification

## Eligibility

## Screening

Records identified through database searching
(n =102)

**Flow diagram topic ix**

Records after duplicates removed
(n = 45)

Additional records identified through other sources
(n = 13)

## Identification

## Eligibility

## Screening

Records identified through database searching
(n = 310)

Records excluded
(n = 247)

Records screened
(n =278)

Studies included in study overview
(n = 31)

**Flow diagram topic x**

## Eligibility

## Screening

## Identification

Studies included in study overview
(n = 31)

Records excluded
(n = 550)

Records screened
(n = 581)

Records after duplicates removed
(n = 39)

Additional records identified through other sources
(n = 20)

Records identified through database searching
(n = 600)

**Flow diagram topic xi**

Studies included in study overview
(n = 28)

Records excluded
(n = 182)

Records screened
(n = 210)

Records after duplicates removed
(n =21)

Additional records identified through other sources
(n = 14)

## Identification

## Eligibility

## Screening

Records identified through database searching
(n = 217)

**Flow diagram topic xii**

Studies included in study overview
(n = 40)

Records excluded
(n = 71)

Records after duplicates removed
(n =3)

Additional records identified through other sources
(n = 14)

## Identification

## Eligibility

## Screening

Records identified through database searching
(n = 100)

Records screened
(n =111)

**Flow diagram topic xiii**

Studies included in study overview
(n = 23)

Records excluded
(n = 20)

Records screened
(n =43)

Records after duplicates removed
(n =0)

Additional records identified through other sources
(n = 18)

## Identification

## Eligibility

## Screening

Records identified through database searching
(n =25)

**Flow diagram topic xiv**

Full-text articles assessed for eligibility
(n = 82)

Records excluded
(n = 508)

Records screened
(n = 590)

Records after duplicates removed
(n =22)

Additional records identified through other sources
(n = 49)

## Identification

## Eligibility

## Screening

Records identified through database searching
(n = 563)

**Flow diagram topic xv**

Studies included in study overview
(n = 36)

Records excluded
(n = 29)

Records screened
(n = 65)

Records after duplicates removed
(n =0)

Additional records identified through other sources
(n = 32)

## Identification

## Eligibility

## Screening

**Flow diagram topic xvi**

Records identified through database searching
(n = 33)

Studies included in study overview
(n = 26)

Records excluded
(n = 57)

Records screened
(n = 83)

Records after duplicates removed
(n = 0)

Additional records identified through other sources
(n = 20)

## Identification

## Eligibility

## Screening

Records identified through database searching
(n = 63)

**Flow diagram topic xvi**

## Eligibility

## Screening

## Identification

Studies included in study overview
(n = 10)

Records excluded
(n = 634)

Records screened
(n = 644)

Records after duplicates removed
(n =39)

Additional records identified through other sources
(n = 9)

Records identified through database searching
(n = 674)
